# Cost-effectiveness of prehabilitation prior to elective surgery: a systematic review of economic evaluations

**DOI:** 10.1186/s12916-023-02977-6

**Published:** 2023-07-19

**Authors:** Tanja Rombey, Helene Eckhardt, Jörn Kiselev, Julia Silzle, Tim Mathes, Wilm Quentin

**Affiliations:** 1 Department of Health Care Management, Technische Universität Berlin, Straße des 17. Juni 135, Berlin, 10623 Germany; 2 Department of Anesthesiology and Operative Intensive Care Medicine, Charité – Universitätsmedizin Berlin, Corporate Member of Freie Universität Berlin and Humboldt-Universität zu Berlin, Berlin, Germany; 3 Department of Health Sciences, Fulda University of Applied Sciences, Fulda, Germany; 4 Department for Medical Statistics, University Medical Centre Goettingen, Goettingen, Germany

**Keywords:** Prehabilitation, Cost-effectiveness, Health economics, Systematic review, Evidence synthesis

## Abstract

**Background:**

Prehabilitation aims at enhancing patients’ functional capacity and overall health status to enable them to withstand a forthcoming stressor like surgery. Our aim was to synthesise the evidence on the cost-effectiveness of prehabilitation for patients awaiting elective surgery compared with usual preoperative care.

**Methods:**

We searched PubMed, Embase, the CRD database, ClinicalTrials.gov, the WHO ICTRP and the dissertation databases OADT and DART. Studies comparing prehabilitation for patients with elective surgery to usual preoperative care were included if they reported cost outcomes. All types of economic evaluations (EEs) were included. The primary outcome of the review was cost-effectiveness based on cost–utility analyses (CUAs).

The risk of bias of trial-based EEs was assessed with the Cochrane risk of bias 2 tool and the ROBINS-I tool and the credibility of model-based EEs with the ISPOR checklist. Methodological quality of full EEs was assessed using the CHEC checklist. The EEs’ results were synthesised narratively using vote counting based on direction of effect.

**Results:**

We included 45 unique studies: 25 completed EEs and 20 ongoing studies. Of the completed EEs, 22 were trial-based and three model-based, corresponding to four CUAs, three cost-effectiveness analyses, two cost–benefit analyses, 12 cost–consequence analyses and four cost-minimization analyses. Three of the four trial-based CUAs (75%) found prehabilitation cost-effective, i.e. more effective and/or less costly than usual care. Overall, 16/25 (64.0%) EEs found prehabilitation cost-effective. When excluding studies of insufficient credibility/critical risk of bias, this number reduced to 14/23 (60.9%). In 8/25 (32.0%), cost-effectiveness was unclear, e.g. because prehabilitation was more effective and more costly, and in one EE prehabilitation was not cost-effective.

**Conclusions:**

We found some evidence that prehabilitation for patients awaiting elective surgery is cost-effective compared to usual preoperative care. However, we suspect a relevant risk of publication bias, and most EEs were of high risk of bias and/or low methodological quality. Furthermore, there was relevant heterogeneity depending on the population, intervention and methods. Future EEs should be performed over a longer time horizon and apply a more comprehensive perspective.

**Trial registration:**

PROSPERO CRD42020182813.

**Supplementary Information:**

The online version contains supplementary material available at 10.1186/s12916-023-02977-6.

## Background

### Rationale

Prehabilitation is still a relatively new care concept. It aims at enhancing patients’ functional capacity and overall health status through behaviour change [1] to enable them to withstand a forthcoming stressor [2]. In the surgical context, prehabilitation complements the concept of ‘enhanced recovery after surgery’ (ERAS) and aims to improve surgical outcomes and lower post-operative complication rates [3]. Prehabilitation programmes are delivered preoperatively by a multidisciplinary team and in various settings (e.g. inpatient, outpatient, or at home). Typical modalities include exercise training, promotion of physical activity, nutritional optimisation and psychological support [4], which are provided in addition to elements of ERAS, such as medical optimisation and alcohol or smoking cessation [5].

The potential of prehabilitation is widely recognised. Nevertheless, prehabilitation has not yet been widely adopted by health care systems. Current evidence is still somewhat limited, though much research is still underway to determine the optimal programme types and delivery modalities for different patient populations. Most research activity seems to be in the field of cancer surgery, for example, in an overview of 55 systematic reviews on preoperative prehabilitation, 23 reviews specifically focused on cancer [6]. A likely explanation for this phenomenon is that there is already a large body of evidence demonstrating the positive effects of physical activity on the physical and psychological outcomes of cancer patients [7]. In addition, little is known about the cost-effectiveness of prehabilitation, which is critical for policy-makers considering the implementation of such programmes. By definition, prehabilitation is an approach to reduce healthcare costs [4] and a comprehensive analysis of the value of prehabilitation should incorporate cost outcomes [8].

The aforementioned overview identified only one systematic review on costs [6], but this review focused on nutritional support rather than full prehabilitation programmes [9]. Other reviews that addressed health economic outcomes focused on specific populations [10] or were not systematic reviews [11]. One large systematic review including 178 randomised controlled trials (RCTs) showed that prehabilitation may reduce postoperative length of stay and complications [12], both of which would translate into a cost reduction. However, to our best knowledge, there is currently no comprehensive systematic review on the cost-effectiveness of prehabilitation prior to elective surgery.

### Aim and objectives

The aim of this systematic review was to synthesise the evidence on the cost-effectiveness of prehabilitation programmes for patients awaiting elective surgery compared with usual preoperative care to inform decisions about the implementation of prehabilitation programmes and to guide the design of future rigorous economic evaluations of prehabilitation programmes. More specifically, our objectives were to (1) identify all eligible economic evaluations (EEs), (2) assess their validity and (3) systematically present their characteristics, methods and findings.

## Methods

We followed general methodological guidance on systematic reviews of interventions [13] as well as guidance specific to systematic reviews of EEs [14–16]. Reporting followed the Preferred Reporting Items for Systematic Reviews and Meta-Analyses (PRISMA) 2020 statement [17, 18] and guidance for systematic reviews without meta-analysis [19]. All raw data collected as part of the review are deposited in the Open Science Framework (OSF) [20].

### Registration and protocol

The systematic review was prospectively registered in PROSPERO (CRD42020182813) and we published a protocol [21]. Important protocol changes are reported in Additional file 1: Appendix 1.

### Eligibility criteria

The study in- and exclusion criteria are displayed in Table 1 (from the protocol with additional specifications) [21].

**Table 1:** Review in- and exclusion criteria

PICOS	Inclusion criteria	Exclusion criteria
Population	Patients from any country undergoing elective surgery	Patients undergoing emergency surgery or non-surgical treatments (e.g. chemotherapy)
Intervention	A preoperative prehabilitation programme (any setting), defined as a (set of) intervention(s) aimed at optimising functioning and reducing disability in individuals awaiting surgery. The intervention(s) had to include at least one component of physio- or occupational therapy and at least one in-person meeting between the patient(s) and health care professional(s). The ‘dose’, i.e. the programme’s duration (overall and per session) and frequency, had to be sufficiently long^a^ to have an effect if the patients fully adhered to it	Purely medical/nutritional interventions, an intervention combined with additional postoperative rehabilitation, cognitive behaviour therapy or health counselling/education alone, purely web/app-based prehabilitation programmes
Control	Usual preoperative care as defined by the study authors, i.e. the routine care that patients with a given condition receive in the respective hospital (extended only by the baseline measurements performed as part of the trial)	Another prehabilitation intervention; no comparator
Outcome	Clinical effectiveness and costs, any timeframe for follow-up	Clinical effectiveness only
Study type	Full (i.e. cost–benefit, cost-effectiveness and cost–utility analyses) or partial economic evaluations (i.e. cost-minimization analysis), trial-based^b^ or model-based economic evaluations regardless of their status^c^, cost perspective, publication year, language and type (i.e. full article, conference abstract)	Systematic reviews, simple, non-comparative cost analyses (i.e. studies that only calculated the costs of the intervention), commentaries/letters, animal studies

^a^As judged by a physiotherapist (JK), based on current evidence on exercise efficacy and duration

^b ^We included trial-based economic evaluations based on randomised controlled trials as well as non-randomised studies of interventions, as we expected that the latter would provide valuable additional evidence, e.g. from a real-world setting. If a group in a multi-arm study did not meet the inclusion criteria, we included the study but not the group

^c ^We included ongoing studies, i.e. protocols and registration records, as we were interested in their methods

### Information sources

We searched PubMed, Embase and the Centre for Reviews and Dissemination (CRD) Database on 31/08/2021, which are the most efficient combination of bibliographic databases for systematic reviews of EEs [22]. Furthermore, we searched OADT.org and the DART-Europe E-theses Portal for grey literature and ClinicalTrials.gov and the World Health Organization (WHO) International Clinical Trials Registry Platform (ICTRP) for unpublished and ongoing studies on October 30, 2021. A weekly email alert was created for the search in PubMed (monitored until August 23, 2022). Additionally, we screened the reference lists of included EEs and relevant systematic reviews as well as articles citing the included EEs obtained through Web of Science and Google Scholar. We also contacted the corresponding authors of all included EEs about further relevant EEs.

### Search strategy

The database search strategies consisted of search terms, relating to the population (e.g. ‘preoperative’), the intervention (e.g. ‘exercise’) and study type, i.e. terms to search for economic evaluations (e.g. ‘cost’). Full search strategies for all sources can be found in Additional file 1: Appendix 2.

### Selection process

Records retrieved from databases were deduplicated, screened and managed using EndNote 20 (Clarivate Analytics, Philadelphia (PA), USA). After deduplication, a randomly selected 10% sample of all unique records was screened against the eligibility criteria by two reviewers (TR, HE) independently based on their titles and abstracts. Disagreement was resolved by consensus. As agreement was above 80%, the remaining 90% were screened by one reviewer (TR). We retrieved the full-text articles for all potentially eligible studies as well as for relevant systematic reviews, so that their references could be screened. Each full-text article was screened for eligibility by two reviewers independently who noted reasons for exclusion. Disagreements were resolved by consensus and by consulting a third reviewer (WQ). Last, all study reports were mapped to unique studies as the unit of interest. No automation tools were used in the process.

### Data collection process

Data were extracted into a standardised excel sheet that was piloted by one reviewer (TR). Two reviewers (TR, HE) independently extracted the data of a randomly selected 20% sample of the included completed EEs for calibration. Disagreement was resolved by consensus. As there were no systematic discrepancies, the remaining records were extracted by one reviewer (TR). All outcome data was verified by a second person (JS). We used all documents relevant to the included EEs for data extraction and contacted the study authors via email in case of missing or unclear data. Uncertainties about the methods were only inquired for completed EEs. A reminder email was sent after 2 weeks.

### Data items

A list of all data items and detailed descriptions can be found in Additional file 1: Appendix 3. For ongoing studies, we only extracted the study characteristics and, if published as a protocol, the EE methods. For completed EEs, we also extracted post-operative results data (per group and as the difference between groups) on clinical effectiveness and costs. Costs were reported with their original year and currency as well as converted to 2020 EUR. For conversion, we used the ‘Cochrane Campbell Economic Methods Group and the Evidence for Policy and Practice Information and Coordinating Centre Cost Converter’ (version 1.6) [23]. We only extracted unadjusted data for the last available follow-up point based on intention-to-treat analyses.

### Risk of bias and methodological quality assessment

The risk of bias of trial-based EEs was assessed on outcome-level using the Cochrane risk of bias tool 2 (RoB 2) [24] for EEs based on RCTs and the ROBINS-I tool [25] for EEs based on non-randomised studies of interventions (NRSI). Among other domains, both tools address the risk of reporting bias. Risk of bias figures were created for each outcome domain separately using the robvis application [26]. Methodological quality of trial-based full EEs was assessed using the Consensus on Health Economic Criteria (CHEC) checklist [27]. Model-based EEs were assessed for credibility using the International Society for Pharmacoeconomics and Outcomes Research (ISPOR) checklist [28]. Assessments were performed by two reviewers (TR, HE) independently in a random 20% sample of the included EEs and continued by one reviewer (TR) as agreement was above 80%.

### Effect measures

The review’s primary outcome was the cost-effectiveness from cost–utility analyses (CUAs) based on direction of effect (i.e. reduced costs and/or additional quality-adjusted life year gained). Secondary outcomes were the cost-effectiveness from cost-effectiveness analyses (CEAs), cost–benefit analyses (CBAs), cost-minimisation analyses (CMAs) and cost–consequence analyses (CCAs) based on direction of effect. We calculated effect measures when not reported using risk differences for dichotomous outcomes and mean differences or differences in medians for continuous outcomes. Confidence intervals were extracted when reported. All calculated values are marked as such. All outcomes were reported in disaggregated form in natural units and combined outcome measures, e.g. incremental cost-effectiveness ratios (ICERs), where possible.

### Synthesis methods

We were unable to perform a meta-analysis because the only EEs that were sufficiently homogenous had an unquantifiable overlap in patient populations [29–34] or missed crucial information for data transformation [32, 33]. Therefore, structured narrative synthesis in the form of vote counting based on direction of effects was performed [35]. EEs were grouped by design (model-based vs. trials-based) [16] and analysis type (CUA vs. CEA, CBA, CCA, CMA) to reflect the prioritisation of outcomes.

Results were presented graphically in form of a hierarchical permutation matrix [36]. There were ten possible outcomes for incremental costs (which could be higher, lower or same) and effectiveness (which could be better, poorer, same or inconsistent) corresponding to five result categories: cost-effective, neutral, not cost-effective, unclear; incremental analysis required, and unclear; individual decision required). No formal sensitivity analysis was performed but we discussed the influence of excluding EEs that were of critical risk of bias or insufficient credibility. Descriptive post-hoc subgroup analyses were performed to explore heterogeneity in the EEs’ results arising from differences in populations, interventions, methods, funding source and conflict of interest.

### Assessment of publication bias

To address publication bias, we searched comprehensively for ongoing studies and grey literature and followed up on their status by searching for related publications and contacting the named investigators. In addition, we discussed how the effectiveness results from the included EEs compare to those of clinical effectiveness studies on prehabilitation using an overview of 55 systematic reviews and meta-analyses of RCTs by McIsaac et al. 2022 [6]. Our hypothesis was that the EEs would appear more beneficial if there truly was a publication bias.

## Results

### Study selection

**Fig. 1:**
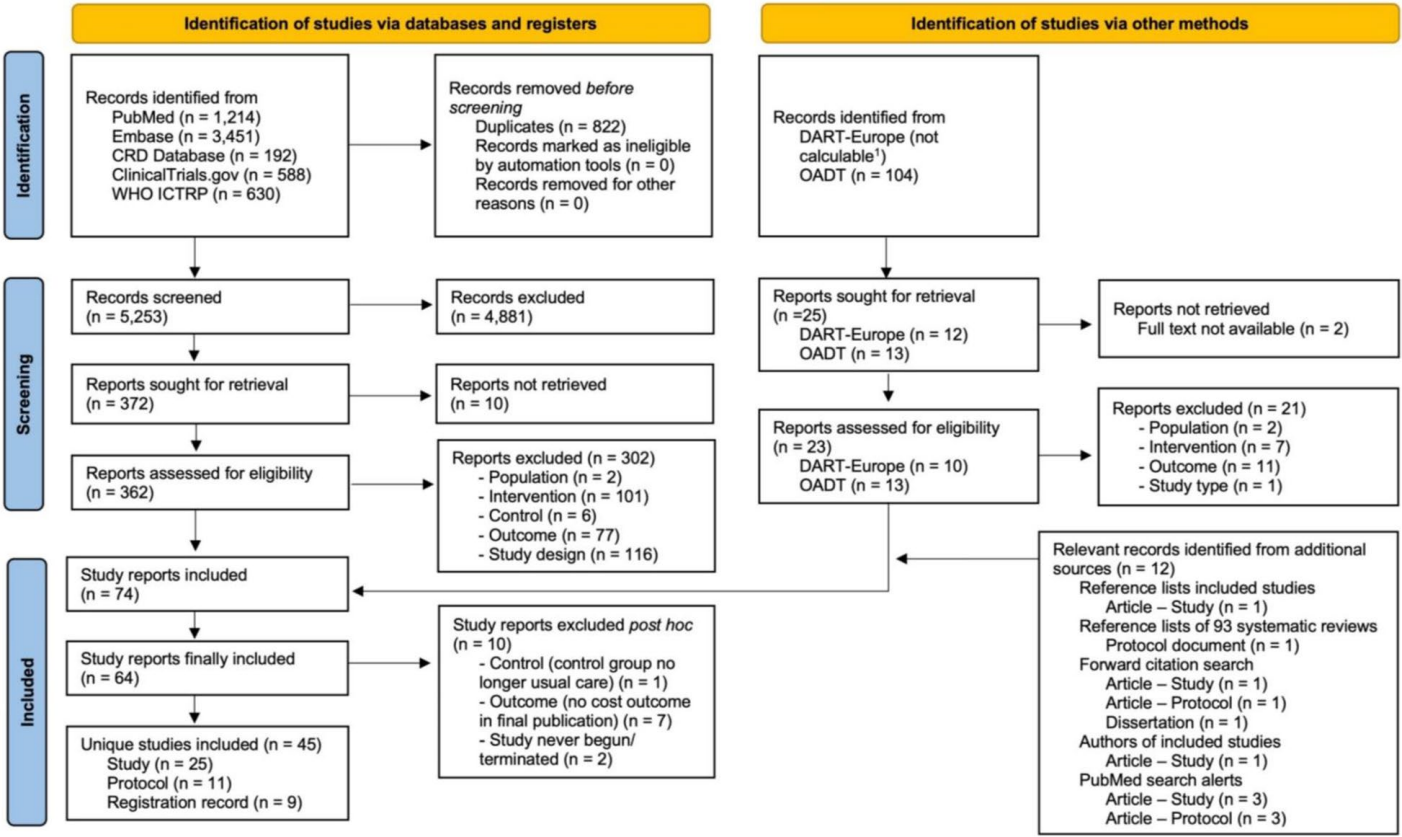
PRISMA 2020 flow diagram of the search and screening process

The study selection process is presented in Fig. 1. In total, 45 unique studies were included: 25 completed EEs [29–34, 37–55] and 20 ongoing studies, of which 11 were published as protocol articles [56–66] and nine as registration records [67–75]. Two completed EEs were only published as conference abstracts [53, 54] and two as dissertations [37, 49]. A total of 54 email enquiries were sent to the study authors, of which 23 were answered (response rate 42.6%). A list of all articles excluded after full-text screening can be found in Additional file 1: Appendix 4, with an additional explanation for close misses and articles excluded post hoc [76–85].

### Characteristics of economic evaluations

The characteristics of the 25 completed EEs are displayed in Table 2. In summary, there were 22 trial-based EEs (13 RCTs and 9 NRSI), and three model-based EEs (2 decision trees and 1 financial projection) corresponding to four CUAs, three CEAs, two CBAs, 12 CCAs and four CMAs. Nine EEs were performed from a mix of a payer and provider perspective, three EEs each from a payer or provider perspective, and one EE from a patient perspective. The perspective remained unclear in the remaining nine EEs.

**Table 2:** Characteristics of the completed economic evaluations

Study ID, main reference^a^	Type and design of analysis	Perspective	Location (city/ cities; country)	Enrolment period^b^	Inclusion criteria (disease(s); type(s) of surgery; criteria for increased perioperative risk)	Population demographics	Number of patients randomised (total (IG vs. CG))
AlShewaier 2016 [37]	CUA; trial-based (RCT)	Unclear	Riyadh; Saudi Arabia	07/2014–01/2015	Isolated ACL injury; ACL reconstruction	Female: 0%, male: 100% Median age: 27 years	84 (39 vs. 45)
Barberan-Garcia 2019 [38]	CCA; trial-based (RCT)	Mix of payer/provider perspective	Barcelona; Spain	02/2013–06/2016	Not specified; major digestive surgery; age > 70 years and/or ASA score ≥ III	Female: 25%, male: 75% Mean age: 71 years	125 (62 vs. 63)
Beaupre 2004 [39]	CMA; trial-based (RCT)	Payer perspective	Edmonton; Canada	Not reported	Non-inflammatory arthritis; primary TKA	Female: 55%, male: 45% Mean age: 67 years	131 (65 vs. 66)
Chen 2022 [40]	CBA; model-based (projection)	Provider perspective	Toronto; Canada	Not applicable (model)	Not specified; major elective intra-cavity surgery; higher-than-average risk, limited physiologic reserve, frailty, deconditioned patient, other indication for prehabilitation with explanation	Not reported	480 (240 vs. 240)
Dholakia 2021 [41]	CEA; model-based (decision tree)	Payer perspective	Not reported; USA	Not applicable (model)	Epithelial ovarian cancer; non-emergent primary debulking surgery; frailty	Female: 100%, male: 0% Age not reported	8830 (4415 vs. 4415)
Englesbe 2017 [29]	CMA; trial-based (NRSI)	Mix of payer/provider perspective	Ann Arbor; USA	IG: 06/2014–12/2015^c^ CG: 07/2006–06/2011	Not specified; major inpatient abdominal and thoracic operative care	Female: 50%, male: 50% Mean age: 60 years	364 (182 vs. 182)
Fernandes 2017 [42]	CUA; trial-based (RCT)	Mix of payer/ provider/patient perspective	Svendborg; Denmark	01/2010–03/2011	Symptomatic osteoarthritis; TKA, THA	Female: 56%, male: 44% Mean age: 67 years	165 (84 vs. 81)
Gao 2015 [43]	CCA; trial-based (NRSI)	Unclear	Chengdu; China	11/2008–06/2011	Lung cancer; lobectomy; > 800 pack-years, quitted smoking < 2 weeks ago, bronchial hyperresponsiveness, impaired lung function	Female: 59%, male: 41% Mean age: 66 years	142 (71 vs. 71)
Gränicher 2020 [44]	CCA; trial-based (RCT)	Payer perspective	Zürich; Switzerland	07/2016–03/2017	Not specified; TKA	Female: 40%, male: 60% Mean age: 67 years	20 (10 vs. 10)
Howard 2019 [30]	CCA; trial-based (NRSI)	Mix of payer/ provider perspective	Ann Arbor; USA	01/2012–12/2017^c^	Not specified; major abdominal surgery	Female: 49%, male: 51% Mean age: 59 years	116 (76 vs. 40)
Huang 2012 [45]	CCA; trial-based (RCT)	Provider perspective	Changhua; Taiwan	01/2008–12/2010	Advanced osteoarthritis; primary TKA	Female: 72%, male: 28% Mean age: 70 years	243 (126 vs. 117)
Koh 2021 [46]	CCA; trial-based (NRSI)	Patient perspective	Singapore; Singapore	IG: 02/2017–03/2020 CG: 04/2016–09/2018	Colorectal cancer; major colectomy; age ≥ 70 years	Female: 44%, male: 56% Median age: 78 years	81 (58 vs. 23)
Lai 2017 [32]	CCA; trial-based (RCT)	Unclear; assumed provider perspective	Chengdu; China	01/2015–12/2015^d^	Non-small cell lung cancer; lung cancer surgery; > 20 pack-years, age > 75 years, BMI > 30, impaired predicted lung function or COPD	Female: 42%, male: 58% Mean age: 64 years	101 (51 vs. 50)
Lai 2019 [33]	CCA; trial-based (RCT)	Unclear; assumed provider perspective	Chengdu; China	01/2018-not reported	Non-small cell lung cancer; lobectomy	Female: 51%, male: 49% Mean age: 64 years	68 (34 vs. 34)
McGregor 2004 [47]	CEA; trial-based (RCT)	Mix of payer/provider perspective	London; UK	Not reported	Not specified; THA	Female: 71%, male: 29% Mean age: 72 years	39 (19 vs. 20)
Mouch 2019 [31]	CMA; trial-based (NRSI)	Mix of payer/provider perspective	Multiple cities in Michigan; USA	01/2014–12/2017^c^	Not specified; various types of surgery; high risk for complications (according to surgeon)	Female: 53%, male: 47% Median age: 70 years	1569 (523 vs. 1046)
Nguyen 2022 [48]	CUA; trial-based (RCT)	Mix of payer/provider perspective	Paris, Clermont-Ferrand; France	10/2012- not reported	Knee osteoarthritis; TKA	Female: 68%, male: 32% Mean age: 69 years	262 (131 vs. 131)
Pham 2016 [49]	CMA; trial-based (RCT)	Unclear; assumed provider perspective	Sudbury; Canada	Not reported	Osteoarthritis; TKA, THA; BMI ≥ 30	Female: 69%, male: 31% Mean age: 64 years	50 (29 vs. 21)
Ploussard 2020 [50]	CCA; trial-based (NRSI)	Mix of payer/provider perspective	Quint-Fonsegrives; France	IG: 01/2018–12/2019 CG: 01/2016–12/2017	Not specified; robot-assisted radical prostatectomy	Female: 0%, male: 100% Mean age: 66 years	350 (194 vs. 156)
Risco 2022 [51]	CCA; trial-based (NRSI)	Provider perspective	Barcelona, Spain	06/2017–12/2019	Not specified; major digestive, cardiac, thoracic, gynaecologic or urologic surgeries; age > 70 years and/or ASA score ≥ III and/or severe deconditioning	Female: 69%, male: 31% Median age 71 years	656 (328 vs. 328)
Tew 2017 [52]	CEA; trial-based (RCT)	Mix of payer/provider perspective	Middlesbrough, Sheffield, York; UK	09/2013–07/2015	Abdominal aortic aneurysms; abdominal aortic aneurysm repair	Female: 6%, male: 94% Mean age: 75 years	53 (27 vs. 26)
Tveter 2020 [53]	CUA; trial-based (RCT)	Unclear; assumed mix of provider, payer and patient perspective	Trondheim, Bergen, Haugesund; Norway	04/2013–06/2015	Carpometacarpal joint osteoarthritis; thumb carpometacarpal joint surgery	Female: 79%, male: 21% Mean age: 63 years	180 (90 vs. 90)
Van Wijk 2020 [54]	CBA; model-based (decision tree)	Not reported	Netherlands (nationwide)	Not applicable (model)	Not specified; pancreatic surgery; low physical fitness, impaired nutritional status, the presence of iron deficiency anaemia, frailty, and/or intoxications	Not reported	Not reported
Wang 2020 [55]	CCA; trial-based (NRSI)	Unclear; assumed provider perspective	Singapore; Singapore	02/2016–10/2017	Hepatocellular carcinoma, colorectal liver metastases; liver resection	Female: 26%, male: 74% Median age: 67 years	104 (70 vs. 34)
Zhou 2017 [34]	CCA; trial-based (NRSI)	Unclear; assumed patient perspective	Chengdu; China	03/2014–06/2015^d^	Primary non-small cell lung cancer; lobectomy; ≥ 20 pack-years, BMI ≥ 28, impaired lung function, COPD/asthma/airway hyper reactivity	Female: 56%, male: 44% Mean age: 59 years	939 (197 vs. 742)

*ACL* anterior cruciate ligament, *ASA* American Society of Anesthesiologists, *BMI* body mass index, *CBA* cost–benefit analysis, *CCA* cost–consequence analysis, *CEA* cost-effectiveness analysis, *CG* control group, *CMA* cost-minimisation analysis, *COPD* chronic obstructive pulmonary disease, *CUA* cost–utility analysis, *IG* intervention group, *NRSI* non-randomised study of interventions, *RCT* randomised controlled trial, *THA* total hip arthroplasty, *TJA* total joint arthroplasty, *TKA* total knee arthroplasty, *UK* United Kingdom, *USA* United States of America

^a ^Further references relating to the included economic evaluations are cited in Additional file 1: Appendix 7

^b ^Extracted from article or registration record (in that order)

^c ^Overlapping population (Michigan, United States)

^d ^Overlapping population (Chengdu, China)

Most EEs were published in the last 10 years and came from Europe (10 EEs), Asia (8 EEs) or North America (7 EEs). The EEs covered a wide range of diseases and surgery types that can be broadly categorised as orthopaedic surgery (9 EEs), cancer surgery (8 EEs), mixed major surgery (6 EEs) and other (2 EEs). In 11 EEs, patients had an increased perioperative risk (e.g. old age or frailty). Sample size ranged from 20 to 8830 patients (median 137). The median proportion of women across the EEs was 53%, and the mean or median age ranged from 59 to 78 years, with one outlier (median age 27 years).

Characteristics and methods of the 20 ongoing EEs are reported in Additional file 1: Appendix 5. All are trial-based EEs, with the majority based on RCTs (18 EEs). There were five CUAs, six CEAs, three EEs using both CUA and CEA, and six EEs with unclear analysis type. In addition to the above continents, two ongoing EEs were from Australia and one from South America. The disease and surgery types were similar to the completed EEs (9 EEs on cancer, 8 EEs on major mixed or major other surgeries and 3 EEs on orthopaedics), though there were slightly more EEs from the field of cardiology and focusing on patients with an increased perioperative risk, and less EEs from the field of orthopaedics.

Information on the completed and ongoing EEs’ funding and conflict of interest can be found in Additional file 1: Appendix 6. Nine EEs did not report any information, one received parts of its funding from a commercial funder [57], one from a private donor [46] and in one, it was unclear [68]. Two EEs declared a relevant conflict of interest [29, 42], as authors were related to companies contracted to organise the prehabilitation.

### Methods of economic evaluations

Detailed information on the methods can be found study-by-study in Additional file 1: Appendix 7 (completed EEs) and Additional file 1: Appendix 8 (ongoing EEs published as protocols). Most completed EEs used a time horizon for effects and costs of 1 month or less (range: 2 weeks to 24 months), with various EEs following patients until discharge and using the costs of hospital stay. No EE discounted effects or costs. Using bootstrapped precision measures (e.g. 95% confidence intervals) was the most common method for calculating uncertainty around the point estimates. Three EEs applied willingness-to-pay thresholds. In summary, with two exceptions [40, 42], few EEs applied comprehensive economic evaluation methods.

### Description of prehabilitation programmes

Characteristics of the completed EEs’ prehabilitation programmes can be found in Table 3. Briefly, in most EEs, the prehabilitation programme was multimodal. All 25 programmes included an exercise element, though the type of training and use of unsupervised sessions varied. Additionally, many included an element of counselling or education (13 EEs) or an element addressing the patients’ nutritional status (11 EEs). The programmes involved various groups of health care professionals, the most common group being physiotherapists (14 EEs). Most programmes were performed in an outpatient (11 EEs) or home setting (8 EEs). The programmes’ overall duration ranged from 3 days to 3 months, with most programmes lasting between 2 and 4 weeks. The frequency of supervised sessions ranged from daily to once per week, with session durations being individual or ranging from 30 to 70 min. Where intensity was reported, we mostly classified it as high, e.g. an 80% of peak work rate for endurance training. Many programmes were not evidence-based. They costed between 100 and 1000 EUR (2020) per patient. Characteristics of the programmes evaluated in the ongoing studies can be found in Additional file 1: Appendix 9.

**Table 3:** Characteristics of the prehabilitation programmes evaluated in the completed economic evaluations

Study ID, main reference	Type and modalities	Involved health care professionals	Setting	Overall duration, frequency and duration per session^a^	Intensity of exercise training^b^	Evidence-based programme	Programme costs in EUR (2020)
AlShewaier 2016 [37]	Unimodal: exercise (resistance, proprioception and balance)	Physiotherapists	Outpatient—hospital	4 weeks, 3x/week, for 45 min	High	Yes	838
Barberan-Garcia 2019 [38]	Multimodal: counselling/education, exercise (endurance), promotion of physical activity	Specialised physiotherapist	Outpatient—hospital	Individual, but min 4 weeks, 1–3x/week, individual session duration	High	Yes	457
Beaupre 2004 [39]	Multimodal: counselling/education, exercise (resistance)	Physiotherapists	Outpatient—community	4 weeks, 3x/week, individual and progressing session duration	High	No	225
Chen 2022 [40]	Multimodal: exercise (endurance, resistance, disease-specific), nutrition (counselling, supplements), psychosocial (stress management), smoking cessation	Kinesiologist or certified exercise physiologist, dietitian, psychologist	Outpatient—hospital (FBP group), home (HBP group)	Individual; min 2 weeks, 2x/week (FBP), 3–5x/week (HBP), for 60 min	Moderate	Yes	798
Dholakia 2021 [41]	Various (model): smoking cessation, stabilising diseases, nutrition (counselling, supplements), exercise (endurance, resistance, inspiratory muscles), psychosocial (stress management), counselling/education, other (social/financial support)	Variable (model)	Variable (model)	Variable (model)	Not applicable	No	Variable (model)
Englesbe 2017 [29]	Multimodal: promotion of physical activity, exercise (inspiratory muscles), nutrition (counselling), psychosocial (stress management), planning of care, smoking cessation	Not reported	Home	Individual; min 2 weeks, 3-7x/week, individual session duration	Not reported	No	80
Fernandes 2017 [42]	Multimodal: exercise (resistance, proprioception and balance)	Physiotherapists	Outpatient—hospital	Not reported but ‘An attendance of 12 sessions or more was considered good compliance’, implying ≥ 6 weeks, 2x/week, for 60 min	Not reported	No	351 (824 when including patient expenses)
Gao 2015 [43]	Multimodal: exercise (inspiratory muscles, endurance)	Professional therapists	Inpatient	3–7 days, 2x/day, for 50–60 min	Not reported	No	246
Gränicher 2020 [44]	Multimodal: exercise (endurance, stretching and flexibility, resistance; individual exercises when indicated), counselling/education	Physiotherapists	Outpatient—hospital	3–4 weeks, 1.25–3x/week, individual session duration	Low to moderate	No	283
Howard 2019 [30]	Multimodal: promotion of physical activity, exercise (inspiratory muscles), nutrition (counselling), psychosocial (stress management), planning of care, smoking cessation	Not reported	Home	Individual; min 2 weeks, 3–7x/week, individual session duration	Not reported	No	80
Huang 2012 [45]	Multimodal: exercise (resistance), counselling/education	Physiotherapists	Home	4 weeks, 7x/week, for 40 min (first session); remaining sessions individual	Not reported	No	20
Koh 2021 [46]	Multimodal: nutrition (supplements), exercise (resistance), counselling/education, drug evaluation, stabilising diseases	Dieticians, physiotherapists	Outpatient—community, hospital^c^	3 weeks, frequency not reported, individual session duration	Not reported	No	Not calculable
Lai 2017 [32]	Multimodal: exercise (inspiratory muscles, endurance)		Inpatient	1 week, 3 + 2 + 1/day, for 45–60 min plus breathing exercises	Not reported	No	Not calculable
Lai 2019 [33]	Multimodal: exercise (inspiratory muscles, endurance)	Specialised nurses, physical therapists	Inpatient	1 week, 3 + 1/day, for 30 min plus breathing exercises	Not reported	No	Not calculable
McGregor 2004 [47]	Unimodal: counselling/education, exercise (not specified)	Not reported	Home	1–3 weeks^c^, unclear frequency, individual session duration	Not reported	No	22
Mouch 2019 [31]	Multimodal: promotion of physical activity, exercise (inspiratory muscles), nutrition (counselling), psychosocial (stress management), planning of care, smoking cessation	Not reported	Home	Individual; min 2 weeks, 3-7x/week, individual session duration	Not reported	No	54
Nguyen 2022 [48]	Multimodal: counselling/education, nutrition (counselling), psychosocial (stress management, anxiety reduction), exercise (resistance, stretching and flexibility, endurance, proprioception and balance)	Physiotherapist, instructor in physical activity, social worker, dietician, psychologist, occupational therapist	Outpatient—hospital, home	8 weeks, 2x/week, for 60 min	Low	Yes	60
Pham 2016 [49]	Multimodal: exercise (stretching and flexibility, resistance, endurance, proprioception and balance), counselling/education	Kinesiologist and/or Human Kinetics graduate student	Outpatient—community	12 weeks, 3x/week, for 40–60 min	Moderate to high	Yes	103
Ploussard 2020 [50]	Multimodal: counselling/education, planning of care, exercise (disease-specific), promotion of physical activity, stabilising diseases, psychological (other), nutrition (counselling, supplements)	Urology nurse, physiotherapist, nurse anaesthetist, oncology nurse specialist, cardiologist (if needed), pneumologist (if needed), psychologist, dietician, urologist	Home	2 weeks^c^, 2–3x/day, for 1 full day, then individual session duration	Not reported	No	231
Risco 2022 [51]	Multimodal: counselling/education, promotion of physical activity, exercise (endurance, resistance), nutrition (counselling, supplements), psychosocial (anxiety reduction, stress management, other)	Anaesthesiologists, physiotherapists, dietitians, psychologists and nurses	Outpatient—hospital	Min 4 weeks, 2-3x/week, for 47 min (endurance) and individual session duration (strength, physical activity)	High (endurance), moderate (strength)	Yes	445
Tew 2017 [52]	Unimodal: exercise (endurance)	Research nurse, physiotherapist	Outpatient—hospital	4 weeks, 3x/week, for 45 min (after first 3 sessions option to do it in 37 min)	High	Yes	1341
Tveter 2020 [53]	Multimodal: counselling/education, other (assistive devices, orthoses), exercise (stretching and flexibility, resistance)	Occupational therapist	Home	12 weeks, 3x/week, individual session duration	Moderate to high	Yes	Not reported
Van Wijk 2020 [54]	Various (model): not reported, but assumedly: exercise^c^, promotion of physical activity, nutrition, stabilizing diseases, alcohol cessation, smoking cessation	Variable (model)	Not reported	Variable (model)	Not reported	No information	1446
Wang 2020 [55]	Multimodal: exercise (inspiratory muscles), nutrition (counselling, supplements), counselling/education, planning of care, other (financial/social support)	Physiotherapist, dietician, case manager	Not reported	2–4 weeks, 5x/week, for 30 min plus breathing exercises	Not reported	No	Not reported
Zhou 2017 [34]	Multimodal: exercise (inspiratory muscles, endurance)	Lung cancer nurse specialists, physiotherapists	Inpatient	1 week, 2 + 3 + 1x/day, for 65–70 min	Not reported	No	Not calculable

*FBP* facility-based prehabilitation, *HBP* home-based prehabilitation

^a ^Referring to exercise element if multiple modalities

^b ^As judged by an experienced physiotherapist

^c ^Information obtained through author contact

### Risk of bias and methodological quality

The results of the assessment of risk of bias and methodological quality of the included studies can be found in Additional file 1: Appendix 10. The majority of RCT-based EEs were judged to be of high risk of bias with the RoB 2 tool. Only one RCT had a moderate risk of bias in all domains [42], and none had a low risk of bias. The main reason for high risk of bias was the absence of a prospective study protocol/registration record. All NRSI-based EE had at least a high, one even a critical risk of bias [34], the main reason being that most EEs did not adequately control for confounding when selecting or analysing patients.

The methodological quality of full trial-based EEs as judged with the CHEC-checklist ranged from 8 to 15 fulfilled items (of 18 to 19 applicable items) and thus can be considered moderate to low. The credibility of model-based EEs as judged with the ISPOR checklist was acceptable in one EE [40], insufficient in another EE [41] and could not be determined due to lack of information in one EE published as a conference abstract [54].

### Results of individual economic evaluations

Table 4 provides an overview of the results of the completed EEs. Smaller values represent a higher benefit unless indicated otherwise. Morbidity refers to the rate of postoperative complications unless indicated otherwise. Detailed cost results can be found in Additional file 1: Appendix 11 including quantities of resource use, unit costs, total costs, incremental cost-effectiveness ratio (ICER) in the original currency and year, and the study authors’ conclusion. Furthermore, adherence and safety/feasibility outcomes can be found in Additional file 1: Appendix 12. Two EEs had adherence rates of less than 35% [48, 51] and in three EEs, drop-out and/or adverse event rates were notably higher in the prehabilitation groups [47, 48, 53].

**Table 4:** Results of completed economic evaluations

Study ID, main reference	Clinical effectiveness (IG vs. CG)	Total costs in EUR (2020) (IG vs. CG)	Cost-effectiveness based on direction of effects^a^	Risk of bias/quality
Results from model-based economic evaluations				
Results from CEAs				
Dholakia 2021 [41]	Mortality: 397/4415 (9.0%) vs. 441/4415 (10.0%); RD^b^ -1.0%	Mean 59,849 vs. 65,304; MD^b^ -5455	Cost-effective	ISPOR-Q: Insufficiently credible
Results from CBAs				
Chen 2022 [40]	Morbidity: 24/240 (10.0%) vs. 16/240 (6.7%); RD^b^ -3.3%	Mean^b^ 3292 vs. 3742; MD^b^ -450	Cost-effective; total cost–benefit: 108,022 EUR (2020)	ISPOR-Q: Sufficiently credible
Van Wijk 2020 [54]	Morbidity: not reported	28,001 vs. 30,242; difference -2,241^c^	Unclear (effectiveness not reported); return of investment: 1.55	ISPOR-Q: Insufficient information
Results from trial-based economic evaluations				
Results from CUAs				
AlShewaier 2016 [37]	QALYs^d^: Median 0.679 (IQR 0.10) vs. 0.573 (0.05); Difference in medians^b^ 0.106	Median 20,790 vs. 19,952; difference in medians^b^ 838	Unclear; incremental analysis required: ICER: 7906 EUR (2020) per QALY gained, but no WTP reported	RoB 2: High CHEC: 13/19 items
Fernandes 2017 [42]	QALYs^d^: Mean 0.66 ± BS SE 0.04 vs. 0.61 ± 0.04^e^; MD 0.04 (95% CI 0.01 to 0.07)	Mean 17,432 ± BS SE 1265 vs. 17,574 ± 1480; MD -142 (95% CI -3952 to 3668) (331^b^ when including patient expenses)	Cost-effective (unclear when including patient expenses); Probability of CEA at a WTP of 40,000 EUR: 84% (approx. 79% when including patient expenses)	RoB 2: Some concerns CHEC: 16/19 items
Nguyen 2022	QALYs^d^: Mean 0.7 ± SD 0.3 vs. 0.6 ± 0.3; MD^b^ 0.1	Mean 15,071 ± SD 7014 vs. 15,472 ± 6309; MD -401	Cost-effective	RoB: Some concerns (QALY), high (costs) CHEC: 9/19 items
Tveter 2020 [53]	QALYs^d^: Not reported per group; difference 0.07^c^	Not reported per group; difference -508^c^	Cost-effective	RoB 2: Some concerns (QALY), high (costs) CHEC: 11/19 items
Results from CEAs				
McGregor 2004 [47]	HrQoL: EQ-5D-3L VAS (0–100%)^d^: mean 75.80 ± SD 14.86 vs. 72.15 ± 22.20^f^; MD^b^ 3.65%, EQ-5D-3L utilities^d^: mean 0.72 ± SD 0.13 vs. 0.60 ± SD 0.31^f^; MD^b^ 0.12	Mean 4148 vs. 5005; MD^b^ -856	Cost-effective	RoB 2: High CHEC: 8/19 items
Tew 2017 [52]	HrQoL: EQ-5D-5L utilities^d^: mean 0.837 vs. 0.760; MD 0.077 (95% CI 0.005 to 0.148)	BS mean 14,269 ± SD 3542 vs. 13,688 ± 3542; BS MD 582 (95% CI -1588 to 2848)	Unclear; incremental analysis required, but ICER not reported	RoB 2: High CHEC: 14/19 items
Results from CCAs				
Barberan-Garcia 2019 [38]	HrQoL: SF-36 PCS^d^: mean 47 ± SD 7 vs. 44 ± 8; MD^b^ 3 SF-36 MCS score^d^: mean 51 ± SD 9 vs. 50 ± 9; MD^b^ 1 Morbidity: 19/62 (30.6%) vs. 39/63 (61.9%); RD^b^ -31.3% Mortality: 1/62 (1.6%) vs. 4/63 (6.3%); RD^b^ -4.7% PROMs: YPAS score^d^: mean 46 ± SD 13 vs. 39 ± 15; MD^b^ 7 HAD total score: mean 6 ± SD 5 vs. 8 ± 6; MD^b^ -2	Mean 5428 (range 1792 to 30,532) vs. 6416 (1587 to 34,521); BS MD -955 (95% CI -3109 to 1033)	Cost-effective	RoB 2: Some concerns (morbidity, mortality), high (PROMs, costs) CHEC: not applicable
Gao 2015 [43]	Morbidity: 12/71 (16.9%) vs. 59/71 (83.3%); RD^b^ -66.4%	Mean 9728 ± SD 1130 vs. 8955 ± 888; MD^b^ 773	Unclear; incremental analysis required, but ICER not applicable for CCA	ROBINS-I: Serious CHEC: not applicable
Gränicher 2020 [44]	PROMs: Lysholm Score^d^: mean 87.1 ± SD 9.0 vs. 69.1 ± 14.9; MD^b^ 18 Lysholm Score pain item^d^: mean 25.0 ± SD 0.0 vs. 16.5 ± 9.1; MD^b^ 8.5 Tegner Activity Scale^d^: mean 3.8 ± SD 0.8 vs. 2.5 ± 0.9; MD^b^ 1.3 Physical function: Stair climbing test, time in seconds: mean 12.58 ± SD 4.64 vs. 13.59 ± 5.30; MD^b^ -1.01 Knee ROM (degrees)^d^: mean 100.5 ± SD 18.7 vs. 103.5 13.7; MD^b^ -3	Mean 3187 vs. 4052; MD^b^ -865	Unclear (inconsistent effectiveness); individual decision required	RoB 2: Some concerns (PROMs, physical function), high (costs) CHEC: not applicable
Howard 2019 [30]	Morbidity: 12/40 (30.0%) vs. 29/75 (38.7%); RD^b^ -8.7% Mortality: 1/40 (2.5%) vs. 1/75 (1.3%); RD^b^ 1.2%	Mean 58,300 ± SD 42,590 vs. 75,248 ± 77,516; MD^b^ (incorporating prehabilitation costs) -16,870	Unclear (inconsistent effectiveness); individual decision required	ROBINS-I: Serious CHEC: not applicable
Huang 2012 [45]	Morbidity: Infection rate: 2/126 (1.6%) vs. 1/117 (0.9%); RD^b^ 0.7% Rate of deep vein thrombosis: 5/126 (4.0%) vs. 3/117 (2.6%); RD^b^ 1.4% PROMs: Pain (VAS): mean 2.4 ± SD 0.7 vs. 2.5 ± 0.6; MD^b^ -0.1 Physical function: Knee ROM (degrees)^d^: mean 76 ± SD 22 vs. 74 ± 20; MD^b^ 2 Ambulation status^d^: 108/126 (85.7%) vs. 95/117 (81.2%); RD^b^ 4.5%	Mean 6726 ± SD 283 vs. 6841 ± SD 241; MD^b^ (incorporating prehabilitation costs) -95	Unclear (inconsistent effectiveness); individual decision required	RoB 2: High CHEC: not applicable
Koh 2021 [46, 86]	Morbidity: 24/58 (41.4%) vs. 11/23 (47.8%); RD^b^ -6.4% Mortality^g^: 0/58 (0%) vs. 0/23 (0%); RD^b^ 0%	Not reported per group; MD -2584	Cost-effective	ROBINS-I: Serious CHEC: not applicable
Lai 2017 [32]	Morbidity: 5/51 (9.8%) vs. 14/50 (28.0%); RD^b^ -18.2%	Mean 7677 ± SD 1374 vs. 8608 ± 2482; MD^b^ -931	Cost-effective	RoB 2: High CHEC: not applicable
Lai 2019 [33]	Morbidity: 4/34 (11.8%) vs. 12/34 (35.3%); RD^b^ -23.5%	Median 10,456 (IQR 9683 to 11,339) vs. 11,285 (10,544 to 13,340); Difference in medians^b^ -830	Cost-effective	RoB 2: High CHEC: not applicable
Ploussard 2020 [50]	Mortality: 0/194 (0%) vs. 1/156 (0.6%); RD^b^ -0.6%	Mean 2904 vs. 3282; MD^b^ -379	Cost-effective	ROBINS-I: Serious CHEC: not applicable
Risco 2022 [51]	Morbidity: Comprehensive complications index: mean 15.1 ± SD 17.1 vs. 16.6 ± 16.9; MD^b^: -1.5	Mean 7288 vs. 7142; MD 145	Unclear; incremental analysis required, but ICER not applicable for CCA	ROBINS-I: Serious CHEC: not applicable
Wang 2020 [55]	Morbidity: 21/70 (30.0%) vs. 18/34 (52.9%); RD -22.9% Mortality: 1/70 (1.4%) vs. 1/34 (2.9%); RD^b^ -1.5% PROMs (in subsample of *n* = 33 vs. *n* = 24): FACT-Hep score^d^: median 152 (range 102 to 179) vs. 148 (66 to 175); differences in medians^b^ 4	Median 6138 (IQR 4590 to 8833) vs. 7349 (5328 to 11,026); difference in medians -1210	Cost-effective	ROBINS-I: Serious CHEC: not applicable
Zhou 2017 [34]	Morbidity: 36/197 (18.3%) vs. 194/742 (26.1%); RD^b^: -7.8%	Not calculable in EUR (2020) as original currency not reported; original values: mean 7131.8 ± SD 2316.6 vs. 77,266.4 ± 1615.0; MD^b^ -134.60	Cost-effective	ROBINS-I: Critical CHEC: not applicable
Results from CMAs				
Beaupre 2004 [39]	Not applicable	Mean 1285 ± SD 1196 vs. 1283 ± 1329; MD 2	Not cost-effective	RoB 2: High CHEC: not applicable
Englesbe 2017 [29]	Not applicable	Provider perspective: median 16,900 (IQR 10,162 to 30,365) vs. 23,091 (14,993 to 39,017); difference in medians^b^ -6191 Payer perspective: median 19,216 (IQR 12,122 to 33,840) vs. 24,519 (17,057 to 37,243); difference in medians^b^ -5303	Cost-effective	ROBINS-I: Serious CHEC: not applicable
Mouch 2019 [31]	Not applicable	Mean 24,435 ± SD 20,024 vs. 26,903 ± 24,935; MD^b^ -2468	Cost-effective	ROBINS-I: Moderate CHEC: not applicable
Pham 2016 [49]	Not applicable	Reported only for a subset of patients (5/29 vs. 11/21): mean 5081 ± SD 298 vs. 5152 ± 656; MD^b^ -71	Cost-effective	RoB 2: High CHEC: not applicable

*BS* bootstrapped, *CBA* cost–benefit analysis, *CCA* cost–consequence analysis, *CEA* cost-effectiveness analysis, *CG* control group, *CHEC* Consensus on Health Economic Criteria, *CMA* cost-minimisation analysis, *CUA* cost–utility analysis, *EQ-5D-3L* EuroQoL 5 dimensions 3 levels, *EQ-5D-5L* EuroQoL 5 dimensions 5 levels, *FACT-Hep* Functional Assessment of Cancer Therapy—Hepatobiliary, *HAD* Hospital Anxiety and Depression Scale, *IG* intervention group, *ICER* incremental cost-effectiveness ratio, *ISPOR-Q* International Society for Pharmacoeconomics and Outcomes Research Questionnaire, *IQR* interquartile range, *MD* mean difference, *PROMs* patient-reported outcome measures, *QALY* quality-adjusted life-year, *HrQoL* health-related quality of life, *RD* relative difference, *RoB 2* revised Cochrane risk-of-bias tool for randomised trials, *ROBINS-I* tool for assessing risk of bias in non-randomised studies of interventions, *ROM* range of motion, *SD* standard deviation, *SF-36* Short Form 36, *VAS* visual analogue scale, *YPAS* Yale Physical Activity Scale

^a ^‘Cost-effective’ if better effectiveness and same/lower costs, or same effectiveness and lower costs; ‘unclear’ if better effectiveness and higher costs, or same effectiveness and same costs, or inconsistent/poorer effectiveness and lower costs; ‘not cost-effective’ if same effectiveness and higher costs, or poorer effectiveness and same/higher costs

^b ^Calculated by review authors

^c ^Measure of central tendency (mean, median) not reported

^d ^Higher values indicating higher benefit

^e ^When missing values were imputed by linear trend at point and adjusted for baseline EQ-5D-3 L scores

^f ^Values were obtained through author contact

^g ^Survival was also calculated but not mentioned in the methods as an outcome

### Results of synthesis

**Fig. 2:**
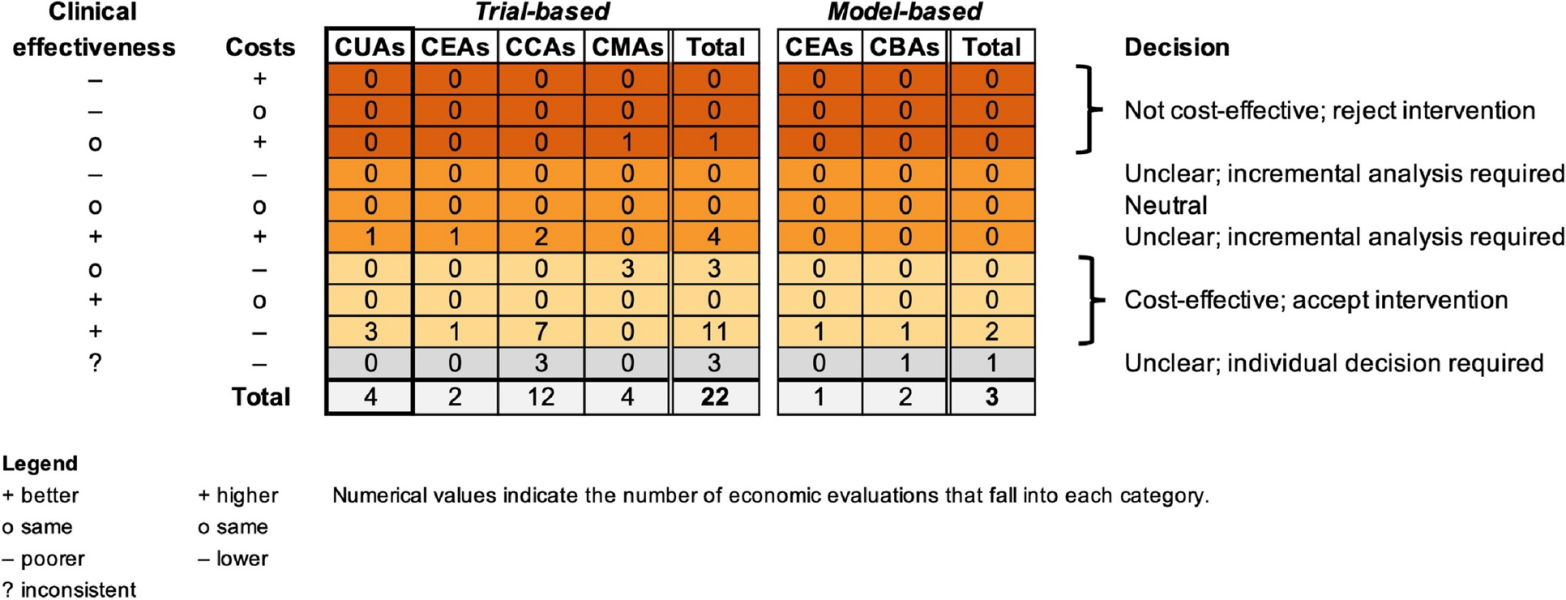
Hierarchical permutation matrix presenting the results vote counting based on direction of effects

Four trial-based EEs [37, 42, 48, 53] reported data on the primary outcome, i.e. cost-effectiveness based on CUA (Fig. 2, thick bordered column). Based on direction of effects, three CUAs (75%) fell into the cost-effective category, and one fell into the category ‘unclear; incremental analysis required’. The ICER of the latter study was 7906 EUR (2020) per quality-adjusted life year (QALY) gained, which is likely acceptable under common willingness-to-pay (WTP) thresholds [37].

Three model-based and 18 trial-based EEs reported on the secondary outcomes (Fig. 2), i.e. cost-effectiveness based on other types of EEs, respectively. Based on direction of effects, two model-based EEs fell into the cost-effective category [40, 41], but one was judged insufficiently credible [41]. The remaining model-based EE fell into the category ‘unclear; individual decision required’ [54]. Of the trial-based EEs, 11 fell into the cost-effective category [29, 31–34, 38, 46, 47, 49, 50, 55], one of which was judged to be of critical risk of bias [34], three into the category ‘unclear; incremental analysis required’ [43, 51, 52], three into the category ‘unclear; individual decision required’ [30, 44, 45], and one, a CMA with a difference in total costs of + 2 EUR (2020), into the not cost-effective category [39].

Overall, 16/25 (64.0%) EEs found prehabilitation cost-effective based on direction of effects, (14/23; 60.9% when excluding the EEs of insufficient credibility/critical risk of bias [34, 41]), in 8/25 (32.0%) it was unclear, and one EE (4.0%) found prehabilitation not cost-effective [39]. Descriptive post hoc subgroup analyses revealed heterogeneity in the cost-effectiveness results depending on the population, intervention and methods, but not on conflict of interest and funding source (Additional file 1: Appendix 13). Briefly, cost-effectiveness was more frequently observed in EEs of cancer patients, patients with a high perioperative risk, multimodal programmes, home-based or inpatient prehabilitation, shorter programmes, low-cost programmes and EEs taking a mix of payer/provider perspective.

### Publication bias

There was a relevant risk of publication bias regarding the included completed EEs. Firstly, the review had initially included 74 study reports belonging to 54 unique studies. However, ten reports referring to nine unique studies were excluded post hoc [76–85] (see Additional file 1: Appendix 4), of which four protocols [77–80], one registration record [85] and a conference abstract [81] referred to studies that no longer reported on costs in the study publication [87–95]. The authors of two studies confirmed that no economic evaluation was performed [78, 80]. The remaining authors did not respond.

In comparison to the results of the overview by McIsaac et al. 2022 [6], the included EEs on cancer surgery showed more beneficial results regarding morbidity [32–34, 43, 46, 55] and mortality [41, 50, 55], and the included EEs on orthopaedic surgery showed more beneficial results on health-related quality of life (HrQoL) [37, 42, 47, 48, 53]. Apart from that, results were comparable but, overall, the included EEs’ results appear more beneficial suggesting a risk of publication bias.

## Discussion

This is the first comprehensive systematic review on the cost-effectiveness of prehabilitation prior to elective surgery including 25 completed and 20 ongoing EEs. Using vote-counting based on direction of effects, the majority of completed EEs found prehabilitation cost-effective, including three CUAs, and only one EE favoured usual care. However, most EEs were of high risk of bias and/or low methodological quality, and we identified a relevant risk of publication bias. Furthermore, the included EEs were heterogeneous in their population, intervention and methods. Therefore, our results should be interpreted with caution. An update of this review might lead to more definite evidence, as it should include at least eight more completed CUAs [58, 59, 62–66].

Cost-effectiveness depended on the population and intervention, with certain groups (e.g. cancer- or high-risk patients) and programmes (e.g. shorter, home-based prehabilitation) resulting more frequently in benefit. Among the included EEs, there was a high variability in populations, whose underlying diseases and surgeries differed in concept (e.g. restoration in orthopaedic surgery and cure in cancer surgery). It is possible that for orthopaedic patients, the restoring character of the surgery might be the crucial element in the recovery of both groups, although the modalities of prehabilitation may also serve as a conservative therapy option for certain orthopaedic patients, delaying or even eliminating the need for surgery [49, 53]. Of course, for other patient groups, cure through prehabilitation is not possible, e.g. for cancer patients whose disease cannot be improved in itself by prehabilitation. Lastly, it might be more (cost-)effective to focus on patients with low functional capacity [96] who are at high-risk for adverse perioperative outcomes because of factors such as old age, relevant co-morbidities [4] and frailty [97], as these patients much room for preoperative improvement.

Our review also showed great variability in the programme modalities, ‘dose’ (i.e. frequency, intensity and duration) and delivery settings. As a result, the programme costs ranged from below 100 EUR (2020) per patient in six (mainly home-based) EEs [29–31, 45, 47, 48] to above 1000 EUR (2020) in two EEs [52, 54]. Although prehabilitation is usually defined as a multi-modal approach, it is not yet clear what intervention designs are most effective and whether they in fact need to be multimodal [6]. For example, in certain indications, a unimodal intervention, such as preoperative breathing exercises, would likely be less costly and hence could turn out to be more cost-effective.

The dose–response relationship of prehabilitation programmes is a crucial aspect for programme effectiveness and depends largely on the length of the preoperative period available for prehabilitation. This again depends on the underlying diseases and how fast these are progressing, i.e. patients with slowly progressing diseases, such as osteoarthritis, can generally wait longer than those with more rapidly progressing diseases, such as most cancer types, who should often be operated within a few weeks following diagnosis [98]. However, cancer patients undergoing neoadjuvant treatment before surgery may be ideal candidates for prehabilitation [99, 100]. Similarly, the waiting period for patients on organ transplant lists may present a window of opportunity to implement a prehabilitation programme [101], and waiting lists in general may aid the early identification of eligible patients [102].

The dose of prehabilitation is also determined by the intensity of individual sessions which must be sufficiently high to have an effect while being tolerable for the target population [103]. Although there were few adverse events directly related to prehabilitation, some EEs reported that patients from the intervention group dropped out due to high-intensity [32–34]. Programmes must be designed in a way to facilitate high adherence rates and thus cost-effectiveness [104]. For instance, offering home-based options may reduce issues regarding transportation, which was found to be a central barrier to adherence to prehabilitation [105]. Though not considered specifically in this review, telemedicine is likely to play an important role in the provision of prehabilitation as well.

### Limitations

Some limitations on review and study level apply. First, we could not perform a meta-analysis but had to resort to narrative synthesis in the form of vote counting based on direction of effects. This synthesis method does not provide any information on the magnitude of effects, nor does it account for the EEs’ sample sizes [106]. Second, the review’s broad inclusion criteria led to a large number of included articles that we coined ‘EEs’ for the purpose of the review. However, most of them were trial reports including cost outcomes which understood themselves as pilot and/or feasibility trials and thus did not apply comprehensive EE methods. Third, as there currently is no universally recognised definition of prehabilitation [6] nor common concepts, procedures or measurements [4], the definition of the prehabilitation elements varied between the EEs. The definition of usual care also varied across EEs. For instance, advice on physical activity and smoking cessation were included as standard care in some EEs [38, 50, 51], while those aspects were part of prehabilitation in other EEs [29–31, 40, 41, 54]. Lastly, characteristics of health systems, such as the type of financing (public vs. private) and organisation of care (centralised vs. decentralised), play a crucial role in programme delivery and cost justification. As we did not formally assess the generalisability and transferability of our results to different health systems, we recommend policy-makers interested in implementing prehabilitation to conduct a health technology assessment (HTA) for their government.

Limitations on the study level included the high risk of bias and low methodological quality of the included EEs. However, the exclusion of the two EEs judged to be insufficiently credible/of critical risk of bias only had a small effect on the results. Furthermore, there was a high risk of publication bias associated with trial-based EEs. Trial-based EEs are by nature prone to a specific form of publication bias, namely conduct bias [107], meaning they are not published because they were never performed in the first place, e.g. when the underlying trial was ‘inconclusive’ or had negative results. Although an intervention that is less effective but cheaper than the control may still be cost-effective, it is generally not acceptable from an ethical and quality of care perspective to replace usual care with a less-effective intervention.

### Implications for practice and policy

Owing to the limitations described above, our results should be interpreted with caution. As many EEs were based on prospective trials, decision-makers must also consider the possibility that there was a motivational bias among the participants and that the cost-effectiveness of prehabilitation may be lower under ‘real world’ circumstances. Before implementing prehabilitation into routine care, decision-makers should assess potential barriers and facilitators [108, 109], which may differ between health systems and stakeholders, or even individuals. For example, qualitative studies found that group prehabilitation was perceived both as a barrier and facilitator [110, 111]. In their framework for prehabilitation services, Bates et al. 2020 list several considerations for the implementation of prehabilitation, including to involve patients when designing the prehabilitation programme [112].

Finally, decision-makers must determine which patient population(s) should receive prehabilitation and establish screening pathways, accessibility to the programme and strategies to ensure sustainability [113]. This involves performing a budget impact analysis, including the one-time investments into infrastructure (e.g. prehabilitation centres) as well as the running costs for the provision of prehabilitation and maintenance of the infrastructure. Although many EEs found that prehabilitation paid off during the index hospitalisation, the pervasive shortage of health care professionals [114] may hinder implementation of prehabilitation.

### Implications for future research

First, future research should address the knowledge gaps discussed above, i.e. which populations benefit most and what the optimal prehabilitation programme for those populations is. If a broadly defined population is included in a clinical trial, it is recommended to consider pre-specified subgroup analyses for economic evaluation [115]. To ensure added value, new clinical research should consider the existing evidence [116] as well as involve patients and stakeholders in all phases of research [117], e.g. when designing the prehabilitation programme [118]. Ideally, these efforts would result in a clinical practice guideline for prehabilitation, the first step of which was taken by Tew et al. 2018 with a guideline on preoperative exercise training in patients awaiting major noncardiac surgery [119].

Second, future research should address the shortcomings of existing EEs. Common issues included inadequate reporting, short time horizons, and the use of limited perspectives. Reporting guidelines are intended to support authors and increase the accuracy and transparency of reporting, but they are frequently used inappropriately, including those for EEs [120]. In our review, reporting guidelines for EEs seemed to have been under-used, as none of the full EEs published as full-text articles after 2013 [37, 40–42, 48, 52] reported following the Consolidated Health Economic Evaluation Reporting Standards (CHEERS) [121], which is applicable to both trial- and model-based EEs. A possible reason is that only two trial-based EEs were published as separate full-text articles [38, 42]. Hence, we recommend that authors publish full EEs as separate articles and follow the latest version of the CHEERS checklist [122].

Many EEs had a short time horizon of 1 month or less. However, as argued by Grocott and Ludbrook 2019, ‘it is plausible that improved fitness arising from prehabilitation might have a further lingering positive impact on the need for later care’ [123]. Such an impact can only be detected using a longer time horizon but, in our review, only five EEs [39, 41, 42, 48, 53] had a time horizon of 12 months or more. To determine an adequately long-time horizon, we recommend authors to consult guidelines from their national HTA institutes and by the ISPOR [115]. On a closely-related matter, many EEs applied limited perspectives, such as the provider perspective, with the hospital being the provider, and therefore did not consider post-discharge or out-of-hospital resource use. None of the included EEs applied a full societal perspective including costs from other sectors, e.g. productivity loss. When this is not feasible, we suggest that authors adopt a comprehensive health sector perspective including all relevant payers and providers. For example, EEs may consider improved access for other patients through freed-up capacity, e.g. due to earlier discharge of prehabilitated patients [124]. In summary, future EEs should be performed over a longer time horizon and apply a more comprehensive perspective.

### Update of the review

We plan to update the review upon publication of our own economic evaluation [59] in 2025/26 by re-running the search strategies modified only by adding the MeSH/Emtree term ‘Preoperative Exercise’.

## Conclusions

We found some evidence that prehabilitation for patients awaiting elective surgery is cost-effective compared to usual preoperative care. Cost-effectiveness based on direction of effect was more frequently observed for cancer patients, patients with a high perioperative risk and for low-cost (shorter or home-based) programmes. However, the results should be interpreted with caution as most EEs were of high risk of bias and/or low methodological quality, and we suspect a relevant risk of publication bias. Future research should address clinical knowledge gaps surrounding prehabilitation, e.g. which populations benefit most, as well as the shortcomings of existing EEs, e.g. by adopting a societal perspective.

## Supplementary Information

**Additional file 1: App1.** Important changes made to the protocol. **App2.** Search strategies. **App3.** Data items. **App4.** List of excluded studies. **App5.** Characteristics of ongoing economic evaluations. **App6.** Funding and competing interest of included economic evaluations. **App7. **Methods of completed economic evaluations. **App8.** Methods of ongoing economic evaluations with a published protocol. **App9. **Description of prehabilitation programmes in ongoing studies. **App10. **Risk of bias and methodological quality of included economic evaluations. **App11. **Detailed costs results of included economic evaluations. **App12. **Results of adherence and safety outcomes. **App13. **Results of descriptive post-hoc subgroup analyses to explore heterogeneity in cost-effectiveness results.

## Data Availability

All raw data collected as part of the review are deposited in the Open Science Framework (OSF) [20].

## Abbreviations

CBA: Cost-benefit analysis
CCA: Cost-consequence analysis
CEA: Cost-effectiveness analysis
CHEC: Consensus on Health Economic Criteria
CHEERS: Consolidated Health Economic Evaluation Reporting Standards
CMA: Cost-minimisation analysis
CRD: Centre for Reviews and Dissemination
CUA: Cost-utility analysis
EE: Economic evaluation
ERAS: Enhanced recovery after surgery
HrQoL: Health-related quality of life
HTA: Health technology assessment
ICER: Incremental cost-effectiveness ratio
ICTRP: International Clinical Trials Registry Platform
ISPOR: International Society for Pharmacoeconomics and Outcomes Research
NRSI: Non-randomised study of an intervention
PRISMA: Preferred Reporting Items for Systematic Reviews and Meta-Analyses
QALY: Quality-adjusted life year
RCT: Randomised controlled trial
ROB: Risk of bias
ROBINS-I: Risk of bias in non-randomised studies of interventions
WHO: World Health Organization
