# Supplementary material for: Cost-effectiveness of prehabilitation prior to elective surgery: a systematic review of economic evaluations
Source: BMC Med. 2023 Jul 19;21:265. doi: 10.1186/s12916-023-02977-6 (PMC10354976; doi:10.1186/s12916-023-02977-6)
Supplement: Supplementary file 1 — Additional file 1: App1. Important changes made to the protocol. App2. Search strategies. App3. Data items. App4. List of excluded studies. App5. Characteristics of ongoing economic evaluations. App6. Funding and competing interest of included economic evaluations. App7. Methods of completed economic evaluations. App8. Methods of ongoing economic evaluations with a published protocol. App9. Description of prehabilitation programmes in ongoing studies. App10. Risk of bias and methodological quality of included economic evaluations. App11. Detailed costs results of included economic evaluations. App12. Results of adherence and safety outcomes. App13. Results of descriptive post-hoc subgroup analyses to explore heterogeneity in cost-effectiveness results. [file 12916_2023_2977_MOESM1_ESM.docx]

## Additional file 1 - Appendices

### Appendix 1 – Important changes made to the protocol

| **Domain** | **Change** | **Explanation** |
| --- | --- | --- |
| **Research team** | The research team was extended to involve a physiotherapist (JK), an occupational therapist (JS) and a statistician with expert knowledge in evidence synthesis of economic evaluations (TM). | Additions to the team were made to increase the team’s methodological and content expertise. |
| **Dates** | The completion date changed from 31 March 2021 to 30 April 2023. | Due to the COVID-19 pandemic, the main project was haltered for several months and extended to August 2024. |
| **Dates** | The date for the update changed from summer 2022 to 2025/26. | The date for the update was delayed so that it will include our own economic evaluation (see Schaller et al. 2022. DOI: 10.1186/s13063-022-06401-x; expected date of publication 2025).  We plan to further extend the research team for the planned update to include an anaesthesiologist and a patient representative. |
| **Databases** | We changed the provider for Embase from Embase to Ovid in the latest search. | We had to change the provider for Embase as the team’s licence for Embase (via Embase) run out after the first search. For the update search in August 2021 Embase via Ovid was used.  Due to the change, no alert was created for Embase (but only for PubMed).  32 records that were previously identified in Embase via Embase could not be retrieved again. However, all of these had been excluded after title/abstract screening. An additional 137 records of articles published before 2020 were found, which had not been retrieved in the Embase via Embase search, 6 of which were included after title/abstract screening (5 conference abstracts) and 2 after full-text screening. |
| **Search** | We updated the search in August 2021 but not again upon completion of the review. | We did not want to further delay the review’s completion date and plan to update the searches when updating the whole review. |
| **Data extraction** | We did not strictly calculate agreement between reviewers following calibration (as originally planned) but evaluated if and which items need to be changed based on subjective evaluation. | Focusing on agreement only was found not to be feasible for some disagreements did not really present essential differences while others did. |
| **Data extraction** | Missing data required for synthesis were imputed for simple calculations and marked as such. | We decided to impute data required for synthesis (e.g., mean or relative differences) to increase the number of synthesisable EEs and thereby the meaning of the review’s results. |
| **Data items** | Instead of extraction the conclusion of all EEs, we only extracted the conclusions of completed, full EEs to use it for the assessment with the CHEC checklist. | We did not longer see any use in providing readers with the EEs’ conclusions given that conclusions are known to have a ‘spin’ problematic, i.e., focusing on positive results only. |
| **Outcomes** | We specified our definition of patient-relevant endpoints to include the following groups: Health related quality of life (HrQoL), morbidity, mortality, patient-reported outcome measures other than HrQoL and function. | We chose these outcomes as we believed that these are most meaningful to patients. This will be re-evaluated once the review team includes a patient-representative. |
| **Outcomes** | We specified our definition of outcomes to include only outcomes measured after the surgery and in both groups. | Some studies reported outcomes following prehabilitation but before surgery and/or for the prehabilitation group only. We did not find these outcomes meaningful in comparative evaluation of prehabilitation (per definition demanding that it is followed by surgery). |
| **Risk of bias assessment** | We assessed the risk of bias per outcome group and not on study level only (as originally planned). | The change was made following guidance for the ROB 2 and ROBINS-I tool. |
| **Assessment of methodological quality** | We did not apply the whole ISPOR checklist to the model-based EEs, but only assessed their credibility (11 questions) and assumed that all model-based EEs were relevant. | We did not assess the model-based EEs because this would have required us to refer to a specific health care system (not intended for this review). |
| **Assessment of publication bias** | We added another method to assess publication bias: Comparison of the effectiveness results from the included EEs to those of systematic reviews on the clinical effectiveness of prehabilitation. | We wished to increase our efforts of detecting publication bias in this review, given it is known to be problematic for EEs. |
| **Data presentation** | We did not report the primary outcome broken-down to change in health-related quality of life and length of life. | None of the included EEs reported the times spent in health states not reported separately. |
| **Data presentation** | Data were presented using a hierarchical permutation matrix based on direction of effects instead of cost-effectiveness planes. | The included EEs were not sufficiently homogenous to use cost-effectiveness planes. |
| **Data synthesis** | We added a narrative synthesis method, namely vote-counting based on direction of effects, to summarise the EEs results. | We could not perform a meta-analysis but felt that it would increase the usability of the review’s results to decision-makers if the EEs results were narratively synthesized (in addition to reporting them in table format). |
| **Assessment of confidence in cumulative evidence** | We did not perform of a full GRADE assessment (including the creation of an evidence profile and summary of findings tables), but only assessed the included EEs risk of bias and publication bias. | The GRADE domains imprecision and inconsistency are not sensitive to vote-counting and the domain indirectness depends vastly on the decision-making context and thus cannot be determined in a general manner. |
| **Assessment of transferability** | We did not assess the transferability of our results to different health systems. | An assessment of transferability would have required us to take a specific health system for reference, but we felt that this would limit the review’s useability to decision-makers and thus decided against it. Instead, we encourage decision-makers to make their own specific judgements using the detailed information on the evidence provided in the text, tables and appendices. |
| **Reporting of amendments** | We did not report important protocol amendments in an updated version of the PROSPERO record. | We did not change the anticipated completion date of our review in PROSPERO in time and were consequently unable to amend the PROSPERO record after that date. Thus, we report all protocol amendments here. |

### Appendix 2 – Search strategies

All databases were searched from their inception to present date. No limits regarding publication date, language, or study design were applied for any database. For screening purposes, full texts published in another language than English or German were translated using DeepL SE (DeepL SE, Cologne, Germany) or Google Translate (Google Inc., Mountain View (CA), USA).

The search strategies do not contain the MeSH/Emtree term ‘Preoperative Exercise’ as it was established on January 1, 2021 (after our initial searches in May 2020).

The search strategies’ recall after the initial search and selection process was greater 90% in all bibliographic databases (92.3% (24/26) for PubMed, 96.4% (27/28) for Embase, and 100% (3/3) for the Centre for Reviews and Dissemination Database), thus we did not revise any search strategies.

1. PubMed via Pubmed (https://pubmed.ncbi.nlm.nih.gov/)

(CBT[tiab] OR Cognitive Behavioral Therapy[tiab] OR Cognitive Behavioural Therapy[tiab] OR Exercise[tiab] OR Fitness[tiab] OR Muscle training[tiab] OR Occupational therap*[tiab] OR Physical activity[tiab] OR Physical training[tiab] OR Physical conditioning[tiab] OR Physical therap*[tiab] OR Physiotherap*[tiab] OR Preconditioning[tiab] OR Prehab*[tiab] OR Rehabilitation[tiab] OR Resistance training[tiab] OR Strength training[tiab] OR Therapeutic activity[tiab] OR “Exercise"[MeSH] OR “Rehabilitation”[MeSH] OR “Physical Therapy Modalities”[MeSH] OR “Cognitive Behavioral Therapy”[MeSh])

AND

(Pre-admission[tiab] OR Preadmission[tiab] OR Pre-hab*[tiab] OR Prehab*[tiab] OR Pre-op*[tiab] OR Preop*[tiab] OR Pre-hosp*[tiab] OR prehosp*[tiab] OR pre-surg*[tiab] OR presurg*[tiab] OR ((Before[tiab] OR prior to[tiab]) AND (surg*[tiab] OR Surgical Procedures, Operative[MeSH])) OR "Preoperative Care"[MeSH])

AND

(cost[tiab] OR costs[tiab] OR economic*[tiab] OR budget*[tiab] OR “Costs and cost analysis”[MeSH] OR "Exercise/economics"[Mesh] OR "Rehabilitation/economics"[Mesh])

1,214 results on 31/08/2021

2. Embase via OVID (https://ovidsp.ovid.com/)

((cbt or (cognitive adj behavioural adj therapy) or (cognitive adj behavioral adj therapy) or exercise or fitness or (muscle adj training) or (occupational adj therap*) or (physical adj activity) or (physical adj training) or (physical adj conditioning) or (physical adj therap*) or physiotherap* or preconditioning or prehab* or rehabilitation or (resistance adj training) or (strength adj training) or (therapeutic adj activity)).ab,kw,ti. or (exp exercise/ or exp rehabilitation/ or exp physiotherapy/ or exp cognitive behavioral therapy/))

and

((preadmission or pre-admission or prehab* or pre-hab* or preop* or pre-op* or pre-hosp* or prehosp* or pre-surg* or presurg*).ab,kw,ti. or exp preoperative period/ or ((before adj5 surg* or (prior adj to) adj5 surg*)). ab,kw,ti.)

and

((cost or costs or economic* or budget*).ab,kw,ti. or exp economic aspect/)

3,451 results on 31/08/2021

3. Centre for Reviews and Dissemination Database (https://www.crd.york.ac.uk/CRDWeb/)

(CBT OR Cognitive Behavioral Therapy OR Cognitive Behavioural Therapy OR Exercise OR Fitness OR Muscle training OR Occupational therap* OR Physical activity OR Physical training OR Physical conditioning OR Physical therap* OR Physiotherap* OR Preconditioning OR Prehab* OR Rehabilitation OR Resistance training OR Strength training OR Therapeutic activity)

AND

(Pre-admission OR Preadmission OR Pre-hab* OR Prehab* OR Pre-op* OR Preop* OR Pre-hosp* OR Prehosp* OR Pre-surg* OR Presurg* OR Before surg* OR prior to surg*)

192 results on 31/08/2021

4. ClinicalTrials.gov (https://clinicaltrials.gov/)

In “other terms”: (Exercise OR training OR conditioning OR physical activity OR ((Physical OR Occupational) AND therapy) OR Prehabilitation OR Rehabilitation) AND (Prehabilitation OR Preoperative OR Preoperatively OR “Before surgery” OR “prior to surgery”) AND cost

578 results on 31/08/2021; 10 results from the search on 04/05/2020 (462 results) could not be retrieved again. All of these records except NCT02294955 had been changed after 04/05/2020; as they still exist, however, the total number is 588.

5. WHO ICTRP (https://trialsearch.who.int/)

1. In Title (no synonyms): “Prehabilitation”

244 results on 31/08/2021

2. In Intervention (no synonyms): “Prehabilitation OR (preoperative AND (exercise OR occupational therapy OR physical activity OR conditioning OR physical therapy OR physiotherapy OR rehabilitation OR therapeutic activity)) OR preconditioning”

629 results on 31/08/2021

6. OATD (https://oatd.org/)

(cbt OR (cognitive AND behavioural AND therapy) OR (cognitive AND behavioral AND therapy) OR exercise OR fitness OR (muscle AND training) OR (occupational AND therap*) OR (physical AND activity) OR (physical AND training) OR (physical AND conditioning) OR (physical AND therap*) OR physiotherap* OR preconditioning OR prehab* OR rehabilitation OR (resistance AND training) OR (strength AND training) OR (therapeutic AND activity))

AND

(preadmission OR prehab* OR preop* OR prehosp* OR presurg* OR (before AND surg*) OR (prior AND to AND surg*))

AND

(cost OR costs OR economic* OR budget*)

85 results on 11/05/2020, 20 results for “last two years” on 30/10/2021

7. DART Europe (https://www.dart-europe.org/)

results on 12/05/2020 (results on 30/10/2021 for the years 2020 and 2021)

prehabilitation = 3 (6); preoperative exercise = 34 (7); preoperative physiotherapy = 6 (2); preoperative fitness = 4 (1); preoperative occupational therapy = 0 (0); preoperative physical activity = 13 (11); preoperative physical training = 8 (1); physical training prior to surgery = 2 (1); preoperative rehabilitation = 25 (7); preoperative resistance training = 4 (0); preoperative strength training = 6 (0); preoperative muscle training = 7 (0); preoperative physical conditioning = 0 (0); preoperative therapeutic activity = 2 (1)

### Appendix 3 – Data items

| **Data item** | **Description** |
| --- | --- |
| **General study characteristics** | |
| Publication details | First author's/principal investigator's last name |
|  | Publication year |
|  | Further references |
|  | Registration number |
|  | Publication type (full journal publication, thesis, conference abstract, protocol article, registration record) |
|  | Study status (according to registration record as per 16 Jan 2023 (ongoing studies only), e.g., recruiting) |
| Funding and conflict of interest | Funding category (non-profit, for-profit, mixed, no funding, not reported) |
|  | Name of funder (sponsor for registration record) |
|  | Conflict of interest |
| Location | City, country |
|  | Continent (derived from country) |
|  | Multicenter (yes, no) |
| Enrolment period | Begin enrolment (for ongoing studies: based on registration record) |
|  | End enrolment (for ongoing studies: estimated end of study according to registration record) |
| Target population | Underlying disease(s) |
|  | Type(s) of surgery |
|  | Inclusion criteria |
|  | Exclusion criteria |
|  | Inclusion criteria relating to high perioperative risk (yes, no) |
| Intervention | Setting (home, outpatient – hospital, outpatient – community, inpatient; referring to physiotherapy element) |
|  | Programme description |
|  | Modality categories by Gurlit et al. 2019 (4); derived from programme description   \| **Category** \| **Subcategory** \| \| --- \| --- \| \| Exercise \| Endurance training \| \|  \| Resistance training \| \|  \| Inspiratory muscle training \| \|  \| Proprioceptive and balance training \| \|  \| Stretching and flexibility \| \|  \| Disease-specific training* (e.g., pelvic floor exercises) \| \| Promotion of physical activity* \| \| \| Nutrition \| Counselling \| \|  \| Supplements \| \| Psychosocial \| Anxiety reduction \| \|  \| Stress management \| \|  \| Other* (e.g., reduction of depression levels, psychological support) \| \| Risk factor reduction \| Cessation of smoking \| \|  \| Cessation of heavy alcohol consumption \| \| Other interventions \| General and specific counselling/education \| \|  \| Advance directives/power of attorney \| \|  \| Stabilizing severe diseases \| \|  \| Drug evaluation \| \|  \| Planning of care** \| \|  \| Other* (e.g., social/financial support, assistive devices) \| \| *(sub-)category added, **Subcategory merged \| \| |
|  | Multi-modal program (yes, no) |
|  | Duration overall in days or weeks |
|  | Frequency (refers to physical therapy element; refers to supervised sessions if there were supervised and unsupervised sessions) |
|  | Duration session in minutes (refers to physical therapy element) |
|  | Health care staff involved |
|  | Evidence-based programme (yes, no; as judged by an experienced physiotherapist; completed EEs only) |
|  | Intensity (high, moderate, low; as judged by an experienced physiotherapist; completed EEs only) |
| Control | Description of usual care (if reported) |
| **Methods (completed EEs and ongoing studies published as protocols only)** | |
| Methods – general  (also for ongoing studies published as registration records) | Type of economic evaluation (EE) (full, partial) |
|  | Type of analysis (cost-utility-analysis, cost-effectiveness-analysis, cost-benefit-analysis, cost–consequence-analysis, cost-minimisation-analysis) |
|  | Design (trial-based, model-based) |
|  | If trial-based: Study type (randomised clinical trial, non-randomised study of interventions) |
|  | If model-based: Model type (e.g., decision tree) |
| Methods – clinical effectiveness | The type of EE determined the clinical effectiveness outcomes to be extracted:   1. Cost-utility analyses (CUAs): quality adjusted life years (QALYs) 2. Cost-effectiveness analyses (CEAs): the specified effectiveness outcome, e.g., health-related quality of life (HrQoL) 3. Cost-benefit analyses (CBA): costs 4. Cost-minimization analyses (CMAs): none 5. Cost-consequence analyses (CCAs): only for outcomes that we considered important to patients: 6. HrQoL 7. Morbidity, i.e., post-operative complications 8. Mortality 9. Patient-reported-outcome measures (PROMs) other than generic HrQoL 10. Physical function   We did not consider length of stay (LOS) in the hospital, intensive care unit or rehabilitation facility, discharge location, ambulation status, readmission, satisfaction surveys or patient-reported experience measures (PREMs), or measurements of function that were not reported alongside a PROM. |
|  | Measurement/definition |
|  | Valuation of effects, e.g., health state valuations from a specified general population |
|  | Source of effects, e.g., medical charts |
|  | Time horizon effects (months) |
|  | Discounting effects (yes, no) |
| Methods – costs | Cost perspective (payer, provider, patient, societal, mix of perspectives; according to Eisenberg 1989. DOI: 10.1001/jama.262.20.2879). |
|  | Type of costs considered (direct, indirect; according to Eisenberg 1989. DOI: 10.1001/jama.262.20.2879). |
|  | Approach to calculate costs (top-down, bottom-up; according to Schoeffski and Graf von der Schulenburg 2012, editors. Gesundheitsökonomische Evaluationen. Berlin, Heidelberg: Springer; 2012. p. 43-70). |
|  | Measurement resource use, e.g., patient diary, hospital billing system |
|  | Valuation resource use, e.g., costs, payments |
|  | Data source resource use, e.g., insurance claims |
|  | Time horizon costs (months) |
|  | Reference year and currency |
|  | Discounting costs (yes, no) |
|  | Inflation (yes, no) |
| Methods - analysis | Statistical analysis (description copied verbatim; only parts referring to the EE; only full EEs) |
|  | Model assumptions (only model-based EE) |
|  | Methods for dealing with uncertainty (only full EEs) |
|  | Handling of missing data (only full EEs) |
|  | Willingness-to-pay threshold (sum and currency; only full EEs) |
| **Results (completed EEs only)** | |
| Study flow | Total number of included patients (also for ongoing studies using the planned sample size) |
|  | Number of included patients in the intervention group (at randomisation/allocation) |
|  | Number of included patients in the control group (at randomisation/allocation) |
|  | Total number of analysed patients |
|  | Number of analysed patients in the intervention group |
|  | Number of analysed patients in the control group |
|  | Reason for exclusion from analysis |
| Study population | Age (mean or median) |
|  | Proportion of female patients |
|  | Description of patient’s comorbidities |
| Adherence | Proportion of actually performed sessions of all scheduled sessions |
|  | Proportion of participants who achieved a sufficient predefined adherence rate, e.g., attendance of at least 80% of sessions |
| Safety | Information on the safety and/or feasibility of the prehabilitation program |
| Clinical effectiveness results  (not for cost-minimization analyses) | QALYs/HrQoL results of intervention group |
|  | QALYs/HrQoL results of control group |
|  | QALYs/HrQoL difference between groups |
|  | Morbidity results of intervention group |
|  | Morbidity results of control group |
|  | Morbidity difference between groups |
|  | Mortality results of intervention group |
|  | Mortality results of control group |
|  | Mortality difference between groups |
|  | PROMs results of intervention group |
|  | PROMs results of control group |
|  | PROMs difference between groups |
|  | Physical function results of intervention group |
|  | Physical function results of control group |
|  | Physical function difference between groups |
| Cost results | Currency and year of costs (copied from methods, assumed where missing) |
|  | Prehabilitation programme costs per patient (only IG) |
|  | Prehabilitation programme costs per patient in EUR 2020 (only IG) calculated using the CCEMG - EPPI-Centre Cost Converter, version 1.6 (23):  In a first step, costs were converted to 2020 EUR using the ‘Implied Inflation Factor’ as the source dataset for purchasing power parity values and Germany as a reference country (as it was mandatory to choose a specific country and there was no option to select the Eurozone as a whole). In the second step, 2020 EUR Germany values were converted to Europe-wide EUR values using EUROSTAT data for comparative price levels (Available from:  <https://ec.europa.eu/eurostat/databrowser/view/tec00120/default/table?lang=en>).  When the year of the original currency was unclear, we chose the study’s last year of recruitment or, if not reported, the year of publication. When more than one year was reported for costs, we converted the costs for all years and reported the mean values. Where costs had already been converted to EUR without naming the reference country, we selected Germany as the country before converting the values. |
|  | Quantities of resource use |
|  | Unit costs (original currency and year) |
|  | Costs of resource use (per resource domain listed) |
|  | Total costs of intervention group |
|  | Total costs of control group |
|  | Difference in total costs between groups |
| Cost-effectiveness results | ICER (only full EEs) |
|  | ICER in EUR 2020 (only full EEs; calculated using the CCEMG - EPPI-Centre Cost Converter (23) (details see above)) |
|  | Cost-effectiveness (cost-effective, unclear; incremental analysis required, unclear; individual decision required, neutral, not cost-effective) |
|  | Study authors' conclusion (only full EEs) |

### Appendix 4 – List of excluded studies

Where more than one exclusion reason applied, we chose the first reason according to the PICO scheme. Studies that seem to meet the inclusion criteria, but which were excluded, are marked as “close misses” and an additional explanation for their exclusion is provided.

| No. | Reference | Reason for exclusion |
| --- | --- | --- |
| 1. | [No author]. "Impact of Pelvic Floor Prehabilitation Using Biofeedback on the Severity of the Low Anterior Resection Syndrome in Patients Undergoing a Total Mesorectal Excision for Rectal Cancer". https://ClinicalTrials. gov/show/NCT03876561; 2019 | Intervention |
| 2. | [No author]. A Trimodal Prehabilitation Study for Patients Undergoing Major Abdominal Surgery. https://ClinicalTrials. gov/show/NCT04047524; 2019 | Outcome |
| 3. | [No author]. An Innovative Tailored Intervention for Improving Children's Postoperative Recovery (WebTIPS). https://ClinicalTrials. gov/show/NCT03730259; 2019 | Intervention |
| 4. | [No author]. Application of remote ischemic preconditioning combined with low central venous pressure in liver surgery.http://www.chictr.org.cn/showproj.aspx?proj=121848. | Intervention |
| 5. | [No author]. Assessing if Cryoneurolysis Improves Prehabilitation and Decreases Pain After Surgery With Less Opioid Use in TKA Patients.https://clinicaltrials.gov/show/NCT03836313. | Intervention |
| 6. | [No author]. Can patient education focused on coping with pain facilitate return to work and everyday life following lumbar spinal fusion surgery? 2014.http://isrctn.com/ISRCTN42281022. | Intervention |
| 7. | [No author]. Cognitive Training for the Prevention of Postoperative Delirium. https://ClinicalTrials. gov/show/NCT02963961; 2017 | Intervention; Close miss: No physiotherapy element (cognitive training only) |
| 8. | [No author]. Cost-effectiveness of biceps tenotomy with or without cuff repair in patients with stage 2-3 Goutallier fatty degenerative cuff lesions. A randomized controlled trial.https://trialregister.nl/trial/4010. | Intervention |
| 9. | [No author]. Does Participation in a Pre-Operative Physical Activity Program Improve Patient Outcomes and Quality of Life? : https://ClinicalTrials. gov/show/NCT03049696; 2017. | Outcome |
| 10. | [No author]. Effect of Remote Ischaemic Preconditioning on Renal Function in Patients Undergoing Living Donor Kidney Transplantation.https://clinicaltrials.gov/show/NCT01289548. | Intervention |
| 11. | [No author]. Effectiveness of Pain Neuroscience Education vs Biomedical Education for Patients Undergoing Surgery for Shoulder Pain. https://ClinicalTrials. gov/show/NCT02960477; 2019 | Intervention |
| 12. | [No author]. Efficacy of Preoperative Prehabilitation With a Home-based Supervised Exercise Program Against an Unsupervised Exercise Program for Frail Elderly Patients Undergoing Major Abdominal Surgery.https://ClinicalTrials.gov/show/NCT04892368.gov/show/NCT04892368; 2021 | Outcome |
| 13. | [No author]. Exercise Prehabilitation in High Risk Cancer Surgery: A Feasibility Study.https://anzctr.org.au/ACTRN12620000073909.aspx. | Control |
| 14. | [No author]. Fast-Track Rehabilitation. https://ClinicalTrials. gov/show/NCT03530059; 2018 | Study type |
| 15. | [No author]. Feasibility of the Hip Instructional Prehabilitation Program for Enhanced Recovery (HIPPER). https://ClinicalTrials. gov/show/NCT02969512; 2019 | Outcome |
| 16. | [No author]. Fit-Joint: Getting Fit for Hip or Knee Replacement. https://ClinicalTrials. gov/show/NCT02885337; 2016 | Outcome; Close miss: No cost outcome listed under outcomes |
| 17. | [No author]. Home-based Prehabilitation for Elderly Patients. https://ClinicalTrials. gov/show/NCT03964363; 2019 | Intervention |
| 18. | [No author]. Home-Initiated Programme to Prepare for Operation (HIPPO) Study. https://ClinicalTrials. gov/show/NCT04271553; 2018 | Intervention |
| 19. | [No author]. Immune Modulation by Enhanced vs Standard Prehabilitation Program Before Major Surgery.https://ClinicalTrials.gov/show/NCT04498208.gov/show/NCT04498208; 2020 | Outcome |
| 20. | [No author]. Implementation of a Cognitive Training Program to Reduce the Risk of Postoperative Cognitive Dysfunction. https://ClinicalTrials. gov/show/NCT03620968; 2018 | Intervention |
| 21. | [No author]. Implementation of Collaborative Self-management Services to Promote Physical Activity. https://ClinicalTrials. gov/show/NCT02976064; 2016 | Outcome; Close miss: Implementation study (no cost outcome listed under outcomes) |
| 22. | [No author]. Improving ACL Reconstruction Outcomes: CBPT. https://ClinicalTrials. gov/show/NCT03243162; 2017 | Intervention; Close miss: Telephone intervention (no in-person meeting); control group also received intervention (not usual care) |
| 23. | [No author]. Incentive Spirometry Prehabilitation Study. https://ClinicalTrials. gov/show/NCT03994848; 2019 | Outcome |
| 24. | [No author]. Increasing Preoperative Cognitive Reserve to Prevent Postoperative Cognitive Dysfunction in Cardiac Surgical Patients.https://ClinicalTrials.gov/show/NCT04493996.gov/show/NCT04493996; 2020 | Intervention |
| 25. | [No author]. Influence of Postoperative Rehabilitation and Pre- and Postoperative Physical Activity in Abdominal Surgery. https://ClinicalTrials. gov/show/NCT02829125; 2015 | Outcome |
| 26. | [No author]. Is There an Alternative to Immediate Home Physical Therapy Following Total Knee Arthroplasty. https://ClinicalTrials. gov/show/NCT04060251; 2019 | Intervention |
| 27. | [No author]. Isometric Preoperative Exercise on Autologous Arteriovenous Fistulas. Randomized Clinical Trial. https://ClinicalTrials. gov/show/NCT03213756; 2017 | Outcome |
| 28. | [No author]. Long Term Effect of Trimodal Prehabilitation Compared to ERAS in Colorectal Cancer Surgery.https://ClinicalTrials.gov/show/NCT04595604.gov/show/NCT04595604; 2020 | Outcome |
| 29. | [No author]. Mobility and Activity Training (MAT) to Optimize Outcomes for Older Adult Abdominal Surgery Patients. https://ClinicalTrials. gov/show/NCT02007876; 2013 | Intervention |
| 30. | [No author]. Move For Surgery - A Novel Preconditioning Program. https://ClinicalTrials. gov/show/NCT03689634; 2018 | Intervention |
| 31. | [No author]. Multimodal Prehabilitation for Surgery in the Elderly: A Randomised, Prospective, Multicenter, Multidisciplinary Trial.https://ClinicalTrials.gov/show/NCT04461301.gov/show/NCT04461301; 2022 | Outcome |
| 32. | [No author]. Optimalisation of the perioperative treatment in kidney donors:&#x0D; Fast track perioperative care program vs standard perioperative care program in hand-assisted laparoscopic donornephrectomy.https://trialregister.nl/trial/1964. | Intervention |
| 33. | [No author]. Osteoarthritis preoperative package for care of orthotics, rehabilitation, topical and oral agent usage and nutrition to improve outcomes at a year.http://isrctn.com/ISRCTN96684272. | Ouctome |
| 34. | [No author]. PAC Time Assessment. https://ClinicalTrials. gov/show/NCT03451604; 2018 | Intervention |
| 35. | [No author]. PAPRIKA - Patients Empowerment for Major Surgery Preparation @Home. https://ClinicalTrials. gov/show/NCT04295668; 2020 | Outcome |
| 36. | [No author]. Pre-surgical Protocol for Frail Elderly People in Order to Reduce Hospitalization Days (APOPM).https://ClinicalTrials.gov/show/NCT04770259.gov/show/NCT04770259; 2021 | Outcome |
| 37. | [No author]. Prehabilitation Versus Enhanced Recovery Program for Elective Colorectal Cancer Surgery. https://ClinicalTrials. gov/show/NCT03758209; 2018 | Outcome |
| 38. | [No author]. Preoperative High Intensity Interval Training: The PHIIT Trial. https://ClinicalTrials. gov/show/NCT02674815; 2016 | Study type |
| 39. | [No author]. Preoperative Vestibular Rehabilitation Effectiveness After Vestibular Schwannoma Surgery. https://ClinicalTrials. gov/show/NCT02275325; 2015 | Outcome |
| 40. | [No author]. PREPARE (PREhabilitation, Physical Activity and ExeRcisE) persons with severe low back pain for an optimal functional outcome after lumbar fusion surgery.http://isrctn.com/ISRCTN17115599. | Intervention |
| 41. | [No author]. Preventive Heart Rehabilitation to Prevent Complications in Patients Undergoing Elective Open Heart Surgery. https://ClinicalTrials. gov/show/NCT02984449; 2017 | Intervention |
| 42. | [No author]. Protective effect of remote ischemic preconditioning combined with sevoflurane posttreatment on myocardial injury during laparoscopic radical resection of colorectal cancer.http://www.chictr.org.cn/showproj.aspx?proj=61150. | Intervention |
| 43. | [No author]. Psychological Preparation for Colorectal Surgery: Impact of Video Education. https://ClinicalTrials. gov/show/NCT01547572; 2010 | Intervention |
| 44. | [No author]. Respiratory Muscle Training Before Surgery in Preventing Lung Complications in Patients With Stage I-IIIB Lung Cancer. https://ClinicalTrials. gov/show/NCT04067830; 2019 | Outcome |
| 45. | [No author]. Telephone Intervention to Increase Patient Preparedness and Satisfaction Trial (TIPPS): A Randomized Control Trial. https://ClinicalTrials. gov/show/NCT03890471; 2019 | Intervention |
| 46. | [No author]. The Effect of Coaching Strategy on Some Patient Outcomes After Total Knee Arthroplasty Surgery.https://ClinicalTrials.gov/show/NCT04683588.gov/show/NCT04683588; 2021 | Intervention |
| 47. | [No author]. The Effect of Preoperative Assessment Clinic on Prognosis and Economic Results of Patients With Coexisting Disease. https://ClinicalTrials. gov/show/NCT03665987; 2018 | Intervention |
| 48. | [No author]. The Impact of a Bariatric Rehabilitation Service on Patient Outcomes. https://ClinicalTrials. gov/show/NCT01264120; 2011 | Intervention |
| 49. | [No author]. The Impact of a Pre-Operative Exercise Program on Fitness Outcomes Following Bariatric Surgery. https://ClinicalTrials. gov/show/NCT02010489; 2014 | Outcome |
| 50. | [No author]. Trial of Prehabilitation in Vulnerable Patients Undergoing Cystectomy for Bladder Cancer. https://ClinicalTrials. gov/show/NCT01840137; 2012 | Study type |
| 51. | [No author]. Using Multimodal Prehabilitation to Improve Outcomes for Frail Patients Undergoing Resection of Colorectal Cancer. https://ClinicalTrials. gov/show/NCT02502760; 2015 | Intervention |
| 52. | Almada P, Archer R. Planning ahead for better outcomes: preparation for joint replacement surgery begins at home!. Orthop Nurs. 2009;28(1):3-8; quiz 9-10 | Intervention |
| 53. | Ambrosino N, Gabbrielli L. Physiotherapy in the perioperative period. Best Pract Res Clin Anaesthesiol. 2010;24(2):283-9 | Study type |
| 54. | Andras L, Thomas L, Dias D. Perioperative surgical home and the total knee arthroplasty patient: Improving care and reducing costs. Regional Anesthesia and Pain Medicine. 2016;41(5) | Intervention |
| 55. | Aragoncillo I, Ligero JM, Hevia C, Morales AL, Amézquita Y, Cervera T, et al. Rationale and design of the PHYSICALFAV trial: a randomized controlled trial to evaluate the effect of preoperative isometric exercise on vascular calibre and maturation of autologous arteriovenous fistulas. Clin Kidney J. 2018;11(6):841-5 | Outcome |
| 56. | Arcêncio L, Souza MD, Bortolin BS, Fernandes AC, Rodrigues AJ, Evora PR. Pre-and postoperative care in cardiothoracic surgery: a physiotherapeutic approach. Rev Bras Cir Cardiovasc. 2008;23(3):400-10 | Study type |
| 57. | Archer K, Ciechanowicz H. The effectiveness of preoperative pulmonary rehabilitation in reducing postoperative pulmonary complications in lung cancer: a systematic review and meta-analysis. Physiotherapy (United Kingdom). 2019;105:e211-e2 | Study type |
| 58. | Argudo N, Rodó-Pin A, Martínez-Llorens J, Marco E, Visa L, Messaggi-Sartor M, et al. Feasibility, tolerability, and effects of exercise-based prehabilitation after neoadjuvant therapy in esophagogastric cancer patients undergoing surgery: an interventional pilot study. Dis Esophagus. 2021;34(4) | Control |
| 59. | Avancini A, Cavallo A, Trestini I, Tregnago D, Belluomini L, Crisafulli E, et al. Exercise prehabilitation in lung cancer: Getting stronger to recover faster. Eur J Surg Oncol. 2021;47(8):1847-55 | Study type |
| 60. | Baillot A, Vallée CA, Mampuya WM, Dionne IJ, Comeau E, Méziat-Burdin A, et al. Effects of a Pre-surgery Supervised Exercise Training 1 Year After Bariatric Surgery: a Randomized Controlled Study. Obes Surg. 2018;28(4):955-62 | Outcome |
| 61. | Baimas-George M, Watson M, Elhage S, Parala-Metz A, Vrochides D, Davis BR. Prehabilitation in Frail Surgical Patients: A Systematic Review. World J Surg. 2020;44(11):3668-78 | Study type |
| 62. | Barbay K. Research evidence for the use of preoperative exercise in patients preparing for total hip or total knee arthroplasty. Orthopaedic Nursing\. 2009;28(3):127-33\ | Study type |
| 63. | Barberan-Garcia A, Cano I, Bongers BC, Seyfried S, Ganslandt T, Herrle F, et al. Digital Support to Multimodal Community-Based Prehabilitation: Looking for Optimization of Health Value Generation. Front Oncol. 2021;11:662013 | Study type |
| 64. | Bennell KL, Spiers L, Takla A, O'Donnell J, Kasza J, Hunter DJ, et al. Efficacy of adding a physiotherapy rehabilitation programme to arthroscopic management of femoroacetabular impingement syndrome: A randomised controlled trial (FAIR). BMJ Open. 2017;7(6) | Intervention |
| 65. | Benzo R, Wigle D, Novotny P, Wetzstein M, Nichols F, Shen RK, et al. Preoperative pulmonary rehabilitation before lung cancer resection: results from two randomized studies. Lung Cancer. 2011;74(3):441-5 | Outcome |
| 66. | Berkel AEM, Bongers BC, van Kamp MS, Kotte H, Weltevreden P, de Jongh FHC, et al. The effects of prehabilitation versus usual care to reduce postoperative complications in high-risk patients with colorectal cancer or dysplasia scheduled for elective colorectal resection: study protocol of a randomized controlled trial. BMC Gastroenterol. 2018;18(1):29. | Post-hoc exclusion: No cost outcome in study publication(s) |
| 67. | Blackwell JEM, Doleman B, Boereboom CL, Morton A, Williams S, Atherton P, et al. High-intensity interval training produces a significant improvement in fitness in less than 31 days before surgery for urological cancer: a randomised control trial. Prostate Cancer Prostatic Dis. 2020 | Outcome |
| 68. | Blasco JM, Igual-Camacho C, Roig-Casasús S. In-home versus hospital preoperative balance and proprioceptive training in patients undergoing TKR; rationale, design, and method of a randomized controlled trial. BMC Musculoskelet Disord. 2017;18(1):518. | Post-hoc exclusion: No cost outcome in study publication(s) |
| 69. | Boden I, Browning L, Skinner EH, Reeve J, El-Ansary D, Robertson IK, et al. The LIPPSMAck POP (Lung Infection Prevention Post Surgery - Major Abdominal - with Pre-Operative Physiotherapy) trial: study protocol for a multi-centre randomised controlled trial. Trials. 2015;16:573 | Intervention; Close miss: Protocol for Boden 2018; Only one session to prepare patients to perform the exercise post-operatively ("...will then receive an additional single education and training session of approximately 30 min with a physiotherapist"). Hence no preoperative training as such. |
| 70. | Boden I, Robertson IK, Neil A, Reeve J, Palmer AJ, Skinner EH, et al. Preoperative physiotherapy is cost-effective for preventing pulmonary complications after major abdominal surgery: a health economic analysis of a multicentre randomised trial. J Physiother. 2020;66(3):180-7 | Intervention; Close miss: Only one session to prepare patients to perform the exercise post-operatively ("...will then receive an additional single education and training session of approximately 30 min with a physiotherapist"). Hence no preoperative training as such; close miss |
| 71. | Boden I, Skinner EH, Browning L, Reeve J, Anderson L, Hill C, et al. Preoperative physiotherapy for the prevention of respiratory complications after upper abdominal surgery: pragmatic, double blinded, multicentre randomised controlled trial. Bmj. 2018;360:j5916 | Intervention; Close miss: See above (Boden 2015). Also no cost outcome (in this publication). "Detailed modelling of specific costs and health economics supporting this clinical efficacy report will be published later." |
| 72. | Bogue E, Twiggs J, Liu D. Prehabilitation using a novel, mobile application reduces length of stay in patients undergoing primary total knee arthroplasty. Journal of Orthopaedic Research. 2017;35 | Outcome |
| 73. | Bolshinsky V, Li MH, Ismail H, Burbury K, Riedel B, Heriot A. Multimodal Prehabilitation Programs as a Bundle of Care in Gastrointestinal Cancer Surgery: A Systematic Review. Dis Colon Rectum. 2018;61(1):124-38 | Study type |
| 74. | Bond-Smith G, Belgaumkar AP, Davidson BR, Gurusamy KS. Enhanced recovery protocols for major upper gastrointestinal, liver and pancreatic surgery. Cochrane Database Syst Rev. 2016;2:Cd011382 | Study type |
| 75. | Bonner RJ, Wallace T, Jones AD, Julian Scott D, Richards SH. The Content of Pre-habilitative Interventions for Patients Undergoing Repair of Abdominal Aortic Aneurysms and Their Effect on Post-Operative Outcomes: A Systematic Review. Eur J Vasc Endovasc Surg. 2021;61(5):756-65 | Study type |
| 76. | Bousquet-Dion G, Awasthi R, Loiselle S, Minnella EM, Agnihotram RV, Bergdahl A, et al. Evaluation of supervised multimodal prehabilitation programme in cancer patients undergoing colorectal resection: a randomized control trial. Acta Oncol. 2018;57(6):849-59 | Outcome |
| 77. | Bowe F, Paul I, Ferguson J, Dunning J, Harrison SL. High intensity inspiratory muscle training (HI-IMT) in individuals referred for lung resection surgery. Physiotherapy (United Kingdom). 2020;107 (Supplement 1):e33-e4 | Control |
| 78. | BPCI Initiative Cuts Readmissions, Costs for Patients Having Cardiac Valve Surgery. Hosp Case Manag. 2016;24(9):127-29 | Study type |
| 79. | Bradley A, Marshall A, Stonehewer L, Reaper L, Parker K, Bevan-Smith E, et al. Pulmonary rehabilitation programme for patients undergoing curative lung cancer surgery. Eur J Cardiothorac Surg. 2013;44(4):e266-71 | Intervention; Close miss: Pre- and post-operative intervention ("Between 4 and 6 weeks post-hospital discharge, the intervention group rejoined the rehabilitation programme twice weekly for up to 3 months and was then offered maintenance sessions once a week.") |
| 80. | Branea H, Streian C, Sofi AM, Genes E. Exercise training before surgical revascularisation-influence on short term outcomes. JK Practitioner. 2015;20(1-2):21-4 | Intervention |
| 81. | Brooks D, Crowe J, Kelsey CJ, Lacy JB, Parsons J, Solway S. A clinical practice guideline on peri-operative cardiorespiratory physical therapy. Physiotherapy Canada\. 2001;53(1):9-25\ | Full text not available |
| 82. | Burtonwood J, Ing H, Mundell C. So what happens next ? integrated prehabillitation in primary care. Supportive Care in Cancer. 2021;29 (SUPPL 1):S93 | Outcome |
| 83. | Butler GS, Hurley CA, Buchanan KL, Smith-VanHorne J. Prehospital education: effectiveness with total hip replacement surgery patients. Patient Educ Couns. 1996;29(2):189-97 | Intervention |
| 84. | Carli F, Feldman LS. From preoperative risk assessment and prediction to risk attenuation: a case for prehabilitation. British Journal of Anaesthesia. 2019;122(1):11-3 | Study type |
| 85. | Carli F, Gillis C, Scheede-Bergdahl C. Promoting a culture of prehabilitation for the surgical cancer patient. Acta Oncol. 2017;56(2):128-33 | Study type |
| 86. | Carli F, Minnella EM. Preoperative functional assessment and optimization in surgical patient: changing the paradigm. Minerva Anestesiol. 2017;83(2):214-8 | Study type |
| 87. | Carli F, Silver JK, Feldman LS, McKee A, Gilman S, Gillis C, et al. Surgical Prehabilitation in Patients with Cancer: State-of-the-Science and Recommendations for Future Research from a Panel of Subject Matter Experts. Phys Med Rehabil Clin N Am. 2017;28(1):49-64 | Study type |
| 88. | Carmine Riccioy D, Gennaro Ismeno D, Ssa Maria Teresa Peyer D, Maria Teresa Peyer MT. The physioterapic treatment pre and post operatory in cardiac surgery: Better outcome and complicance reduction or only psycologic progress? European Journal of Preventive Cardiology. 2013;20(1):S100. | Population |
| 89. | Casner P, Hilty SA, Smetana GW, Cohn SL, Lawrence VA. Update in perioperative medicine [5] (multiple letters). Annals of Internal Medicine. 2004;141(6):486-7 | Study type |
| 90. | Cassini D, Clementi S, Colletti G, Cortellazzi P, Baldazzi G. ERAS prehabilitation in “low budget” era…“where there's a will, there's a way”. Clinical Nutrition ESPEN. 2019;31:140-1 | Outcome |
| 91. | Cave T, Neck C. Can a physical activity intervention for individuals with an ovarian cancer diagnosis improve outcomes? Physiotherapy (United Kingdom). 2019;105:e150-e1. | Outcome |
| 92. | Chmelo J, Phillips AW, Greystoke A, Charman SJ, Avery L, Hallsworth K, et al. A feasibility study to investigate the utility of a home-based exercise intervention during and after neo-adjuvant chemotherapy for oesophago-gastric cancer-the ChemoFit study protocol. Pilot Feasibility Stud. 2020;6:50 | Outcome |
| 93. | Chughtai M, Shah NV, Sultan AA, Solow M, Tiberi JV, Mehran N, et al. The role of prehabilitation with a telerehabilitation system prior to total knee arthroplasty. Ann Transl Med. 2019;7(4):68 | Intervention |
| 94. | Colquhoun AD, Zuelzer W, Butterworth JF. Improving the management of hip fractures in the elderly: A role for the perioperative surgical home? Anesthesiology. 2014;121(6):1144-6. | Study type |
| 95. | Contreras Delgado AN, Flores Echeverria MI, Romero Bielma E, Banos Mejia O, Galicia Amor S. Pulmonary rehabilitation in lung and pleural cancer patients: Preliminary report. Journal of Cardiopulmonary Rehabilitation and Prevention. 2016;36 (4):297 | Outcome |
| 96. | C̈orovic̈ A, Griffiths R. Pre-habilitation (ii): Time for a patient-doctor contract? Anaesthesia. 2014;69(5):407-10. | Study type |
| 97. | Coudeyre E, Jardin C, Givron P, Ribinik P, Revel M, Rannou F. Could preoperative rehabilitation modify postoperative outcomes after total hip and knee arthroplasty? Elaboration of French clinical practice guidelines. Ann Readapt Med Phys. 2007;50(3):189-97 | Study type |
| 98. | Crowe J, Henderson J. Pre-arthroplasty rehabilitation is effective in reducing hospital stay. Can J Occup Ther. 2003;70(2):88-96 | Outcome |
| 99. | Cui HW, Turney BW, Griffiths J. The Preoperative Assessment and Optimization of Patients Undergoing Major Urological Surgery. Current Urology Reports. 2017;18(7) | Study type |
| 100. | Dand A, Taylor L, Hamdan Y, Smethurst G, Dean G, Whiteside K. Implementation of incremental shuttle walk testing for lung cancer patients. Lung Cancer. 2020;139:S75 | Outcome |
| 101. | Dao TK, Youssef NA, Armsworth M, Wear E, Papathopoulos KN, Gopaldas R. Randomized controlled trial of brief cognitive behavioral intervention for depression and anxiety symptoms preoperatively in patients undergoing coronary artery bypass graft surgery. Journal of Thoracic and Cardiovascular Surgery. 2011;142(3):e109-e15 | Intervention |
| 102. | das Nair R, Anderson P, Clarke S, Leighton P, Lincoln NB, Mhizha-Murira JR, et al. Home-administered pre-surgical psychological intervention for knee osteoarthritis (HAPPiKNEES): study protocol for a randomised controlled trial. Trials. 2016;17:54 | Intervention |
| 103. | Dasari M, Ayo D, McMacken M, Ogedegbe O, Parikh M. Does a preoperative medically supervised weight loss program improve bariatric surgery outcomes: A pilot randomized study. Surgical Endoscopy and Other Interventional Techniques. 2011;25:S255 | Outcome |
| 104. | Delgado Floody P, Jerez Mayorga D, Caamaño Navarrete F, Concha Díaz M, Ovalle Elgueta H, Osorio Poblete A. [EFFECTIVENESS OF COMPREHENSIVE TREATMENT ON THE PREOPERATIVE CONDITIONS OF OBESE WOMEN CANDIDATES FOR BARIATRIC SURGERY]. Nutr Hosp. 2015;32(6):2570-5 | Outcome |
| 105. | Delgado-López PD, Rodríguez-Salazar A, Castilla-Díez JM. "Prehabilitation" in degenerative spine surgery: A literature review. Neurocirugia (Astur). 2019;30(3):124-32 | Study type |
| 106. | Devereaux B, Skinner C, Myhill R, Hopkins G. Intensive medical weight loss program achieves significant rapid pre-operative weight loss in the obese population. Journal of Gastroenterology and Hepatology (Australia). 2014;29:145 | Outcome |
| 107. | Dowsey MM, Castle DJ, Knowles SR, Monshat K, Salzberg MR, Choong PF. The effect of mindfulness training prior to total joint arthroplasty on post-operative pain and physical function: study protocol for a randomised controlled trial. Trials. 2014;15:208 | Intervention |
| 108. | Duchalais E. Impact of pelvic floor prehabilitation using biofeedback therapy on the severity of low anterior resection syndrome following total mesorectal excision: CONTICARE randomized controlled trial. Colorectal Disease. 2019;21 (Supplement 3):131 | Intervention |
| 109. | Dunne D, Jones R, Lythgoe D, Malik H, Poston GJ, Jack S, et al. Prehabilitation before liver surgery. European Journal of Surgical Oncology. 2014;40(11):S52 | Outcome |
| 110. | Emanuel O, Greenslade K, Lechner M, Eynon-Lewis N. Mindfulness for patients undergoing surgery: a cost-effective and potentially underrated tool for improving outcomes. Br J Anaesth. 2021;127(1):e22-e4 | Intervention |
| 111. | Fagevik Olsen M, Anzen H. Effects of training interventions prior to thoracic or abdominal surgery: a systematic review. Physical Therapy Reviews\. 2012;17(2):124-31\ | Study type |
| 112. | Fenton C, Tan AR, Abaraogu UO, McCaslin JE. Prehabilitation exercise therapy before elective abdominal aortic aneurysm repair. Cochrane Database Syst Rev. 2021;7:Cd013662 | Study type |
| 113. | Fleege C, Arabmotlagh M, Almajali A, Rauschmann M. Pre- and postoperative fast-track treatment concepts in spinal surgery : patient information and patient cooperation. Der Orthopäde. 2014;43(12):1062-4, 6-9 | Outcome |
| 114. | Furze G, Dumville JC, Miles JN, Irvine K, Thompson DR, Lewin RJ. "Prehabilitation" prior to CABG surgery improves physical functioning and depression. Int J Cardiol. 2009;132(1):51-8 | Intervention; Close miss: No physiotherapy element (booklet & CD, telephone calls); Control group also received intervention (education & counselling) |
| 115. | Galbraith AS, McGloughlin E, Cashman J. Enhanced recovery protocols in total joint arthroplasty: a review of the literature and their implementation. Ir J Med Sci. 2018;187(1):97-109 | Study type |
| 116. | Galbraith AS, McGloughlin EK, Cashman J. Enhanced recovery protocols in total joint arthroplasty, a review of the evidence and implementation. Irish Journal of Medical Science. 2017;186(3):S157-S8 | Study type |
| 117. | Gao S, Barello S, Chen L, Chen C, Che G, Cai K, et al. Clinical guidelines on perioperative management strategies for enhanced recovery after lung surgery. Translational Lung Cancer Research. 2019;8(6):1174-87 | Study type |
| 118. | Garson L, Schwarzkopf R, Vakharia S, Alexander B, Stead S, Cannesson M, et al. Implementation of a total joint replacement-focused perioperative surgical home: a management case report. Anesth Analg. 2014;118(5):1081-9 | Outcome |
| 119. | Gill SD, McBurney H. Does exercise reduce pain and improve physical function before hip or knee replacement surgery? A systematic review and meta-analysis of randomized controlled trials. Archives of Physical Medicine and Rehabilitation\. 2013;94(1):164-76\ | Study type |
| 120. | Gill TM, Baker DI, Gottschalk M, Gahbauer EA, Charpentier PA, De Regt PT, et al. A prehabilitation program for physically frail community-living older persons. Archives of Physical Medicine and Rehabilitation. 2003;84(3 SUPPL. 1):394-404 | Outcome |
| 121. | Gillis C, Buhler K, Bresee L, Carli F, Gramlich L, Culos-Reed N, et al. Effects of Nutritional Prehabilitation, With and Without Exercise, on Outcomes of Patients Who Undergo Colorectal Surgery: A Systematic Review and Meta-analysis. Gastroenterology. 2018;155(2):391-410 e4 | Study type |
| 122. | Gilmore SJ, McClelland JA, Davidson M. Physiotherapeutic interventions before and after surgery for degenerative lumbar conditions: a systematic review. Physiotherapy\. 2014:epub\ | Study type |
| 123. | Girard N. Evidence appraisal of Boden I, Skinner EH, Browning L, et al. Preoperative physiotherapy for the prevention of respiratory complications after upper abdominal surgery: pragmatic, double blinded, multicentre randomised controlled trial. BMJ. 2018;360:j5916 | Study type |
| 124. | Glauser G, Ali ZS, Gardiner D, Ramayya AG, Pessoa R, Grady MS, et al. Assessing the utility of an IoS application in the perioperative care of spine surgery patients: the NeuroPath Pilot study. Mhealth. 2019;5:40 | Intervention |
| 125. | Gometz A, Maislen D, Youtz C, Kary E, Gometz EL, Sobotka S, et al. The Effectiveness of Prehabilitation (Prehab) in Both Functional and Economic Outcomes Following Spinal Surgery: A Systematic Review. Cureus. 2018;10(5):e2675 | Study type |
| 126. | Greaves J, Dawkins D, McNaught J, Clark L. Pre operative education-improving patient experience for lower limb arthroplasty patients. Physiotherapy (United Kingdom). 2017;103:e42-e3 | Outcome |
| 127. | Grocott MPW, Ludbrook GL. Economic evaluation of prehabilitation: a true return on investment? Br J Anaesth. 2019;123(6):710-2. | Study type |
| 128. | Gunnarsson AK, Lönn K, Gunningberg L. Does nutritional intervention for patients with hip fractures reduce postoperative complications and improve rehabilitation? J Clin Nurs. 2009;18(9):1325-33. | Intervention |
| 129. | Halliday L, Doganay E, Lada H, Wynter-Blyth V, Hanna G, Moorthy K. Prehabilitation in oesophago-gastric cancer: The impact on post-operative outcomes. British Journal of Surgery. 2019;106 (Supplement 5):79. | Post-hoc exclusion: No cost outcome in study publication(s) |
| 130. | Hartmann CEA, Ko LWL, Jones GJ, Nathwani D. Preoperative patient education as tool to improving post-operative patient recovery. International Journal of Surgery. 2013;11(8):668 | Intervention |
| 131. | Hartog J, Blokzijl F, Dijkstra S, DeJongste MJL, Reneman MF, Dieperink W, et al. Heart Rehabilitation in patients awaiting Open heart surgery targeting to prevent Complications and to improve Quality of life (Heart-ROCQ): study protocol for a prospective, randomised, open, blinded endpoint (PROBE) trial. BMJ Open. 2019;9(9):e031738 | Intervention |
| 132. | Hass S, Jaekel C, Nesbitt B. Nursing strategies to reduce length of stay for persons undergoing total knee replacement: integrative review of key variables. J Nurs Care Qual. 2015;30(3):283-8 | Study type |
| 133. | Hayes. Preoperative Behavioural Intervention for Risky Drinkers before elective orthopaedic Surgery (Preop-BIRDS). Impact of preoperative supervised weight loss programs on bariatric surgery outcomes. Lansdale, PA: HAYES Inc\; 2017. | Study type |
| 134. | Heiman Ullmark J, Bock D, Fagevik Olsén M, Olofsson Bagge R, Haglind E. PhysSurg-B-PHYSical activity in relation to SURGical operations. European Journal of Cancer. 2018;92:S77 | Intervention |
| 135. | Hernon J. SupPoRtive Exercise Programmes for Accelerating REcovery after major ABdominal Cancer surgery (PREPARE-ABC). Colorectal Disease. 2016;18:126 | Intervention |
| 136. | Hernon J. SupPoRtive Exercise Programmes for Accelerating REcovery after major ABdominal Cancer surgery trial (PREPARE-ABC): study protocol for a multi-centre randomised controlled trial. Colorectal Dis.2021 | Intervention; Close miss: Pre- and post-operative intervention (post-operative booster session) |
| 137. | Hile E, Hoffman L, Postier R, Ding K, Yang J, Li M. A pilot RCT of sarcopenia-focused prehabilitation in pancreas cancer. Journal of Clinical Oncology. 2017;35(15) | Outcome |
| 138. | Hirschmüller A, Schoch W, Baur H, Wondrasch B, Konstantinidis L, Südkamp NP, et al. Rehabilitation before regenerative cartilage knee surgery: a new prehabilitation guideline based on the best available evidence. Arch Orthop Trauma Surg. 2019;139(2):217-30 | Study type |
| 139. | Hodgkinson B, Evans D, O'Neill S. Knowledge retention from pre-operative patient information. Title to be Checked \. 2000:1-47\ | Study type |
| 140. | Hollywood A, Ogden J, Pring C. The impact of a bariatric rehabilitation service on weight loss and psychological adjustment--study protocol. BMC Public Health. 2012;12:275 | Intervention |
| 141. | Hoogeboom TJ, Oosting E, Vriezekolk JE, Veenhof C, Siemonsma PC, de Bie RA, et al. Therapeutic validity and effectiveness of preoperative exercise on functional recovery after joint replacement: a systematic review and meta-analysis. Plos One\. 2012;7(5) | Study type |
| 142. | Hoppeler H. Importance of preoperative physical activity. Obesity Surgery. 2011;21(8):1041-2 | Study type |
| 143. | Horstmann T, Mayer F, Niess A, Röcker K, Dickhuth HH. New aspects in rehabilitation of osteoarthritic and endoprosthesis patients. Medizinische Welt. 2000;51(6):193-7 | Full text not available |
| 144. | Hulzebos EH, Smit Y, Helders PP, van Meeteren NL. Preoperative physical therapy for elective cardiac surgery patients. Cochrane Database Syst Rev. 2012;11:Cd010118 | Study type |
| 145. | Hutchinson C. Does prehabilitation before a primary ACL reconstruction improve patient-reported outcome measures? Physiotherapy (United Kingdom). 2020;107 (Supplement 1):e99-e100. | Outcome |
| 146. | Ibrahim MS, Alazzawi S, Nizam I, Haddad FS. An evidence-based review of enhanced recovery interventions in knee replacement surgery. Ann R Coll Surg Engl. 2013;95(6):386-9 | Study type |
| 147. | Ibrahim MS, Khan MA, Nizam I, Haddad FS. Peri-operative interventions producing better functional outcomes and enhanced recovery following total hip and knee arthroplasty: an evidence-based review. BMC Med. 2013;11:37 | Study type |
| 148. | Ibrahim MS, Twaij H, Giebaly DE, Nizam I, Haddad FS. Enhanced recovery in total hip replacement: a clinical review. Bone Joint J. 2013;95-b(12):1587-94 | Study type |
| 149. | Inoue J, Ono R, Makiura D, Kashiwa-Motoyama M, Miura Y, Usami M, et al. Prevention of postoperative pulmonary complications through intensive preoperative respiratory rehabilitation in patients with esophageal cancer. Dis Esophagus. 2013;26(1):68-74 | Outcome |
| 150. | Ismail H, Cormie P, Burbury K, Waterland J, Denehy L, Riedel B. Prehabilitation Prior to Major Cancer Surgery: Training for Surgery to Optimize Physiologic Reserve to Reduce Postoperative Complications. Current Anesthesiology Reports. 2018;8(4):375-85 | Study type |
| 151. | Jankowski CJ. Preparing the Patient for Enhanced Recovery after Surgery. International Anesthesiology Clinics. 2017;55(4):12-20 | Study type |
| 152. | Janssen TL, Mosk CA, van Hoof-de Lepper C, Wielders D, Seerden TCJ, Steyerberg EW, et al. A multicomponent prehabilitation pathway to reduce the incidence of delirium in elderly patients in need of major abdominal surgery: study protocol for a before-and-after study. BMC Geriatr. 2019;19(1):87. | Post-hoc exclusion: No cost outcome in study publication(s) |
| 153. | Jiang DD, Gallagher S, Burchill L, Berli J, Dugi D. Implementation of a pelvic floor physical therapy program for transgender women undergoing gender-affirming vaginoplasty. Journal of Urology. 2019;201(4):e37-e8 | Outcome |
| 154. | Jiang DD, Gallagher S, Burchill L, Berli J, Dugi D. Implementation of a pelvic floor physical therapy program for transgender women undergoing gender-affirming vaginoplasty. Obstetrics and Gynecology. 2019;133(5):1003-11 | Outcome |
| 155. | Jiménez CA, Martín V, Rejas J. Cost-benefit analysis of a smoking cessation program funded by the spanish national system in pre-operative patients. Value in Health. 2017;20(9):A644-A5 | Intervention |
| 156. | Johanning J, Hall D. Successful Prehabilitation: A Noble Goal. Journal of the American Geriatrics Society. 2018;66(10):1869 | Study type |
| 157. | Johansson K, Nuutila L, Virtanen H, Katajisto J, Salantera S. Preoperative education for orthopaedic patients: systematic review. Journal of Advanced Nursing\. 2005;50(2):212-23\ | Study type |
| 158. | Johansson K, Salantera S, Heikkinen K, Kuusisto A, Virtanen H, Leino-Kilpi H. Surgical patient education: assessing the interventions and exploring the outcomes from experimental and quasiexperimental studies from 1990 to 2003. Clinical Effectiveness in Nursing\. 2004;8\:81-92\ | Study type |
| 159. | Jones BD, Jones R, Dunne DF, Astles T, Fenwick SW, Poston GJ, et al. Patient selection and perioperative optimisation in surgery for colorectal liver metastases. European Surgery - Acta Chirurgica Austriaca. 2018;50(3):87-92 | Study type |
| 160. | Jordan RW, Smith NA, Chahal GS, Casson C, Reed MR, Sprowson AP. Enhanced education and physiotherapy before knee replacement; is it worth it? A systematic review. Physiotherapy. 2014;100(4):305-12 | Study type |
| 161. | Judge A, Carr A, Price A, Garriga C, Cooper C, Prieto-Alhambra D, et al. Health Services and Delivery Research. The impact of the enhanced recovery pathway and other factors on outcomes and costs following hip and knee replacement: routine data study. Southampton (UK): NIHR Journals Library | Intervention; Close miss: Pre- and post-operative intervention ("Enhanced recovery after surgery is a complex intervention focusing on several areas of patients’ care pathways through surgery: preoperatively (patient is in best possible condition for surgery), perioperatively (patient has best possible management during and after operation) and postoperatively (patient experiences best rehabilitation).") |
| 162. | Kash BA, Zhang Y, Cline KM, Menser T, Miller TR. The perioperative surgical home (PSH): a comprehensive review of US and non-US studies shows predominantly positive quality and cost outcomes. Milbank Q. 2014;92(4):796-821 | Study type |
| 163. | Kato M, Kito K, Kubo A, Takagi H. Effects of preoperative exercise training on safety and exercise capacity in patients with abdominal aortic aneurysm: A meta-analysis of randomised controlled trials. European Heart Journal. 2018;39:276-7 | Study type |
| 164. | Kato M, Kubo A, Green FN, Takagi H. Meta-analysis of randomized controlled trials on safety and efficacy of exercise training in patients with abdominal aortic aneurysm. J Vasc Surg. 2019;69(3):933-43 | Study type |
| 165. | Katsura M, Kuriyama A, Takeshima T, Fukuhara S, Furukawa TA. Preoperative inspiratory muscle training for postoperative pulmonary complications in adult patients undergoing cardiac and major abdominal surgery. Cochrane Database of Systematic Reviews. 2013;2013(2) | Study type |
| 166. | Kenn K, Sczepanski B. [Pulmonary rehabilitation before and after lung transplantation]. Pneumologie. 2011;65(7):419-27 | Study type |
| 167. | Kim I, Lee H. [Effects of a progressive walking program on physical activity, exercise tolerance, recovery, and post-operative complications in patients with a lung resection]. J Korean Acad Nurs. 2014;44(4):381-90 | Outcome |
| 168. | King PM, Blazeby JM, Ewings P, Longman RJ, Kipling RM, Franks PJ, et al. The influence of an enhanced recovery programme on clinical outcomes, costs and quality of life after surgery for colorectal cancer. Colorectal Disease\. 2006;8(6):506-13\ | Intervention |
| 169. | Klaiber U, Stephan-Paulsen L, Bruckner T, Fink C, Diener MK, Büchler MW, et al. Impact of preoperative patient EDUCATion on the prevention of postoperative complications after major visceral surgery: A randomized controlled pilot trial (PEDUCAT). European Surgical Research. 2017;58:7 | Intervention |
| 170. | Knowles C, Dunne JDF, Ashcroft J, Byrne J, Rigby C, Byrne C, et al. Prehab matters - a prehabilitation service for cancer patients undergoing major abdominal surgery. Physiotherapy (United Kingdom). 2019;105:e132 | Outcome |
| 171. | Kosik SL, Reynolds PJ. A nursing contribution to cost containment. A group preoperative teaching program that shortens hospital stay. J Nurs Staff Dev. 1986;2(1):18-22 | Intervention |
| 172. | Kuhns K. Preoperative thoracic surgery patient education program development. Journal of Thoracic Oncology. 2017;12(1):S198-S9 | Intervention |
| 173. | Kulinski K, Smith NA. Surgical prehabilitation using mobile health coaching in patients with obesity: A pilot study. Anaesth Intensive Care. 2020;48(5):373-80 | Intervention |
| 174. | Langer D. Rehabilitation in Patients before and after Lung Transplantation. Respiration. 2015;89(5):353-62 | Study type |
| 175. | Laurent H, Aubreton S, Galvaing G, Pereira B, Merle P, Richard R, et al. Preoperative respiratory muscle endurance training improves ventilatory capacity and prevents pulmonary postoperative complications after lung surgery. Eur J Phys Rehabil Med. 2020;56(1):73-81 | Outcome |
| 176. | Ledlie N, Gudimetla V. The use of Short Form 12 (SF-12) scores to assess the impact of a pre-habilitation programme. Anaesthesia. 2021;76 (SUPPL 2):151 | Outcome |
| 177. | Lemanu DP, Singh PP, MacCormick AD, Arroll B, Hill AG. Effect of preoperative exercise on cardiorespiratory function and recovery after surgery: a systematic review. World Journal of Surgery\. 2013;37(4):711-20\ | Study type |
| 178. | Lewis D, Fullard K, Kolbe T, Chapman S, Divanoglou A, Doran C, et al. Does face-to-face pre-operative joint replacement education reduce hospital costs in a regional Australian hospital? A descriptive retrospective clinical audit. Eur J Orthop Surg Traumatol. 2020;30(2):257-65 | Intervention; Close miss: Only one (educational) meeting ("The course lasted for approximately 1.5 h, and it was free of charge for those attending.") |
| 179. | Liang MK. Modifying Risk in Ventral Hernia Patients (NCT02365194) Bethesda, United States: NIH U.S. National Library of Medicine; 2018. Available from: https://clinicaltrials.gov/ct2/show/NCT02365194. | Post-hoc exclusion: No cost outcome in study publication(s) |
| 180. | Licker M, Assouline B, Schorer R. Preoperative Exercise Training to Prevent Postoperative Pulmonary Complications in Adults Undergoing Major Surgery: A Systematic Review and Meta-Analysis with Trial Sequential Analysis. Chest. 2020;157 (6 Supplement):A389 | Study type |
| 181. | Lin C, Wan F, Lu Y, Li G, Yu L, Wang M. Enhanced recovery after surgery protocol for prostate cancer patients undergoing laparoscopic radical prostatectomy. J Int Med Res. 2019;47(1):114-21 | Intervention |
| 182. | Liu C, Zhu M. Progress of researches on application of multimodal prehabilitation technique in geriatric surgery [Chinese]. Chinese Journal of Clinical Nutrition. 2020;28(6):368-73 | Full text not available |
| 183. | Lloyd JM, Martin R, Rajagopolan S, Zieneh N, Hartley R. An innovative and cost-effective way of managing ankle fractures prior to surgery--home therapy. Ann R Coll Surg Engl. 2010;92(7):615-8 | Intervention |
| 184. | Loewen I, Jeffery CC, Rieger J, Constantinescu G. Prehabilitation in head and neck cancer patients: a literature review. Journal of Otolaryngology - Head and Neck Surgery. 2021;50(1) | Study type |
| 185. | Lotzke H, Jakobsson M, Brisby H, Gutke A, Hägg O, Smeets R, et al. Use of the PREPARE (PREhabilitation, Physical Activity and exeRcisE) program to improve outcomes after lumbar fusion surgery for severe low back pain: a study protocol of a person-centred randomised controlled trial. BMC Musculoskelet Disord. 2016;17(1):349 | Intervention; Close miss: Pre- and post-operative intervention (post-operative booster session) |
| 186. | Loughney L, Grocott MPW. Exercise and nutrition prehabilitation for the evaluation of risk and therapeutic potential in cancer patients: A review. International Anesthesiology Clinics. 2016;54(4):e47-e61 | Study type |
| 187. | Louw A, Diener I, Landers MR, Puentedura EJ. Preoperative pain neuroscience education for lumbar radiculopathy: a multicenter randomized controlled trial with 1-year follow-up. Spine (Phila Pa 1976). 2014;39(18):1449-57 | Intervention |
| 188. | Louw A, Diener I, Landers MR, Zimney K, Puentedura EJ. Three-year follow-up of a randomized controlled trial comparing preoperative neuroscience education for patients undergoing surgery for lumbar radiculopathy. J Spine Surg. 2016;2(4):289-98 | Intervention |
| 189. | Louw A, Diener I. Preoperative neuroscience education for lumbar radiculopathy patients-a randomised control trial. Physiotherapy (United Kingdom). 2015;101:eS317 | Intervention |
| 190. | Low A, Khan J, Kong KL. Assessment and optimisation of high-risk non-cardiac surgery patients in a joint anaesthetic/cardiology pre-operative clinic. Anaesthesia. 2016;71:57 | Outcome |
| 191. | Lucas B. Does a pre-operative exercise programme improve mobility and function post-total knee replacement: a mini-review. Journal of Orthopaedic Nursing\. 2004;8(1):25-33\ | Study type |
| 192. | Madani A, Fiore JF, Wang Y, Bejjani J, Sivakumaran L, Mata J, et al. An enhanced recovery pathway reduces duration of stay and complications after open pulmonary lobectomy. Surgery (United States). 2015;158(4):899-910 | Intervention |
| 193. | Mahendran K, Naidu B. The key questions in rehabilitation in thoracic surgery. J Thorac Dis. 2018;10(Suppl 8):S924-s30 | Study type |
| 194. | Majerova A. Pre-operative and early postoperative rehabilitation care of patients after total hip joint repla-cement. Rehabilitacia. 2000;33(1):11-23 | Outcome |
| 195. | Makhabah DN, Martino F, Ambrosino N. Peri-operative physiotherapy. Multidiscip Respir Med. 2013;8(1):4 | Study type |
| 196. | Manchester University NHS Foundation Trust. Surviving Aneurysm Surgery: A Pilot Study on Exercise Training in Abdominal Aortic Aneurysm Patients (SAS) (NCT01805973) Bethesda, United States: NIH U.S. National Library of Medicine; 2020. Available from: https://clinicaltrials.gov/ct2/show/NCT01805973. | Post-hoc exclusion: Study never started |
| 197. | Mans CM, Reeve JC, Elkins MR. Postoperative outcomes following preoperative inspiratory muscle training in patients undergoing cardiothoracic or upper abdominal surgery: a systematic review and meta analysis. Clinical Rehabilitation\. 2014:epub\ | Study type |
| 198. | Marcon ER, Baglioni S, Bittencourt L, Lopes CLN, Neumann CR, Trindade MRM. What Is the Best Treatment before Bariatric Surgery? Exercise, Exercise and Group Therapy, or Conventional Waiting: a Randomized Controlled Trial. Obesity Surgery. 2017;27(3):763-73 | Outcome |
| 199. | Martin D, Besson C, Pache B, Michel A, Geinoz S, Gremeaux-Bader V, et al. Feasibility of a prehabilitation program before major abdominal surgery. British Journal of Surgery. 2021;108 (SUPPL 4):iv30 | Outcome |
| 200. | Massierer D, Bourgeois N, Räkel A, Prevost K, Lands LC, Poirier CD, et al. Effectiveness of a Home-Based Pre-Habilitation Program for Lung Transplant Candidates - A Retrospective Chart Review. Journal of Heart and Lung Transplantation. 2020;39(4):S383-S4 | Outcome |
| 201. | Matzi V, Lindenmann J, Muench A, Greilberger J, Juan H, Wintersteiger R, et al. The impact of preoperative micronutrient supplementation in lung surgery. A prospective randomized trial of oral supplementation of combined alpha-ketoglutaric acid and 5-hydroxymethylfurfural. Eur J Cardiothorac Surg. 2007;32(5):776-82 | Outcome |
| 202. | McDonald S, Page Matthew J, Beringer K, Wasiak J, Sprowson A. Preoperative education for hip or knee replacement. Cochrane Database of Systematic Reviews: Reviews\. 2014;Issue 5\ | Study type |
| 203. | McIsaac DI, Hladkowicz E, Bryson GL, Forster AJ, Gagne S, Huang A, et al. Home-based prehabilitation with exercise to improve postoperative recovery for older adults with frailty having cancer surgery: the PREHAB randomised clinical trial. Br J Anaesth. 2022;129(1):41-8. | Post-hoc exclusion: Control group no longer described as usual care, but as an active comparator |
| 204. | McIsaac DI, Saunders C, Hladkowicz E, Bryson GL, Forster AJ, Gagne S, et al. PREHAB study: a protocol for a prospective randomised clinical trial of exercise therapy for people living with frailty having cancer surgery. BMJ Open. 2018;8(6):e022057. | Post-hoc exclusion: Control group no longer described as usual care, but as an active comparator |
| 205. | Merki-Künzli C, Kerstan-Huber M, Switalla D, Gisi D, Raptis DA, Greco N, et al. Assessing the Value of Prehabilitation in Patients Undergoing Colorectal Surgery According to the Enhanced Recovery After Surgery (ERAS) Pathway for the Improvement of Postoperative Outcomes: Protocol for a Randomized Controlled Trial. JMIR Res Protoc. 2017;6(10):e199. | Post-hoc exclusion: No cost outcome in study publication(s) |
| 206. | Milner Q. Fitness for anaesthesia. Foundation Years. 2006;2(4):137-41 | Study type |
| 207. | Minnella EM, Awasthi R, Gillis C, Fiore JF, Jr. , Liberman AS, Charlebois P, et al. Patients with poor baseline walking capacity are most likely to improve their functional status with multimodal prehabilitation. Surgery. 2016;160(4):1070-9 | Outcome |
| 208. | Miralpeix E, Mancebo G, Gayete S, Corcoy M, Solé-Sedeño JM. Role and impact of multimodal prehabilitation for gynecologic oncology patients in an Enhanced Recovery after Surgery (ERAS) program. International Journal of Gynecological Cancer. 2019;29(8):1235-43 | Study type |
| 209. | Mitchell C, Walker J, Walters S, Morgan AB, Binns T, Mathers N. Costs and effectiveness of pre- and post-operative home physiotherapy for total knee replacement: randomized controlled trial. J Eval Clin Pract. 2005;11(3):283-92 | Intervention |
| 210. | Mohamed B, Ramachandran R, Rabai F, Price CC, Polifka A, Hoh D, et al. Frailty Assessment and Prehabilitation Before Complex Spine Surgery in Patients With Degenerative Spine Disease: A Narrative Review. J Neurosurg Anesthesiol.2021 | Full text not available |
| 211. | Moore SM. Effects of interventions to promote recovery in coronary artery bypass surgical patients. Journal of Cardiovascular Nursing\. 1997;12(1):59-70\ | Study type |
| 212. | Morielli AR, Usmani N, Boulé NG, Severin D, Tankel K, Nijjar T, et al. Exercise during and after neoadjuvant rectal cancer treatment (the EXERT trial): study protocol for a randomized controlled trial. Trials. 2018;19(1):35 | Population |
| 213. | Nanavati AJ, Prabhakar S. A comparative study of 'fast-track' versus traditional peri-operative care protocols in gastrointestinal surgeries. J Gastrointest Surg. 2014;18(4):757-67 | Intervention |
| 214. | Nash AB, E OS, Branney D, Anstee L, Thomas A, Hopper I, et al. Does preoperative psychology intervention affect outcomes in hip and knee arthroplasty patients? British Journal of Pain. 2019;13 (14):256-7. | Intervention |
| 215. | National Guideline C. Evidence review for preoperative rehabilitation: Joint replacement (primary): hip, knee and shoulder: Evidence review C. London: National Institute for Health and Care Excellence (UK) Copyright © NICE 2020.; 2020 | Study type |
| 216. | Nielsen PR, Andreasen J, Asmussen M, Tønnesen H. Costs and quality of life for prehabilitation and early rehabilitation after surgery of the lumbar spine. BMC Health Serv Res. 2008;8:209 | Intervention |
| 217. | Nunns M, Shaw L, Briscoe S, Thompson Coon J, Hemsley A, McGrath JS, et al. Health Services and Delivery Research. Multicomponent hospital-led interventions to reduce hospital stay for older adults following elective surgery: a systematic review. Southampton (UK): NIHR Journals Library | Study type |
| 218. | O'Doherty AF, West M, Jack S, Grocott MP. Preoperative aerobic exercise training in elective intra-cavity surgery: a systematic review. Br J Anaesth. 2013;110(5):679-89 | Study type |
| 219. | Onerup A, Angenete E, Bock D, Börjesson M, Fagevik Olsén M, Grybäck Gillheimer E, et al. The effect of pre- and post-operative physical activity on recovery after colorectal cancer surgery (PHYSSURG-C): study protocol for a randomised controlled trial. Trials. 2017;18(1):212 | Intervention |
| 220. | Onerup A, Angerås U, Bock D, Börjesson M, Fagevik Olsén M, Gellerstedt M, et al. The preoperative level of physical activity is associated to the postoperative recovery after elective cholecystectomy - A cohort study. International Journal of Surgery. 2015;19:35-41 | Intervention |
| 221. | Onerup A, Thörn SE, Angenete E, Bock D, Grybäck-Gillheimer E, Haglind E, et al. The effect of pre-and postoperative physical activity on recovery after colorectal cancer surgery (PHYSSURG-C): A randomized controlled trial. Colorectal Disease. 2017;19:141 | Intervention |
| 222. | Oosting E, Hoogeboom T, Appelman S, Dronkers J, Van Meeteren N. Feasibility of an intensive therapeutic exercise program for frail elderly prior to total hip arthroplasty: Two randomized pilot studies. Physiotherapy (United Kingdom). 2015;101:eS1150-eS1 | Outcome |
| 223. | Oosting E, Jans MP, Dronkers JJ, Naber RH, Dronkers-Landman CM, Appelman-de Vries SM, et al. Preoperative home-based physical therapy versus usual care to improve functional health of frail older adults scheduled for elective total hip arthroplasty: a pilot randomized controlled trial. Arch Phys Med Rehabil. 2012;93(4):610-6 | Outcome; Close miss: No cost outcome (only costs of the programme) |
| 224. | Orange ST, Northgraves MJ, Marshall P, Madden LA, Vince RV. Exercise prehabilitation in elective intra-cavity surgery: A role within the ERAS pathway? A narrative review. International Journal of Surgery. 2018;56:328-33 | Study type |
| 225. | Orlovskaya E, Zorin E, Ermakov N, Achkasov E, Yashkov Y. Optimization of perioperative rehabilitation of patients with obesity and severe obstructive sleep apnea syndrome. Obesity Surgery. 2019;29(5):603 | Intervention |
| 226. | Patel A, Mookerji N, Jen T, Lalu M, McIsaac D. A systematic review of perioperative interventions to improve outcomes in frail elderly patients having surgery. Canadian Journal of Anesthesia. 2017;64(1):S72-S3 | Study type |
| 227. | Paterson C, Primeau C, Pullar I, Nabi G. Development of a Prehabilitation Multimodal Supportive Care Interventions for Men and Their Partners Before Radical Prostatectomy for Localized Prostate Cancer. Cancer Nurs. 2019;42(4):E47-e53 | Outcome |
| 228. | Patman S. Preoperative physiotherapy education prevented postoperative pulmonary complications following open upper abdominal surgery. BMJ Evidence-Based Medicine. 2019;24(2):74-5 | Study type |
| 229. | Petro CC, Prabhu AS. Preoperative Planning and Patient Optimization. Surgical Clinics of North America. 2018;98(3):483-97 | Study type |
| 230. | Ploussard G, Loison G, Almeras C, Gautier JR, Cazali P, Tollon C, et al. One-day Prehabilitation Program Before Robotic Radical Prostatectomy in Daily Practice: Routine Feasibility and Benefits for Patients and Hospitals. European Urology Open Science. 2020;21:14-6 | Control |
| 231. | Poulton T, Murray D. Pre-optimisation of patients undergoing emergency laparotomy: a review of best practice. Anaesthesia. 2019;74:100-7 | Study type |
| 232. | Pouwels S, Stokmans RA, Willigendael EM, Nienhuijs SW, Rosman C, van Ramshorst B, et al. Preoperative exercise therapy for elective major abdominal surgery: a systematic review. Int J Surg. 2014;12(2):134-40 | Study type |
| 233. | Pre-surgery fitness programme reduces patients' stay in hospital. Nurs Stand. 2017;31(37):16 | Study type |
| 234. | Preoperative Behavioural Intervention for Risky Drinkers before elective orthopaedic Surgery (Preop-BIRDS). HTA Technology Assessment \. Health Technology Assessment\; 2020 | Intervention |
| 235. | Preoperative physical therapy for severe osteoarthritis of the hip. Lansdale, PA: HAYES Inc\; 2014. | Full text not available |
| 236. | Radboud University Medical Center. Incremental Cost-Utility Study on Prehabilitation Among Older Patients With Colorectal Cancer Undergoing Surgery (PreColo CU) (NCT04097795) Bethesda, United States: NIH U.S. National Library of Medicine; 2019. Available from: https://clinicaltrials.gov/ct2/show/NCT04097795. | Post-hoc exclusion: Study terminated |
| 237. | Rajendran AJ, Pandurangi UM, Murali R, Gomathi S, Vijayan VK, Cherian KM. Pre-operative short-term pulmonary rehabilitation for patients of chronic obstructive pulmonary disease undergoing coronary artery bypass graft surgery. Indian Heart J. 1998;50(5):531-4 | Full text not available |
| 238. | Reilly KA, Beard DJ, Barker KL, Dodd CA, Price AJ, Murray DW. Efficacy of an accelerated recovery protocol for Oxford unicompartmental knee arthroplasty: a randomised controlled trial. Knee \. 2005;12(5):351-7\ | Intervention |
| 239. | Rennie M. Can we keep patients out of the ICU by pre-operative training? British Journal of Intensive Care. 2004;14(2):41. | Full text not available |
| 240. | Riddle DL, Keefe FJ, Ang D, J K, Dumenci L, Jensen MP, et al. A phase III randomized three-arm trial of physical therapist delivered pain coping skills training for patients with total knee arthroplasty: the KASTPain protocol. BMC Musculoskelet Disord. 2012;13:149 | Intervention |
| 241. | Ripollés-Melchor J, Carli F, Coca-Martínez M, Barbero-Mielgo M, Ramírez-Rodríguez JM, García-Erce JA. Committed to be fit. the value of preoperative care in the perioperative medicine era. Minerva Anestesiologica. 2018;84(5):615-25 | Study type |
| 242. | Rispoli M, Salvi R, Cennamo A, Di Natale D, Natale G, Meoli I, et al. Effectiveness of home-based preoperative pulmonary rehabilitation in COPD patients undergoing lung cancer resection. Tumori. 2020:300891619900808 | Outcome |
| 243. | Rivard A, Warren S, Voaklander D, Jones A. The efficacy of pre-operative home visits for total hip replacement clients. Canadian Journal of Occupational Therapy. 2003;70(4):226-32 | Outcome |
| 244. | Rodgers JA, Garvin KL, Walker CW, Morford D, Urban J, Bedard J. Preoperative physical therapy in primary total knee arthroplasty. Journal of Arthroplasty. 1998;13(4):414-21 | Outcome |
| 245. | Rodrigues SN, Henriques HR, Henriques MA. Effectiveness of preoperative breathing exercise interventions in patients undergoing cardiac surgery: A systematic review. Rev Port Cardiol (Engl Ed). 2021;40(3):229-44 | Study type |
| 246. | Rolving N, Nielsen CV, Christensen FB, Holm R, Bünger CE, Oestergaard LG. Does a preoperative cognitive-behavioural intervention affect disability, pain behaviour, pain and return to work after lumbar spinal fusion surgery? European Spine Journal. 2015;24(6):S691. | Intervention |
| 247. | Rolving N, Soegaard R, Nielsen CV, Christensen FB, Bünger CE, Oestergaard LG. Preoperative cognitive-behavioural patient education versus standard care after lumbar spinal fusion: Economic evaluation alongside a randomized controlled trial. European Spine Journal. 2015;24(6):S692 | intervention |
| 248. | Rolving N, Sogaard R, Nielsen CV, Christensen FB, Bunger C, Oestergaard LG. Preoperative cognitive-behavioral patient education versus standard care for lumbar spinal fusion patients: Economic evaluation alongside a randomized controlled trial. Spine. 2016;41(1):18-25 | intervention |
| 249. | Rombey T, Eckhardt H, Quentin W. Cost-effectiveness of prehabilitation prior to elective surgery compared to usual preoperative care: protocol for a systematic review of economic evaluations. BMJ Open. 2020;10(12):e040262 | Study type |
| 250. | Santa Mina D, Clarke H, Ritvo P, Leung YW, Matthew AG, Katz J, et al. Effect of total-body prehabilitation on postoperative outcomes: a systematic review and meta-analysis. Physiotherapy\. 2014;100(3):196-207\ | Study type |
| 251. | Santa Mina D, Scheede-Bergdahl C, Gillis C, Carli F. Optimization of surgical outcomes with prehabilitation. Appl Physiol Nutr Metab. 2015;40(9):966-9 | Study type |
| 252. | Santa Mina D, Sellers D, Au D, Alibhai SMH, Clarke H, Cuthbertson BH, et al. A Pragmatic Non-Randomized Trial of Prehabilitation Prior to Cancer Surgery: Study Protocol and COVID-19-Related Adaptations. Front Oncol. 2021;11:629207 | Control; Close miss: Prehab in both groups (Economic evaluation refers only to the two prehab groups) |
| 253. | Sato T, Gagliardi-Lugo A, Lloyd C, Jones H, King V, Gardner-Thorpe C, et al. A prehabilitation protocol to reduce blood transfusion requirement in head and neck microvascular reconstructive surgery. British Journal of Oral and Maxillofacial Surgery. 2019;57(10):e39 | Intervention |
| 254. | Saunders A. Priming elderly patients for surgery - the ongoing development of a pre-operative service for frail elderly patients. Physiotherapy (United Kingdom). 2020;107 (Supplement 1):e184-e5 | Ouctome |
| 255. | Saunders R, Seaman K, Ashford C, Sullivan T, McDowall J, Whitehead L, et al. An eHealth Program for Patients Undergoing a Total Hip Arthroplasty: Protocol for a Randomized Controlled Trial. JMIR Res Protoc. 2018;7(6):e137 | Intervention |
| 256. | Sawaguchi A, Momosaki R, Hasebe K, Chono M, Kasuga S, Abo M. Effectiveness of preoperative physical therapy for older patients with hip fracture. Geriatrics and Gerontology International. 2018;18(7):1003-8 | Outcome |
| 257. | Schneider S, Armbrust R, Spies C, du Bois A, Sehouli J. Prehabilitation programs and ERAS protocols in gynecological oncology: a comprehensive review. Arch Gynecol Obstet. 2020;301(2):315-26 | Study type |
| 258. | Schwab D, Davies D, Bodtker T, Anaya L, Johnson K, Chaves M. A study of efficacy and cost-effectiveness of guided imagery as a portable, self-administered, presurgical intervention delivered by a health plan. Adv Mind Body Med. 2007;22(1):8-14 | Full text not available |
| 259. | Selvan D, Molloy A, Abdelmalek A, Mulvey I, Alnwick R. The effect of preoperative foot and ankle physiotherapy group on reducing inpatient stay and improving patient care. Foot Ankle Surg. 2013;19(2):118-20 | Outcome |
| 260. | Senkal M, Zumtobel V, Bauer KH, Marpe B, Wolfram G, Frei A, et al. Outcome and cost-effectiveness of perioperative enteral immunonutrition in patients undergoing elective upper gastrointestinal tract surgery: a prospective randomized study. Archives of Surgery\. 1999;134(12):1309-16\ | Intervention |
| 261. | Shenson JA, Craig JN, Rohde SL. Effect of Preoperative Counseling on Hospital Length of Stay and Readmissions after Total Laryngectomy. Otolaryngol Head Neck Surg. 2017;156(2):289-98 | Intervention |
| 262. | Shoemaker MJ, Gibson C, Saagman S. Preoperative exercise in individuals undergoing total knee arthroplasty: state of the evidence. Topics in Geriatric Rehabilitation\. 2013;29(1):2-16\ | Study type |
| 263. | Shuldham CM. Pre-operative education for the patient having coronary artery bypass surgery. Patient Education and Counseling\. 2001;43(2):129-37\ | Study type |
| 264. | Sigurdsson E, Siggeirsdottir K, Jonsson H, Jr. , Gudnason V, Matthiasson T, Jonsson BY. Early discharge and home intervention reduces unit costs after total hip replacement: results of a cost analysis in a randomized study. Int J Health Care Finance Econ. 2008;8(3):181-92 | intervention |
| 265. | Silkman Baker C, McKeon JM. Does preoperative rehabilitation improve patient-based outcomes in persons who have undergone total knee arthroplasty? A systematic review. PM and R\. 2012;4(10):756-67\ | Study type |
| 266. | Silver J. The role of prehabilitation and rehabilitation in high-quality cancer care. Supportive Care in Cancer. 2015;23(1):S23 | Study type |
| 267. | Silver JK, Baima J. Cancer prehabilitation: an opportunity to decrease treatment-related morbidity, increase cancer treatment options, and improve physical and psychological health outcomes. Am J Phys Med Rehabil. 2013;92(8):715-27 | Study type |
| 268. | Silver JK. Cancer prehabilitation and its role in improving health outcomes and reducing health care costs. Semin Oncol Nurs. 2015;31(1):13-30 | Study type |
| 269. | Silver JK. Cancer rehabilitation and prehabilitation may reduce disability and early retirement. Cancer. 2014;120(14):2072-6 | Study type |
| 270. | Simpson A, Howie CR, Kinsella E, Hamilton DF, Conaghan PG, Hankey C, et al. Osteoarthritis Preoperative Package for care of Orthotics, Rehabilitation, Topical and oral agent Usage and Nutrition to Improve ouTcomes at a Year (OPPORTUNITY); a feasibility study protocol for a randomised controlled trial. Trials. 2020;21(1):209 | Ouctome |
| 271. | Singh F, Newton RU, Galvao DA, Spry N, Baker MK. A systematic review of pre-surgical exercise intervention studies with cancer patients. Surgical Oncology\. 2013;22(2):92-104\ | Study type |
| 272. | Sinha A, Jayaraman L, Punhani D, Chowbey P. Enhanced Recovery after Bariatric Surgery in the Severely Obese, Morbidly Obese, Super-Morbidly Obese and Super-Super Morbidly Obese Using Evidence-Based Clinical Pathways: a Comparative Study. Obesity Surgery. 2017;27(3):560-8 | Study type |
| 273. | Snow R, Granata J, Ruhil AV, Vogel K, McShane M, Wasielewski R. Associations between preoperative physical therapy and post-acute care utilization patterns and cost in total joint replacement. J Bone Joint Surg Am. 2014;96(19):e165 | Study type |
| 274. | Snowdon D, Haines TP, Skinner EH. Preoperative intervention reduces postoperative pulmonary complications but not length of stay in cardiac surgical patients: a systematic review. Journal of Physiotherapy\. 2014;60(2):66-77\ | Study type |
| 275. | Sobrinho MT, Guirado GN, Silva MA. Preoperative therapy restores ventilatory parameters and reduces length of stay in patients undergoing myocardial revascularization. Rev Bras Cir Cardiovasc. 2014;29(2):221-8 | Outcome |
| 276. | Sommer MS, Trier K, Vibe-Petersen J, Missel M, Christensen M, Larsen KR, et al. Perioperative rehabilitation in operation for lung cancer (PROLUCA) - rationale and design. BMC Cancer. 2014;14(1) | Outcome |
| 277. | Soni A, Joshi A, Mudge N, Wyatt M, Williamson L. Supervised exercise plus acupuncture for moderate to severe knee osteoarthritis: a small randomised controlled trial. Acupunct Med. 2012;30(3):176-81 | Outcome |
| 278. | Spalding NJ. Reducing anxiety by pre-operative education: Make the future familiar. Occupational Therapy International. 2003;10(4):278-93 | Intervention |
| 279. | Springmeyer SC, Casaburi R, Make B, Jones P, Wise R. Pulmonary rehabilitation and clinical trial design in patients with severe COPD. COPD: Journal of Chronic Obstructive Pulmonary Disease. 2008;5(5):305-9 | Study type |
| 280. | Steffens D, Solomon M, Denehy L. Is preoperative exercise training the new holy grail for patients undergoing major surgery? Annals of the American Thoracic Society. 2021;18(4):587-9. | Study type |
| 281. | Steffens D, Young J, Beckenkamp PR, Ratcliffe J, Rubie F, Ansari N, et al. Feasibility and acceptability of a preoperative exercise program for patients undergoing major cancer surgery: results from a pilot randomized controlled trial. Pilot Feasibility Stud. 2021;7(1):27 | Outcome |
| 282. | SupPoRtive Exercise Programs for Accelerating REcovery after major ABdominal Cancer surgery trial (PREPARE-ABC): pilot phase of a multi-centre randomised controlled trial. Colorectal Dis.2021 | Intervention |
| 283. | Takaoka ST, Weinacker AB. The value of preoperative pulmonary rehabilitation. Thorac Surg Clin. 2005;15(2):203-11 | Study type |
| 284. | Tew GA, Bedford R, Carr E, Durrand JW, Gray J, Hackett R, et al. Community-based prehabilitation before elective major surgery: the PREP-WELL quality improvement project. BMJ Open Qual. 2020;9(1) | Study type |
| 285. | Thomas LC, Patterson M, Dias D, Sumrall D, Schubert A, Chimento G, et al. The perioperative surgical home program improves healcare value and reduces length of stay for patients undergoing elective total hip arthroplasty. Regional Anesthesia and Pain Medicine. 2015;40(5) | Intervention |
| 286. | Thomson K, Pestieau SR, Patel JJ, Gordish-Dressman H, Mirzada A, Kain ZN, et al. Perioperative Surgical Home in Pediatric Settings: Preliminary Results. Anesthesia and Analgesia. 2016;123(5):1193-200 | Intervention |
| 287. | Tibbles L, Lewis C, Reisine S, Rippey R, Donald M. Computer assisted instruction for preoperative and postoperative patient education in joint replacement surgery. Comput Nurs. 1992;10(5):208-12 | Full text not available |
| 288. | Townsend WB, Worrilow WM, Riggs SB. The benefit of prehabilitation and enhanced recovery in robot-assisted radical prostatectomy and the promising future of these protocols in the field of urologic oncology. Cancer. 2020;126(18):4107-9 | Study type |
| 289. | Treanor C, Kyaw T, Donnelly M. An international review and meta-analysis of prehabilitation compared to usual care for cancer patients. J Cancer Surviv. 2018;12(1):64-73 | Study type |
| 290. | Treanor C, Kyaw T, Donnelly M. An international review and metaanalysis of prehabilitation compared to usual care for cancer patients. Psycho-Oncology. 2017;26:59-60 | Study type |
| 291. | Tucker A, McCusker D, Gupta N, Bunn J, Murnaghan M. Orthopaedic Enhanced Recovery Programme for Elective Hip and Knee Arthroplasty - Could a Regional Programme be Beneficial? Ulster Med J. 2016;85(2):86-91. | Intervention |
| 292. | Tully R, Loughney L, Bolger J, Sorensen J, McAnena O, Collins CG, et al. The effect of a pre- and post-operative exercise programme versus standard care on physical fitness of patients with oesophageal and gastric cancer undergoing neoadjuvant treatment prior to surgery (The PERIOP-OG Trial): Study protocol for a randomised controlled trial. Trials. 2020;21(1):638 | Intervention; Close miss: Pre- and post-operative intervention (post-operative booster session) |
| 293. | Ulrich CM, Himbert C, Boucher K, Wetter DW, Hess R, Kim J, et al. Precision-Exercise-Prescription in patients with lung cancer undergoing surgery: rationale and design of the PEP study trial. BMJ Open. 2018;8(12):e024672 | Intervention |
| 294. | Vaillant MF, Albaladejo L, Lathiere T, Thomas-Billot S, Albaladejo V, Proux EA, et al. How to increase adherence to a prehabilitation program: Grenoble's Paprika experience. Clinical Nutrition ESPEN. 2020;40:597 | Outcome |
| 295. | Valkenet K, Trappenburg JC, Gosselink R, Sosef MN, Willms J, Rosman C, et al. Preoperative inspiratory muscle training to prevent postoperative pulmonary complications in patients undergoing esophageal resection (PREPARE study): study protocol for a randomized controlled trial. Trials. 2014;15:144 | Outcome |
| 296. | Valkenet K, van de Port IG, Dronkers JJ, de Vries WR, Lindeman E, Backx FJ. The effects of preoperative exercise therapy on postoperative outcome: a systematic review2011. 99-111\ p. | Study type |
| 297. | van Heusden-Scholtalbers Linda AG, ter Voert Joris M, Staal JB, Bonenkamp Han J, Nijhuis-van der Sanden Maria WG, van Goor H. Preoperative physical exercise training for patients scheduled for major abdominal surgery. Cochrane Database of Systematic Reviews: Reviews\. 2012;Issue 9\ | Study type |
| 298. | Van Looy L, Goethals B. (P)Rehabilitation: Planning preventive, proactive and tailored non pharmacological interventions for cancer patients in a general hospital setting. Psycho-Oncology. 2009;18:S53 | Outcome |
| 299. | VanderBeek S, Kelly R. "get fit" for surgery: Benefits of a prehabilitation clinic for an enhanced recovery program for colorectal surgical patients. Perioperative Medicine. 2016;5 | Intervention |
| 300. | Varela G, Ballesteros E, Jiménez MF, Novoa N, Aranda JL. Cost-effectiveness analysis of prophylactic respiratory physiotherapy in pulmonary lobectomy. Eur J Cardiothorac Surg. 2006;29(2):216-20 | Intervention |
| 301. | Velasco FT, Ko W, Rosengart T, Altorki N, Lang S, Gold JP, et al. Cost containment in cardiac surgery: results with a critical pathway for coronary bypass surgery at the New York Hospital-Cornell Medical Center. Best Practices and Benchmarking in Healthcare\. 1996;1(1):21-8\ | Intervention |
| 302. | Wallis JA, Taylor NF. Pre-operative interventions (non-surgical and non-pharmacological) for patients with hip or knee osteoarthritis awaiting joint replacement surgery - a systematic review and meta-analysis2011. 1381-95\ p. | Study type |
| 303. | Walters TL, Mariano ER, Clark JD. Perioperative Surgical Home and the Integral Role of Pain Medicine. Pain Medicine (United States). 2015;16(9):1666-72 | Study type |
| 304. | Wang L, Lee M, Zhang Z, Moodie J, Cheng D, Martin J. Does preoperative rehabilitation for patients planning to undergo joint replacement surgery improve outcomes? A systematic review and meta-analysis of randomised controlled trials. BMJ Open. 2016;6(2):e009857 | Study type |
| 305. | Wang W, Huang QM, Liu FP, Mao QQ. Effectiveness of preoperative pelvic floor muscle training for urinary incontinence after radical prostatectomy: a meta-analysis. BMC Urology\. 2014;14\:99\ | Study type |
| 306. | Waterland JL, McCourt O, Edbrooke L, Granger CL, Ismail H, Riedel B, et al. Efficacy of Prehabilitation Including Exercise on Postoperative Outcomes Following Abdominal Cancer Surgery: A Systematic Review and Meta-Analysis. Front Surg. 2021;8:628848 | Study type |
| 307. | Weidenhielm L, Mattsson E, Broström LA, Wersäll-Robertsson E. Effect of preoperative physiotherapy in unicompartmental prosthetic knee replacement. Scand J Rehabil Med. 1993;25(1):33-9 | Outcome |
| 308. | Williamson L, Wyatt MR, Yein K, Melton JT. Severe knee osteoarthritis: a randomized controlled trial of acupuncture, physiotherapy (supervised exercise) and standard management for patients awaiting knee replacement. Rheumatology (Oxford). 2007;46(9):1445-9 | Outcome; Close miss: No cost outcome (only costs of the programme) |
| 309. | Wilson CM, Stromberg JS, Wiechec Seidell J. Integrating Prehabilitation, Rehabilitation, and Prospective Surveillance into Cancer Interdisciplinary Teams. Oncology Issues. 2021;36(4):46-55 | Study type |
| 310. | Wright CD, Wain JC, Grillo HC, Moncure AC, Macaluso SM, Mathisen DJ. Pulmonary lobectomy patient care pathway: a model to control cost and maintain quality. Annals of Thoracic Surgery\. 1997;64(2):299-302\ | Intervention |
| 311. | Wu F, Laza-Cagigas R, Pagarkar A, Olaoke A, El Gammal M, Rampal T. The Feasibility of Prehabilitation as Part of the Breast Cancer Treatment Pathway. Pm r.2020 | Outcome; Close miss: No cost outcome (only costs of the programme) |
| 312. | Wu F, Pagarkar A, Olaoke A, Laza-Cagigas R, El Gammal M, Rampal T. Preparing our patients for surgery: A feasibility project following the introduction of a prehabilitation programme for our breast cancer patients. British Journal of Surgery. 2020;107 (SUPPL 4):36 | Control |
| 313. | Wylde V, Marques E, Artz N, Blom A, Gooberman-Hill R. Effectiveness and cost-effectiveness of a group-based pain self-management intervention for patients undergoing total hip replacement: Feasibility study for a randomised controlled trial. Osteoarthritis and Cartilage. 2014;22:S422 | Intervention |
| 314. | Wylde V, Marques E, Artz N, Blom A, Gooberman-Hill R. Effectiveness and cost-effectiveness of a group-based pain self-management intervention for patients undergoing total hip replacement: feasibility study for a randomized controlled trial. Trials. 2014;15:176 | Intervention |
| 315. | Wynter-Blyth V, Moorthy K. Prehabilitation: Preparing patients for surgery. BMJ (Online). 2017;358 | Study type |
| 316. | Yamasaki T, Komasawa N, Omoto H, Kido H, Minami T. Evaluation of lifestyle changes before elective operation: A preliminary observational study. Journal of Clinical Anesthesia. 2016;33:173-5 | Intervention |
| 317. | Yau DKW, Underwood MJ, Joynt GM, Lee A. Effect of preparative rehabilitation on recovery after cardiac surgery: A systematic review. Annals of Physical and Rehabilitation Medicine. 2021;64(2) | Study type |
| 318. | Yeh DD, Martin M, Sakran JV, Meier K, Mendoza A, Grant A, et al. In Brief. Current Problems in Surgery. 2019;56(8):337-9 | Study type |
| 319. | Yogaratnam JZ, Laden G, Guvendik L, Cowen M, Cale A, Griffin S. Hyperbaric oxygen preconditioning improves myocardial function, reduces length of intensive care stay, and limits complications post coronary artery bypass graft surgery. Cardiovasc Revasc Med. 2010;11(1):8-19 | Intervention |
| 320. | Yulia Argunova Y, Pomeshkina SA, Moskin EG, Sogoyan N, Barbarash OL. Effects of prehabilitation program on quality of life and adherence to therapy in patients undergoing coronary artery bypass grafting. European Journal of Preventive Cardiology. 2019;26:S35 | Outcome |
| 321. | Zafirova Z, Urman RD. Preoperative Patient Evaluation: Practicing Evidence-Based, Cost-Effective Medicine. Anesthesiology Clinics. 2018;36(4):xvii-xviii | Study type |
| 322. | Zhou YB. [Prehabilitation for gastrointestinal cancer patients]. Zhonghua Wei Chang Wai Ke Za Zhi. 2021;24(2):122-7 | Full text not available |

### Appendix 5 – Characteristics of ongoing economic evaluations

| **Study ID, main reference** | **Registration number** | **Status*** | **Type and design of analysis** | **Location (city/ cities; Country)** | **Study period**† | **Inclusion criteria (disease(s); type(s) of surgery; criteria for increased perioperative risk)** | **Planned sample size** |
| --- | --- | --- | --- | --- | --- | --- | --- |
| Ongoing studies with published protocol | | | | | | | |
| Barberan-Garcia 2020 (56) | NCT04052100 | unknown | not reported; trial-based (RCT) | Barcelona; Spain | 10/2019-06/2021 | lung cancer; thoracic surgery; impaired predicted lung function and/or ASA score ≥ III and/or age ≥ 75 years | 158 |
| Coca-Martinez 2020 (57) | NCT03466606 | not yet recruiting | not reported; trial-based (RCT) | Barcelona; Spain | 03/2018 -11/2021 | not specified; CABG and/or valve surgery | 160 |
| Pufulete 2020 (58) | ISRCTN10644366 | no longer recruiting | CUA; trial-based (RCT) | Multicentre; UK | 10/2018-03/2022 | not specified; elective major cardiac, thoracic and abdominal surgery; ARISCAT score ≥26 | 2500 |
| Sheill 2020 (60) | NCT03978325 | recruiting | CEA; trial-based (RCT) | Dublin; Ireland | 07/2021-02/2023 | primary oesophageal or lung cancer; oesophagectomy or major lung resection | 78 |
| Schaller 2022 (59) | NCT04418271 | recruiting | CEA; trial-based (RCT) | Multicentre; Germany | 06/2020-11/2023 | not specified; elective surgery with an expected anaesthesia duration > 60 min; age ≥ 70 years, pre-frailty or frailty | 1400 |
| Stamp 2021 (61) | - | no registration number reported | not reported; trial-based (RCT) | not reported, Australia | not reported | not specified; elective cardiac surgery | not reported |
| Steffens 2022 (62) | ACTRN12621000617864 | recruiting | CEA; trial-based (RCT) | Sydney, Melbourne; Australia | 11/2021-09/2024 | advanced or recurrent gastrointestinal cancer; cytoreductive surgery, oesophagectomy, hepatectomy, gastrectomy or pancreatectomy | 172 |
| Svinøy 2019 (63) | NCT03602105 | recruiting | CUA; trial-based (RCT) | South-Eastern Norway Regional Health Authority; Norway | 03/2019-03/2026 | end-stage OA; primary THR; age ≥ 70 years | 150 |
| van Rooijen 2019 (64) | NTR5947, NL5784 | recruiting | CEA, CUA; trial-based (RCT) | Eindhoven-Veldhoven, Netherlands; Montréal, Canada; Roskilde, Denmark; Paris, France; Ferrara, Italy; Barcelona, Spain | not reported-12/2021 | cancer; colorectal resection | 714 |
| West 2021 (65) | NCT03509428 | unknown | CUA; trial-based (RCT) | Southampton, Bournemouth, Portsmouth, Hampshire, Poole, Isle of Wight, Dorset; UK | 03/2018-03/2022 | cancer; major, intra-cavity cancer surgery with a curative intent | 1560 |
| Yau 2019 (66) | ChiCTR1800016098 | recruiting | CUA; trial-based (RCT) | Shatin; Hong Kong | 06/2018-not reported | not specified; CABG, valve replacement or both; pre-frailty or moderate frailty | 164 |
| Ongoing studies without published protocol | | | | | | | |
| Albaladejo 2019 (75) | NCT04190719 | unknown | not reported; trial-based (NRSI) | Grenoble; France | 01/2020-12/2021 | urologic, cardiovascular, thoracic, gastrointestinal disease or orthopaedic disorder; Major surgery; age > 70 years and/or ASA score ≥ III and/or unfit patient according to appropriate scores | 225 |
| Diaz-Feijoo 2021 (67) | NCT04862325 | recruiting | CEA; trial-based (RCT) | Barcelona; Spain | 04/2021-12/2024 | advanced ovarian cancer; gynaecological cancer surgery of high complexity by laparotomy | 146 |
| Hu 2021 (68) | ChiCTR2100042131 | recruiting | not reported; trial-based (RCT) | Ningbo; China | 02/2021-11/2024 | gastric adenocarcinoma; gastric cancer surgery; frailty | 130 |
| Lönnroos 2019 (69) | NCT04001699 | not yet recruiting | CEA; trial-based (RCT) | Kuopio; Finland | 08/2019-12/2024 | hip or knee OA; THA, TKA; multimorbid patient and (at least two) polypharmacy, limited walking distance, care dependency, BMI ≤ 23 or ≥ 34 | 140 |
| McIsaac 2020 (71) | NCT04221295 | recruiting | CEA; trial-based (RCT) | Ottawa; Canada | 02/2020-02/2025 | not specified; major elective non-cardiac surgery; age > 60, frailty | 750 |
| Molenaar 2019 (70) | NL8080 | recruiting | CEA; trial-based (RCT) | Eindhoven, Dordrecht; Netherlands | 01/2020-02/2022 | non-small cell lung cancer; anatomical lung resection | 40 |
| Ocampos 2019 (74) | NCT04017858 | unknown | CUA; trial-based (RCT) | Sao Paulo; Brazil | 08/2019-04/2020 | Knee OA; TKA; age 60-75 years, metabolic syndrome | 44 |
| Santa Mina 2019 (72) | NCT04155346 | not yet recruiting | not reported; trial-based (NRSI) | Toronto; Canada | 11/2019-06/2022 | cancer; cancer surgery; higher-than-average risk, limited physiologic reserve, frail or deconditioned patient, other indications with explanation | 150 |
| Strijker 2020 (73) | NL8699 | recruiting | CEA; trial-based (RCT) | Multicentre; Netherlands | 03/2021 - 03/2023 | various oncological and non-oncological diseases; elective high impact surgery | 2830 |
| * Status according to registration record as per January 16, 2023.  † As stated in the registration record.  Abbreviations: CABG, coronary artery bypass graft; NRSI, non-randomised study of interventions; RCT, randmoised controlled trial; UK, United Kingdom | | | | | | | |

### Appendix 6 – Funding and competing interest of included economic evaluations

| **Study ID, main reference** | **Category of funding** | **Funder** | **Competing interests** |
| --- | --- | --- | --- |
| Completed economic evaluations | | | |
| AlShewaier 2016 (37) | non-profit | Al-Majma’ah University | not reported |
| Barberan-Garcia 2019 (38) | non-profit | European Commission (CONNECARE H2020-689802 and NEXTCARE COMRDI15-1-0016); European Society of Anaesthesiology (ESA Research Support Grant 2016); Instituto de Salud Carlos III (PI15/00576, PI13/00425, and PI12/01241]; Generalitat de Catalunya (2014SGR661). | none |
| Beaupre 2004 (39) | non-profit | Health Research Fund, a division of the Alberta Heritage Foundation for Medical Research, and Capital Health | not reported |
| Chen 2022 (40) | not reported | not applicable | Dr. Ian Randall reports a grant received for implementation of a prehabilitation-related feasibility study from the University Health Network Academic Medical Organization, during the conduct of the study. |
| Dholakia 2021 (41) | not reported | not applicable | none |
| Englesbe 2017 (29) | non-profit | Center for Medicare and Medicaid Services---Health Care Innovation Award 1C1CMS331340-01-00. | M.J.E., J.A.S., W.C.P., N.C.W., and S.C.W. have an equity stake in Prenovo, LLC. |
| Fernandes 2017 (42) | non-profit | OUH Svendborg Hospital (nr. 12/9062) and the Association of Danish Physiotherapists’ Foundation for research, CPD and professional development. | LF is co-owner of Therex Ltd, Denmark. AV is co-owner of Therex Ltd., Denmark. [..] ER is developer of the Good Life with Osteoarthritis in Denmark (GLA:D) program. […] All other authors declare no competing interests. |
| Gao 2015 (43) | not reported | not applicable | none |
| Gränicher 2020 (44) | none | not applicable | none |
| Howard 2019 (30) | non-profit | Blue Cross Blue Shield of Michigan | not reported |
| Huang 2012 (45) | not reported | not applicable | none |
| Koh 2021 (46) | unclear | Altruistic anonymous donor funded the PEERS program. | none |
| Lai 2017 (32) | non-profit | Department of Sichuan Province | none |
| Lai 2019 (33) | not reported | not applicable | none |
| McGregor 2004 (47) | non-profit | National Health Service Executive | not reported |
| Mouch 2019 (31) | non-profit | Center for Medicare and Medicaid Services | not reported |
| Nguyen 2022 (48) | non-profit | French Ministry of Health, Assistance Publique - Hôpitaux de Paris | Dr Roren reported receiving grants from the French Ministry of Health during the conduct of the study. Dr Anract reported receiving grants from Medacata and Grunenthal outside the submitted work and having a patent for SERF with royalties paid. Dr Boisgard reported receiving personal fees from Zimmer and Adler outside the submitted work. Dr Durand-Zaleski reported receiving grants from the Ministry of Health during the conduct of the study. Dr Rannou reported receiving grants from the Programme Hospitalier de Recherche Clinique during the conduct of the study. No other disclosures were reported. |
| Pham 2016 (49) | not reported | not applicable | not reported |
| Ploussard 2020 (50) | none | not applicable | none |
| Risco 2022 (51) | non-profit | JADECARE project- HP-JA-2019 - Grant Agreement no 951442 a European Union’s Health Programm 2014-2020 | none |
| Tew 2017 (52) | non-profit | National Institute for Health Research (NIHR), UK | none |
| Tveter 2020 (53) | non-profit | Interregional research funds (HelSeFOrSK) via the research council of norway | none |
| Van Wijk 2020 (54) | not reported | not applicable | not reported |
| Wang 2020 (55) | not reported | not applicable | none |
| Zhou 2017 (34) | non-profit | Department of Sichuan Province | none |
| Ongoing studies with published protocol | | | |
| Barberan-Garcia 2020 (56) | non-profit | European Commission, Catalan Foundation of Respiratory Medicine, Spanish Respiratory Society | none |
| Coca-Martinez 2020 (57) | mixed | Spanish Public Government Fondos de Investigación en Salud (FIS) Instituto Carlos III, Fresenius Kabi España | none |
| Pufulete 2020 (58) | non-profit | National Institute of Health Research (NIHR) | not reported |
| Schaller 2022 (59) | non-profit | “Innovationsausschuss” of the German Federal Joint Committee (G-BA) | SJS received grants from Reactive Robotics GmbH (Munich, Germany); grants and non-financial support from STIMIT AG (Biel, Switzerland), Liberate Medical LLC (Crestwood USA), and ESICM (Geneva, Switzerland); grants, personal fees, and non-financial support from Fresenius Kabi Deutschland GmbH (Bad Homburg, Germany); personal fees from Springer Verlag GmbH (Vienna, Austria) for educational purposes; and non-financial support from Technical University of Munich (Munich, Germany) and from national and international societies (and their congress organizers) in the field of anesthesiology and intensive care medicine, outside the submitted work. Dr. Schaller held stocks in small amounts from Rhön-Klinikum AG and holds stocks in small amounts from Alphabeth Inc., Bayer AG, and Siemens AG; these holdings have not affected any decisions regarding his research or this study. J Kiselev declares no competing interests. V Loidl declares no competing interests. W Quentin declares no competing interests. K Schmidt declares no competing interests. R Mörgeli declares no competing interests. T Rombey received honorary fees for commissioned research by the Statutory Health Insurance Medical Review Board. R Busse declares no competing interests. U Mannsmann declares no competing interests. C Spies reports grants from Gemeinsamer Bundesausschuss/Federal Joint Committee (G-BA) during the conduct of the study, grants from BMG/RKI, grants from Deutsche Forschungsgemeinschaft/German Research Society, grants from Deutsches Zentrum für Luft- und Raumfahrt e. V. (DLR)/German Aerospace Center, grants from Einstein Stiftung Berlin/Einstein Foundation Berlin, grants from Inneruniversitäre Forschungsförderung/Inner University Grants, grants from Projektträger im DLR/Project Management Agency, grants from Stifterverband/Non-Profit Society Promoting Science and Education, grants from European Society of Anaesthesiology and Intensive Care, grants from Baxter Deutschland GmbH, grants from Cytosorbents Europe GmbH, grants from Edwards Lifesciences Germany GmbH, grants from Fresenius Medical Care, grants from Grünenthal GmbH, grants from Masimo Europe Ltd., grants from Pfizer Pharma PFE GmbH, personal fees from Georg Thieme Verlag, grants from Dr. F. Köhler Chemie GmbH, grants from Sintetica GmbH, grants from Stifterverband für die deutsche Wissenschaft Þ.V./PhilipsÐ grants from Stiftung Charité, grants from AGUETTANT Deutschland GmbH, grants from AbbVie Deutschland GmbH & Co. KG, grants from Amomed Pharma GmbH, grants from InTouch Health, grants from Copra System GmbH, grants from Correvio GmbH, grants from Max-Planck-Gesellschaft zur Förderung der Wissenschaften e.V., grants from Deutsche Gesellschaft für Anästhesiologie & Intensivmedizin (DGAI, grants from Stifterverband für die deutsche Wissen- schaft e.V./Metronic, grants from Philips Electronics Nederland BV, grants from BMG/RKI, grants from BMBF, grants from Deutsche Forschungsgemeinschaft/ German Research Society, and grants from Drägerwerk AG & Co. KGaA, outside the submitted work. In addition, Dr. Spies has a patent 10 2014 215 211.9 licensed, a patent 10 2018 114 364.8 licensed, a patent 10 2018 110 275.5 licensed, a patent 50 2015 010 534.8Ð licensed, a patent 50 2015 010 347.7 licensed, and a patent 10 2014 215 212.7 licensed. |
| Sheill 2020 (60) | non-profit | Health Research Board, Irish Cancer Society | Dr. Emer Guinan is a member of the editorial board (Associate Editor) of this journal. Other authors have no competing interests to disclose. |
| Stamp 2021 (61) | not reported | not applicable | not reported |
| Steffens 2022 (62) | non-profit | Australia’s National Health and Medical Research Council | none |
| Svinøy 2019 (63) | non-profit | Norwegian Fund for Education of Physiotherapists | none |
| van Rooijen 2019 (64) | non-profit | Dutch Cancer Society, Peri-Operative Program | none |
| West 2021 (65) | non-profit | NHS England Sustainability and Transformation Funding, University Hospital Southampton NHS Foundation Trust | none |
| Yau 2019 (66) | non-profit | Department of Anaesthesia and Intensive Care, The Chinese University of Hong Kong | none |
| Ongoing studies without published protocol | | | |
| Albaladejo 2019 (75) | non-profit | University Hospital, Grenoble | not reported |
| Diaz-Feijoo 2021 (67) | non-profit | Hospital Clinic of Barcelona | not reported |
| Hu 2021 (68) | unclear | Zhejiang Medicine and Health Science and Technology Project | not reported |
| Lönnroos 2019 (69) | non-profit | University of Eastern Finland | not reported |
| McIsaac 2020 (71) | non-profit | Ottawa Hospital Research Institute | not reported |
| Molenaar 2019 (70) | non-profit | Máxima MC | not reported |
| Ocampos 2019 (74) | non-profit | University of Sao Paulo General Hospital | not reported |
| Santa Mina 2019 (72) | non-profit | University of Toronto | not reported |
| Strijker 2020 (73) | non-profit | Radboudumc | not reported |

### Appendix 7 – Methods of completed economic evaluations

Legend: * = authors did not respond, † = not enquired from authors, ‡ = information obtained through author contact

AlShewaier 2016 (37)

| **Type and design of analysis** | | CUA; trial-based (RCT) |
| --- | --- | --- |
| **Further references** | | not applicable |
| **Clinical effectiveness** | ***Outcome(s) and measurement*** | QALY: time spent in EQ-5D-5L health states |
|  | ***Valuation*** | not reported* |
|  | ***Source*** | trial documentation |
|  | ***Time horizon (months)*** | 0,5 |
|  | ***Discounting*** | not reported, but assumed no given the time frame of less than 1 year |
| **Costs** | ***Perspective*** | unclear* |
|  | ***Type*** | direct |
|  | ***Calculation approach*** | unclear* |
|  | ***Resource use measurement*** | ACL reconstruction and physiotherapy after surgery, cost of pre-operative physiotherapy rehabilitation |
|  | ***Valuation*** | Costs in GBP |
|  | ***Source*** | unclear* |
|  | ***Time horizon (months)*** | 0,5 |
|  | ***Reference year and currency*** | 2015 Saudi Riyals |
|  | ***Discounting*** | not reported, but assumed no given the time frame of less than 1 year |
|  | ***Correction for inflation*** | not reported, but assumed no given the time frame of recruitment of less than 1 year |
| **Analysis** | ***Description*** | The incremental cost-effectiveness ratio was calculated based on the difference between the two groups in the costs of treatment and in the quality of life (expressed as qualityadjusted life year, QALY) experienced due to the treatment using the following equation (Fenwick et al., 2006). |
|  | ***Model assumptions*** | not applicable |
|  | ***Assessment of uncertainty*** | No sensitivity analysis was included in this assessment. |
|  | ***Handling of missing data*** | not reported* |
|  | ***Willingness-to-pay threshold*** | none available (narrative use of willingness-to-pay thresholds from the United Kingdom and United States of America) |

Barberan-Garcia 2019 (38)

| **Type and design of analysis** | | CCA; trial-based (RCT) |
| --- | --- | --- |
| **Further references** | | Barberan-Garcia A, Ubré M, Roca J, Lacy AM, Burgos F, Risco R, et al. Personalised Prehabilitation in High-risk Patients Undergoing Elective Major Abdominal Surgery: A Randomized Blinded Controlled Trial. Ann Surg. 2017;267(1):50-6. |
| **Clinical effectiveness** | ***Outcome(s) and measurement*** | HrQoL: Self-perceived health status (Short Form 36, 0 (worst) to 100 (best)) Morbidity: rate of postoperative complications (any deviation from the normal postoperative course; classified following the standards of the European Society of Anaesthesiology and European Society of intensive Care Medicine) Mortality: All-cause mortality rate PROMs:  1) Physical activity (Yale Physical Activity Survey, 0 (worst) to indefinite (best)) 2) Psychological status (Hospital anxiety and depression questionnaire, 0 (best) to 21 (worst)) |
|  | ***Valuation*** | not applicable |
|  | ***Source*** | unclear*, assumed: trial documentation |
|  | ***Time horizon (months)*** | Morbidity:  unclear*, assumed until discharge All other outcomes: 6 |
|  | ***Discounting*** | no |
| **Costs** | ***Perspective*** | Mix of payer/ provider perspective |
|  | ***Type*** | direct |
|  | ***Calculation approach*** | bottom-up |
|  | ***Resource use measurement*** | Costs or fees of the pre-habilitation programme and follow-up period; Prehabilitation programme: (i) a cardiopulmonary exercise testing, (ii) the physiotherapist fees, and (iii) a pedometer device. Follow-up postoperative costs (hospitalisation after surgery, hospital readmissions, surgical re-interventions, and emergency room visit costs at 30 days after hospital discharge) included hospital-specific fees of (i) inpatient services, (ii) emergency room visits, (iii) diagnostic procedures, (iv) structural costs and micro costing data on (i) pharmaceutical consumption, (ii) blood products consumption. |
|  | ***Valuation*** | Hospital fees and micro-costing data of the Hospital Clinic de Barcelona, and implied direct cost imputation according to individual consumption at a patient level |
|  | ***Source*** | hospital documentation: diagnostic-related-group-based hospital fees and micro-costing. |
|  | ***Time horizon (months)*** | planned 6, but actually reported for 1 |
|  | ***Reference year and currency*** | 2017 EURO |
|  | ***Discounting*** | no |
|  | ***Correction for inflation*** | not reported* |

Beaupre 2004 (39)

| **Type and design of analysis** | | CMA; trial-based (RCT) |
| --- | --- | --- |
| **Further references** | | not applicable |
| **Costs** | ***Perspective*** | payer perspective |
|  | ***Type*** | direct |
|  | ***Calculation approach*** | bottom-up |
|  | ***Resource use measurement*** | Homecare services measured in terms of hours of provider care, outpatient therapy services were measured by visits to the program, institutional care measured in terms of days of stay: Intervention-related institutional, outpatient therapy and homecare services; institutional services related to transfers to a rehabilitation subacute care program within a continuing care facility, a rehabilitation hospital, or rural acute care hospitals; readmissions to acute care hospitals related to the TKA surgery; physical and occupational therapy services pertaining to direct care or case management in homecare and outpatient therapy programs; personal and home support services, provided by the homecare program. Health service costs were based on the services described above and excluded the cost of the initial surgical stay, which was similar for both study groups. The cost of the preoperative exercise and education program provided to the treatment group was included. |
|  | ***Valuation*** | Standard unit-costs based on Capital Health program costs |
|  | ***Source*** | regional health authorities’ administrative databases |
|  | ***Time horizon (months)*** | 12 |
|  | ***Reference year and currency*** | 1997/98 Canadian dollars |
|  | ***Discounting*** | no |
|  | ***Correction for inflation*** | no; "Unit-costs were expressed in 1997/98 Canadian dollars, and consequently adjustment for price changes over the study period was not necessary." |

Chen 2022 (40)

| **Type and design of analysis** | | CBA; model-based (projection) |
| --- | --- | --- |
| **Further references** | | Santa Mina D, Sellers D, Au D, Alibhai SMH, Clarke H, Cuthbertson BH, et al. A Pragmatic Non-Randomized Trial of Prehabilitation Prior to Cancer Surgery: Study Protocol and COVID-19-Related Adaptations. Frontiers in Oncology. 2021;11. |
| **Clinical effectiveness** | ***Outcome(s) and measurement*** | Morbidity: rate of postoperative complications (surgical site infection, wound disruption, pneumonia, unplanned intubation, on ventilator > 48 h, progressive renal insufficiency, acute renal failure, urinary tract infection, stroke/cerebral vascular accident, cardiac arrest, myocardial infarction, systemic sepsis or septic shock) |
|  | ***Valuation*** | costs |
|  | ***Source*** | American College of Surgeons’ National Surgical Quality Improvement Program database, literature |
|  | ***Time horizon (months)*** | 1 |
|  | ***Discounting*** | no |
| **Costs** | ***Perspective*** | provider perspective |
|  | ***Type*** | direct |
|  | ***Calculation approach*** | top-down |
|  | ***Resource use measurement*** | Fixed and variable programme costs, complications per group |
|  | ***Valuation*** | Costs in USD |
|  | ***Source*** | not reported for intervention costs, Veterans Affairs Decision Support System for cost of morbidity |
|  | ***Time horizon (months)*** | 1 |
|  | ***Reference year and currency*** | 2021 US dollars |
|  | ***Discounting*** | no |
|  | ***Correction for inflation*** | Yes; We adjusted for the cost of inflation, resulting in a median price of US$52,555.77 using the consumer price index (CPI2006 = 198.3, CPI2021 = 273.57, US Bureau of Labor Statistics) to compare costs at the time of the Veterans Affairs analysis in 2006 to costs in 2021. |
| **Analysis** | ***Description*** | The cost-savings attributed to surgical morbidity may be expressed by: MCS = Vuc − Vpc, where MCS represents morbidity cost-savings, u represents the 'usual care' morbidity rate, c represents the median cost of morbidity, and p represents the expected morbidity rate after prehabilitation (calculated using the estimated effect with reported OR and rounded up to be conservative in our estimates). Equations 1.1 and 1.2 may be combined to obtain the total cost-benefit, represented by TCB, of a clinical prehabilitation program budget and expressed by the following equation: TCB = MCS − PC |
|  | ***Model assumptions*** | To achieve an operational financial projection of this clinical prehabilitation program, we assumed an annual patient volume of 240 patients. [...] We calculated financial projections for a clinically integrated prehabilitation program based on the following components detailed in the subsequent sections: (i) prehabilitation costs for multimodal prehabilitation program (exercise, nutrition, psychology) delivery to intra-cavity surgery patients orsurgeries with comparable risk; (ii) NSQIP-based estimates of surgical morbidity rates; (iii) estimated reduction in surgical morbidity by prehabilitation; and (iv) published costs of surgical morbidity. The financial viability of adopting a clinical prehabilitation program was elucidated by calculating an evidence-based projection of cost-benefit, profit-loss relationships, break-even points, and undertaking sensitivity analyses to account for uncertainty in our calculation variables. In the interest of using the most accurate and complete dataset, our calculations are comprised of USD estimates for program delivery and complication-related costs. |
|  | ***Assessment of uncertainty*** | A total of three sensitivity analyses were conducted to account for the uncertainty in the assumptions underlying our financial projections. |
|  | ***Handling of missing data*** | not applicable, model |
|  | ***Willingness-to-pay threshold*** | not applicable (perfomed break even analysis) |

Dholakia 2021 (41)

| **Type and design of analysis** | | CEA; model-based (decision tree) |
| --- | --- | --- |
| **Further references** | | Dholakia J, Cohn DE, Montemorano L, Straughn JM, Dilley SE. Prehabilitation is a cost-saving method with improved outcomes for medically frail patients undergoing surgery for epithelial ovarian cancer: A cost-effectiveness analysis. Gynecologic Oncology. 2020;159 (Supplement 1):74. |
| **Clinical effectiveness** | ***Outcome(s) and measurement*** | Mortality*: rate following major complication (perioperative complication rates and 90-day survival estimates from the literature (Kumar et al. 2017, Shinall et al. 2020, Kniesely et al. 2020)) |
|  | ***Valuation*** | life years gained |
|  | ***Source*** | literature |
|  | ***Time horizon (months)*** | 12 |
|  | ***Discounting*** | not reported* |
| **Costs** | ***Perspective*** | payer perspective |
|  | ***Type*** | direct |
|  | ***Calculation approach*** | top-down |
|  | ***Resource use measurement*** | Estimates from the literature: Hospital charge [32, 33, 19, 35], non-home discharge facility charge (w/wo complications) [29,30], pre-habilitation program [20, 24, 36], usual preoperative care (For routine pre-operative costs, we assumed that patients received laboratory testing (complete blood count, Chem-10) and/or basic diagnostics (chest x-ray, electrocardiogram) costing on average $200 per patient [no source provided]. |
|  | ***Valuation*** | Ohio area estimates of per diem costs for a semi-private nursing home room [15,16,34]; |
|  | ***Source*** | Estimates from the literature and Diagnostic Related Grouping (DRG) cost estimates: DRG Code 738: ‘Hospital charge: Uterine and adnexa procedure for ovarian or adnexal malignancy without complication,’; Code 736: ‘Hospital charge: Uterine and adnexa procedure for ovarian or adnexal malignancy with major complication.’ |
|  | ***Time horizon (months)*** | 12 |
|  | ***Reference year and currency*** | US dollars (year not reported)* |
|  | ***Discounting*** | not reported* |
|  | ***Correction for inflation*** | not reported* |
| **Analysis** | ***Description*** | We developed a decision tree model to evaluate the cost-effectiveness of a prehabilitation intervention on healthcare system costs for medically frail women undergoing PDS for EOC. The primary outcome was incremental cost-effectiveness ratio (ICER) of prehabilitation compared to no pre-operative intervention. The ICER represents the cost per one life year gained with prehabilitation compared to standard of care; a lower ICER represents a cost-effective strategy. For this model, we utilized a conservative standard willingness-to-pay ratio of $100,000 per life year; this represents the amount that society is willing to pay for one additional year of life [25]. |
|  | ***Model assumptions*** | We applied our model to a hypothetical cohort of 4,415 medically frail women who undergo PDS for EOC in one year. Of the estimated 22,530 women newly diagnosed with ovarian cancer annually [26], 55%-80% of these women will undergo PDS [27]. A recent systematic review identifed the prevalence of frailty in gynecologic oncology patients ranges between 6.1%-60%; We approximated that a prevalence of 24.5% based on data from the Mayo Clinic [6, 8, 28, 29]. Probabilities of a major complication, death from major complication, and need for discharge to an increased level of care facility were included in the model (Fig. 1). |
|  | ***Assessment of uncertainty*** | One-way sensitivity analyses were performed to account for uncertainty in our model inputs. First, a simultaneous one-way sensitivity analysis was run in a tornado analysis to demonstrate the variables with the largest impact on the ICER. Threshold values were then calculated to determine the probabilities of these variables at which the preferred strategy would change. |
|  | ***Handling of missing data*** | not applicable, model |
|  | ***Willingness-to-pay threshold*** | 100,000 $ per life year gained |

Englesbe 2017 (29)

| **Type and design of analysis** | | CMA; trial-based (NRSI) |
| --- | --- | --- |
| **Further references** | | not applicable |
| **Costs** | ***Perspective*** | Mix of payer/ provider perspective |
|  | ***Type*** | direct |
|  | ***Calculation approach*** | bottom-up and top-down |
|  | ***Resource use measurement*** | LOS, total cost, and payer costs for the primary encounter and for all hospital encounters within 90 days from the primary encounter were considered. Total cost was computed as the sum of direct (patient care) and indirect (overhead) hospital costs as calculated by the cost accounting system. Payer costs were determined based on “estimated payment” for the admission based on the patient’s primary insurance. To remove surgeon-to-surgeon variation in billing, professional fees were not included in the analysis. |
|  | ***Valuation*** | Costs were inﬂation adjusted to January 2015 US dollars using the Bureau of Labor Statistics’ consumer price index |
|  | ***Source*** | University of Michigan Health System Data Warehouse cost accounting system for both the treatment and control groups |
|  | ***Time horizon (months)*** | 3 |
|  | ***Reference year and currency*** | 2015 US dollars |
|  | ***Discounting*** | not reported, but assumed no given the time frame of less than 1 year |
|  | ***Correction for inflation*** | Costs were inflation adjusted to January 2015 dollars using the Bureau of Labor Statistics’ consumer price index. |

Fernandes 2017 (42)

| **Type and design of analysis** | | CUA; trial-based (RCT) |
| --- | --- | --- |
| **Further references** | | Fernandes L, Roos EM, Overgaard S, Villadsen A, Soegaard R. Supervised neuromuscular exercise prior to hip or knee replacement: Cost-utility analysis alongside a randomised controlled trial. Osteoarthritis and Cartilage. 2015;23:A35-A6. |
| **Clinical effectiveness** | ***Outcome(s) and measurement*** | QALY: time spent in EQ-5D-3L health states |
|  | ***Valuation*** | Health state valuations from the Danish general population |
|  | ***Source*** | trial documentation |
|  | ***Time horizon (months)*** | 14 |
|  | ***Discounting*** | no |
| **Costs** | ***Perspective*** | Mix of payer/ provider/ patient perspective |
|  | ***Type*** | direct and indirect |
|  | ***Calculation approach*** | bottom-up |
|  | ***Resource use measurement*** | Intervention & hospital-based utilization and costs: Services provided in primary care (NHISR, in national reimbursement fees per visit); contacts (visits and inpatient days) to hospitals including diagnoses, procedures (DNPR: DRG case-mix tariffs); intervention costs based (RCT-data: exercise sessions á 1.25 h). Post-operative municipality-based care utilization and costs were not included.  Patient expenses (RCT-data, Statistic Denmark): travelling distance and time (transportation costs in national fees for travel reimbursement), informal time (productivity loss) measured by person’s time and wage rates (age- and gender matched national average gross income for year 2012) |
|  | ***Valuation*** | Tariff-based fees for physiotherapy in primary care 2016 expressed in 2012 Euro (1 EUR = DKK 7.45) Health care utilization valued by national reimbursement fees and DRG case-mix tariffs Valuation of patients’ costs by human capital approach: national fees for travel reimbursement for 2012 (DKK 3.80/km or €0.51/km) times the distance (km) between the exercise facility and patients’ homes. |
|  | ***Source*** | National Health Insurance Service Registry, Danish National Patient Register, Statistic Denmark, RCT-data) |
|  | ***Time horizon (months)*** | 14 |
|  | ***Reference year and currency*** | 2012 EURO |
|  | ***Discounting*** | no |
|  | ***Correction for inflation*** | not reported* |
| **Analysis** | ***Description*** | QALYs were produced by calculating the area under the curve of the EQ-5D-3L utility scores from baseline and all follow-ups assuming linear trend between observations. One-year clinical effect was expressed as the between-group mean difference [95% confidence interval (CI)] of change values (61 weeks – baseline) and effect-size (d = mean difference of change values/pooled baseline standard deviation) of the five subscale scores of the HOOS and KOOS. Analysis of linear regression was used for between-group comparisons of QALYs and costs and presented as between-group mean differences (95% CI) over the time horizon. An adjustment for baseline health utility was included in the analysis to account for baseline imbalances in the estimation of mean differential QALYs. We estimated the value for money of the intervention by calculating the incremental net monetary benefit using a range of hypothetical threshold values for decision-makers’ willingness-to-pay for a unit of effect [32]. The threshold values ranged from €0 to €100,000. The net benefits were presented visually in cost-effectiveness acceptability curves (CEAC). |
|  | ***Model assumptions*** | not applicable |
|  | ***Assessment of uncertainty*** | All parameters were tested for normality and distribution. Because of skewed data all comparative analyses, including the net benefit, were based on bootstrapped standard errors. Non-parametric bootstrapping with 10,000 replications was applied. |
|  | ***Handling of missing data*** | linear trend at point (LTAP) |
|  | ***Willingness-to-pay threshold*** | 40,000€ per QALY gained |

Gao 2015 (43)

| **Type and design of analysis** | | CCA; trial-based (NRSI) |
| --- | --- | --- |
| **Further references** | | not applicable |
| **Clinical effectiveness** | ***Outcome(s) and measurement*** | Morbidity: Rate of postoperative complications (diarrhea, allergy, aerodermectasia, arrhythmia, aconuresis, aerothorax, pleural effusion, lung infection) |
|  | ***Valuation*** | not applicable |
|  | ***Source*** | not reported* |
|  | ***Time horizon (months)*** | unclear*; assumed until discharge |
|  | ***Discounting*** | not reported, but assumed no given the time frame of less than 1 year |
| **Costs** | ***Perspective*** | unclear* |
|  | ***Type*** | direct |
|  | ***Calculation approach*** | unclear* |
|  | ***Resource use measurement*** | Average hospital costs included the costs of examinations, surgery, drugs, and the cost of staying in a ward. Pulmonary rehabilitation costs included the costs of the test of cardiopulmonary function, physical treatment, drugs, and the cost of the volumetric exerciser. |
|  | ***Valuation*** | not reported* |
|  | ***Source*** | not reported* |
|  | ***Time horizon (months)*** | until discharge |
|  | ***Reference year and currency*** | Yuan (year not reported)* |
|  | ***Discounting*** | not reported, but assumed no given the time frame of less than 1 year |
|  | ***Correction for inflation*** | not reported* |

Gränicher 2020 (44)

| **Type and design of analysis** | | CCA; trial-based (RCT) |
| --- | --- | --- |
| **Further references** | | not applicable |
| **Clinical effectiveness** | ***Outcome(s) and measurement*** | PROMs: 1) Patients’ perception of knee function and activity level in activities of daily living (Lysholm Score, 3 (worst) to 100 (best)) 2) Pain (item from Lysholm Score, 0 (worst) to 25 (best)) 3) Assessment of work and sporting activities (Tegner Activity Scale, 0 (worst) to 10 (best))  Physical funtion: 1) Stair Climbing Test (time used to ascend and descend a flight of eight 16 cm high steps with a depth of 30cm in seconds) 2) Range of Motion (degrees measured by standard goniometer) |
|  | ***Valuation*** | not applicable |
|  | ***Source*** | trial documentation |
|  | ***Time horizon (months)*** | 3 |
|  | ***Discounting*** | not reported, but assumed no given the time frame of less than 1 year |
| **Costs** | ***Perspective*** | payer perspective‡ |
|  | ***Type*** | direct |
|  | ***Calculation approach*** | bottom-up |
|  | ***Resource use measurement*** | total treatment costs‡ (based on the amount of preoperative therapy sessions and prescribed length of stay at an inpatient rehabilitation facility); (patient) total costs (e.g. preoperative therapies, walking aids, medication) prior to surgery, costs of postoperative medication and post-acute care services (e.g. payment for home health agencies) and amount of rehabilitative ambulant physiotherapy sessions:  "costs of pre- and postoperative therapy sessions (see https://zh-gl.physioswiss.ch/de/tarif/tarifstruktur) à physical therapists · additional costs (if not within usual care, e.g. braces, aids for everyday life, costs for unplanned medical consultations associated with TKA, …) à material costs, orthopeadists/CP · average costs per week at rehabilitation clinic (e.g. santésuisse) à includes all medical-therapeutic services during stay" |
|  | ***Valuation*** | actual payments by patients‡, valuation of primary or secondary care utilization not reported: "costs of pre- and postoperative therapy sessions (see https://zh-gl.physioswiss.ch/de/tarif/tarifstruktur) à physical therapists · additional costs (if not within usual care, e.g. braces, aids for everyday life, costs for unplanned medical consultations associated with TKA, …) à material costs, orthopeadists/CP · average costs per week at rehabilitation clinic (e.g. santésuisse) à includes all medical-therapeutic services during stay" |
|  | ***Source*** | patient diary; clinic information system |
|  | ***Time horizon (months)*** | 3 |
|  | ***Reference year and currency*** | CHF (2017)‡ |
|  | ***Discounting*** | not reported, but assumed no given the time frame of less than 1 year |
|  | ***Correction for inflation*** | No‡; "costs were not corrected for inflation" |

Howard 2019 (30)

| **Type and design of analysis** | | CCA; trial-based (NRSI) |
| --- | --- | --- |
| **Further references** | | not applicable |
| **Clinical effectiveness** | ***Outcome(s) and measurement*** | Morbidity: Rate of postoperative complications (classified as per Clavien-Dindo; grouped as none, minor (class I-II), major (class III-IV), and death (class V)) Mortality: rate |
|  | ***Valuation*** | not applicable |
|  | ***Source*** | medical charts |
|  | ***Time horizon (months)*** | 1‡ |
|  | ***Discounting*** | not reported, but assumed no given the time frame of less than 1 year |
| **Costs** | ***Perspective*** | Mix of payer/ provider perspective |
|  | ***Type*** | direct |
|  | ***Calculation approach*** | bottom-up |
|  | ***Resource use measurement*** | The primary financial outcomes included hospital charges, professional charges, and total charges for each patient. |
|  | ***Valuation*** | actual charges in 2012–2017 US dollars |
|  | ***Source*** | Health record system‡: "The data source was our electronic health record system (so this was essentially a “chart review”). Clinical data were abstracted directly from patients’ medical records and physiologic data were abstracted from anesthesia records. Cost data were also from our electronic health record system." |
|  | ***Time horizon (months)*** | until discharge |
|  | ***Reference year and currency*** | US dollars (2012-2017)‡ |
|  | ***Discounting*** | not reported, but assumed no given the time frame of less than 1 year |
|  | ***Correction for inflation*** | no‡; "They were not adjusted for inflation." |

Huang 2012 (45)

| **Type and design of analysis** | | CCA; trial-based (RCT) |
| --- | --- | --- |
| **Further references** | | not applicable |
| **Clinical effectiveness** | ***Outcome(s) and measurement*** | Morbidity:  1) rate of infection 2) rate of deep vein thrombosis PROMs: Pain (visual analogue scale, 0 (best) to 10 (worst)) Physical function: 1) knee range of motion (degrees) 2) ambulation status (positive when patient can ambulate independently for more than 15 meters) |
|  | ***Valuation*** | not applicable |
|  | ***Source*** | trial documentation |
|  | ***Time horizon (months)*** | Morbidity: Until discharge All other outcomes: 5 days |
|  | ***Discounting*** | not reported, but assumed no given the time frame of less than 1 year |
| **Costs** | ***Perspective*** | provider perspective |
|  | ***Type*** | direct |
|  | ***Calculation approach*** | bottom-up‡ |
|  | ***Resource use measurement*** | Medical cost was calculated as the total medical expenditure of hospitalization for TKA, including preoperative care, prosthesis, operation, and post-TKA costs.‡ "We calculate the cost by summing up all the costs (by every item) per participant. (Because our insurance system was paid per patient.)" |
|  | ***Valuation*** | Costs in NTD |
|  | ***Source*** | medical information system |
|  | ***Time horizon (months)*** | until discharge |
|  | ***Reference year and currency*** | NTD (2008-2010)‡ |
|  | ***Discounting*** | not reported, but assumed no given the time frame of less than 1 year |
|  | ***Correction for inflation*** | no‡; "Not corrected for inflation." |

Koh 2021 (46)

| **Type and design of analysis** | | CCA; trial-based (NRSI) |
| --- | --- | --- |
| **Further references** | | Koh FH, Tan WJ, Ho L, Yen F, G K, Sivarajah S, et al. QS342: A structured pre-surgery prehabilitation for elderly patients undergoing elective surgery significantly improves surgical outcomes and reduces cost. Dis Colon Rectum. 2020;63(6):e125. |
| **Clinical effectiveness** | ***Outcome(s) and measurement*** | Morbidity: Rate of postoperative complications (classified as per Clavien-Dindo) Mortality: rate  Note: Survival was also calculated but not mentioned in the methods as an outcome and thus not further considered. |
|  | ***Valuation*** | not applicable |
|  | ***Source*** | electronic medical records; National Death Registry |
|  | ***Time horizon (months)*** | 1 |
|  | ***Discounting*** | not reported, but assumed no given the time frame of less than 1 year |
| **Costs** | ***Perspective*** | patient perspective‡ |
|  | ***Type*** | direct‡ |
|  | ***Calculation approach*** | top-down |
|  | ***Resource use measurement*** | national average unsubsidized cost per day for a colonic resection in a public hospital‡: "We actually did not look at the breakdown of cost due to prevailing Personal data protection Act laws in the country. However, what we did was to look at the number of days of hospitalisations savings, and then looking at the fee benchmarks for a paying patient (A class patient) for 1 day stay in a general ward and multiple that by the number of days saved, this gave us a MINIMUM cost savings of USD$11,838.80 per patient with an average of 800 dollars spending per patient on the prehabilitation interventions)." |
|  | ***Valuation*** | cost savings in USD |
|  | ***Source*** | Medical chart, Ministry of Health, Singapore, Fee benchmarks and Bill Amount Information, 2019 |
|  | ***Time horizon (months)*** | until discharge |
|  | ***Reference year and currency*** | US dollars (2019)‡ |
|  | ***Discounting*** | not reported, but assumed no given the time frame of less than 1 year |
|  | ***Correction for inflation*** | no‡; "the cost was not [corrected for inflation] as it was obtained in the same year of writing and not publication." |

Lai 2017 (32)

| **Type and design of analysis** | | CCA; trial-based (RCT) |
| --- | --- | --- |
| **Further references** | | Lai Y, Su J, Yang M, Zhou K, Che G. [Impact and Effect of Preoperative Short-term Pulmonary Rehabilitation Training on  Lung Cancer Patients with Mild to Moderate Chronic Obstructive Pulmonary Disease:  A Randomized Trial]. Zhongguo Fei Ai Za Zhi. 2016;19(11):746-753. Huang J, Lai Y, Zhou X, Li S, Su J, Yang M, et al. Short-term high-intensity rehabilitation in radically treated lung cancer: a three-armed randomized controlled trial. J Thorac Dis. 2017;9(7):1919-29. |
| **Clinical effectiveness** | ***Outcome(s) and measurement*** | Morbidity: rate of postoperative pulmonary complications (atelectasis, acute respiratory distress syndrome, respiratory failure, mechanical ventilation at 48 h postoperatively, deep vein thrombosis/pulmonary embolism, empyema or pneumonia) |
|  | ***Valuation*** | not applicable |
|  | ***Source*** | trial documentation |
|  | ***Time horizon (months)*** | 1 |
|  | ***Discounting*** | not reported, but assumed no given the time frame of less than 1 year |
| **Costs** | ***Perspective*** | unclear* |
|  | ***Type*** | unclear*; assumed direct |
|  | ***Calculation approach*** | not reported* |
|  | ***Resource use measurement*** | Unclear*; “In-hospital expenses included a daily nursing care fee, intraoperative examination cost, surgery-related expenses, drug costs and so forth.” |
|  | ***Valuation*** | not reported |
|  | ***Source*** | not reported* |
|  | ***Time horizon (months)*** | until discharge |
|  | ***Reference year and currency*** | EUR (year not reported)* |
|  | ***Discounting*** | not reported, but assumed no given the time frame of less than 1 year |
|  | ***Correction for inflation*** | not reported* |

Lai 2019 (33)

| **Type and design of analysis** | | CCA; trial-based (RCT) |
| --- | --- | --- |
| **Further references** | | not applicable |
| **Clinical effectiveness** | ***Outcome(s) and measurement*** | Morbidity: rate of postoperative pulmonary complications (using definitions by Society of Thoracic Surgeons, European Society of Thoracic Surgeons, Centers for Disease Control (for pneumonia); classified as per Clavien-Dindo) |
|  | ***Valuation*** | not applicable |
|  | ***Source*** | trial documentation |
|  | ***Time horizon (months)*** | unclear*; assumed until discharge |
|  | ***Discounting*** | not reported, but assumed no given the time frame of less than 1 year |
| **Costs** | ***Perspective*** | unclear* |
|  | ***Type*** | unclear*; assumed direct |
|  | ***Calculation approach*** | not reported* |
|  | ***Resource use measurement*** | Details unclear*; “Total expense included all the in-hospitalization costs, in-hospital expense included the costs during the in-hospital stay, and material or drug cost include expense for all the material or drug use during in-hospitalization.” |
|  | ***Valuation*** | not reported |
|  | ***Source*** | not reported* |
|  | ***Time horizon (months)*** | until discharge |
|  | ***Reference year and currency*** | Yuan (year not reported)* |
|  | ***Discounting*** | not reported, but assumed no given the time frame of less than 1 year |
|  | ***Correction for inflation*** | not reported* |

McGregor 2004 (47)

| **Type and design of analysis** | | CEA; trial-based (RCT) |
| --- | --- | --- |
| **Further references** | | not applicable |
| **Clinical effectiveness** | ***Outcome(s) and measurement*** | HrQoL: EQ-5D-3L (visual analogue scale, 0% (worst) to 100% (best); utility score‡, 0 (worst) to 1 (best)) |
|  | ***Valuation*** | “we used the EQ5D scoring calculator that was available at the time”‡ |
|  | ***Source*** | trial documentation |
|  | ***Time horizon (months)*** | 3 |
|  | ***Discounting*** | not reported, but assumed no given the time frame of less than 1 year |
| **Costs** | ***Perspective*** | Mix of payer/ provider perspective |
|  | ***Type*** | direct |
|  | ***Calculation approach*** | top-down‡ |
|  | ***Resource use measurement*** | Costs incurred to the National Health Service including staffing costs (except the surgical team): cost of the preadmission class and booklet where appropriate, the cost of the hospital stay, inpatient physiotherapy, and occupational therapy costs, outpatient costs, visits to the general practitioner, and use of community or outpatient therapy. The costs of equipment and medication were not included because these costs were deemed to be similar in the 2 study populations. |
|  | ***Valuation*** | Costs in GBP |
|  | ***Source*** | Unclear; could not be reconstructed‡ |
|  | ***Time horizon (months)*** | 3 |
|  | ***Reference year and currency*** | GBP (2003)‡ |
|  | ***Discounting*** | not reported, but assumed no given the time frame of less than 1 year |
|  | ***Correction for inflation*** | not reported* |
| **Analysis** | ***Description*** | The data generated were analyzed using an unbalanced analysis of variance with restricted maximum likelihood using Genstat Release 4.1 (Oxford, UK). The statistical threshold was set at P<.05. The model contained the fixed effects for the study group (patients receiving preoperative advice and exercise, Group A, and patients who did not, Group B), time (admission, discharge, 3 months after surgery), age, and interaction. Wald tests were performed to assess the significance of the fixed effects. The baseline preadmission assessment was included in the model as a covariate to reduce the variability among subjects. The model contained a random effect for subject. |
|  | ***Model assumptions*** | not applicable |
|  | ***Assessment of uncertainty*** | unclear; could not be reconstructed‡ |
|  | ***Handling of missing data*** | not applicable‡ |
|  | ***Willingness-to-pay threshold*** | unclear; could not be reconstructed‡ |

Mouch 2019 (31)

| **Type and design of analysis** | | CMA; trial-based (NRSI) |
| --- | --- | --- |
| **Further references** | | not applicable |
| **Costs** | ***Perspective*** | Mix of payer/ provider perspective |
|  | ***Type*** | direct |
|  | ***Calculation approach*** | bottom-up |
|  | ***Resource use measurement*** | total episode payments, index hospitalization payments, and 90-day post-acute care payments: inpatient (initial admission/readmission), outpatient, professional fees (in-/outpatient), carrier, durable medical equipment, skilled nursing facility, home health services |
|  | ***Valuation*** | actual Medicare payment documented on the claim (actual claims in 2014–2017 US dollars) |
|  | ***Source*** | Centers for Medicare and Medicaid Services (data use agreement on ﬁle: any cell displaying data of n <11 is masked) |
|  | ***Time horizon (months)*** | 3 |
|  | ***Reference year and currency*** | US dollars (year not reported)* |
|  | ***Discounting*** | not reported, but assumed no given the time frame of less than 1 year |
|  | ***Correction for inflation*** | not reported* |

Nguyen 2022 (48)

| **Type and design of analysis** | | CUA; trial-based (RCT) |
| --- | --- | --- |
| **Further references** | | not applicable |
| **Clinical effectiveness** | ***Outcome(s) and measurement*** | QALY: time spent in EQ-5D-3L health states |
|  | ***Valuation*** | unclear† |
|  | ***Source*** | trial documentation |
|  | ***Time horizon (months)*** | 12 |
|  | ***Discounting*** | not reported |
| **Costs** | ***Perspective*** | Mix of payer/ provider perspective |
|  | ***Type*** | direct |
|  | ***Calculation approach*** | bottom-up |
|  | ***Resource use measurement*** | time spent before TKR by the surgeons in the control group and by the teams in charge of education and rehabilitation in the experimental group, the duration of hospitalization, and the length of stay in the rehabilitation department |
|  | ***Valuation*** | gross salary of involved health care professionals, diagnosis-related group cost adjusted for actual length of stay |
|  | ***Source*** | case report form |
|  | ***Time horizon (months)*** | not reported†; assumed until discharge from rehabilitation |
|  | ***Reference year and currency*** | EUR (year not reported)† |
|  | ***Discounting*** | not reported |
|  | ***Correction for inflation*** | not reported† |
| **Analysis** | ***Description*** | Costs were calculated per patient and compared by randomization group based on intention to treat, using bootstrap hypothesis testing to avoid relying on normality assumptions. |
|  | ***Model assumptions*** | not applicable |
|  | ***Assessment of uncertainty*** | bootstrapping |
|  | ***Handling of missing data*** | not reported† |
|  | ***Willingness-to-pay threshold*** | none stated |

Pham 2016 (49)

| **Type and design of analysis** | | CMA; trial-based (RCT) |
| --- | --- | --- |
| **Further references** | | not applicable |
| **Costs** | ***Perspective*** | unclear* |
|  | ***Type*** | direct |
|  | ***Calculation approach*** | bottom-up and top-down |
|  | ***Resource use measurement*** | costs to deliver the prehabilitation exercise program (facility  costs to deliver the land and pool based exercise program, the salaries of staff to  administer the program, the equipment, licensing and administration supplies),  hospital costs associated with TJA; recovery cost per day (i.e. fees incurred during the hospital stay: the effect of a prehabilitation program on LOS post surgery and the hospital cost per day including salary costs of physicians and nurses, medication, food, laundry and so forth) |
|  | ***Valuation*** | Costs in CAD |
|  | ***Source*** | unclear*; assumed hospital documentation |
|  | ***Time horizon (months)*** | unclear if 3 or only until discharge* |
|  | ***Reference year and currency*** | CAD (year not reported)* |
|  | ***Discounting*** | not reported, but assumed no given the time frame of less than 1 year |
|  | ***Correction for inflation*** | not reported* |

Ploussard 2020 (50)

| **Type and design of analysis** | | CCA; trial-based (NRSI) |
| --- | --- | --- |
| **Further references** | | not applicable |
| **Clinical effectiveness** | ***Outcome(s) and measurement*** | Mortality: rate (based on 90-day overall survival) |
|  | ***Valuation*** | not applicable |
|  | ***Source*** | medical charts, hospital information system |
|  | ***Time horizon (months)*** | 3 |
|  | ***Discounting*** | not reported, but assumed no given the time frame of less than 1 year |
| **Costs** | ***Perspective*** | Mix of payer/ provider perspective |
|  | ***Type*** | direct |
|  | ***Calculation approach*** | bottom-up |
|  | ***Resource use measurement*** | Cost represented the amount of resources consumed by each patient (medications, supplies, salaries, operating room costs, and support costs).‡ "unit costing during hospital stay, mainly driven by the lenght of hospitalization" |
|  | ***Valuation*** | Costs in EUR |
|  | ***Source*** | Finance Department |
|  | ***Time horizon (months)*** | until discharge‡ |
|  | ***Reference year and currency*** | EURO (2020)‡ |
|  | ***Discounting*** | not reported, but assumed no given the time frame of less than 1 year |
|  | ***Correction for inflation*** | no‡; "no correction for inflation" |

Risco 2022 (51)

| **Type and design of analysis** | | CCA; trial-based (NRSI) |
| --- | --- | --- |
| **Further references** | | Baltaxe E, Cano I, Herranz C, Barberan-Garcia A, Hernandez C, Alonso A, et al. Evaluation of integrated care services in Catalonia: population-based and service-based real-life deployment protocols. BMC Health Services Research. 2019;19(1):370. |
| **Clinical effectiveness** | ***Outcome(s) and measurement*** | Morbidity: severity of postoperative complications (Comprehensive Complications Index score, 0 (best) to 100 (worst)) |
|  | ***Valuation*** | not applicable |
|  | ***Source*** | medical charts‡ |
|  | ***Time horizon (months)*** | 1 |
|  | ***Discounting*** | not reported, but assumed no given the time frame of less than 1 year |
| **Costs** | ***Perspective*** | provider perspective |
|  | ***Type*** | direct |
|  | ***Calculation approach*** | bottom-up and top-down |
|  | ***Resource use measurement*** | DRGs and micro costing |
|  | ***Valuation*** | Costs in EUR |
|  | ***Source*** | Hospital patient-level data |
|  | ***Time horizon (months)*** | 1 |
|  | ***Reference year and currency*** | EURO (2017-2019)‡ |
|  | ***Discounting*** | not reported, but assumed no given the time frame of less than 1 year |
|  | ***Correction for inflation*** | no‡: "With this kind of cost we considered, according to the usual methodology, that there is no need for correction for inflation." |

Tew 2017 (52)

| **Type and design of analysis** | | CEA; trial-based (RCT) |
| --- | --- | --- |
| **Further references** | | Tew GA, Weston M, Kothmann E, Batterham AM, Gray J, Kerr K, et al. High-intensity interval exercise training before abdominal aortic aneurysm repair (HIT-AAA): protocol for a randomised controlled feasibility trial. BMJ Open. 2014;4(1):e004094. |
| **Clinical effectiveness** | ***Outcome(s) and measurement*** | HrQoL: EQ-5D-5L (utility score, 0 (worst) to 1 (best)) |
|  | ***Valuation*** | Health state valuations based on UK value set (Dolan et al. 1997) |
|  | ***Source*** | trial documentation |
|  | ***Time horizon (months)*** | 3 |
|  | ***Discounting*** | not reported, but assumed no given the time frame of less than 1 year |
| **Costs** | ***Perspective*** | Mix of payer/ provider perspective (NHS and personal social services perspective) |
|  | ***Type*** | direct |
|  | ***Calculation approach*** | bottom-up |
|  | ***Resource use measurement*** | Cost of exercise programme (micro-costing for resources utilised in delivering the exercise programme), costs of AAA repair, Post-discharge costs (unit costs for the health and personal social services used by participants in the 12-week period after hospital discharge). Capital costs was annuitised over the useful lifespan of the equipment (assumed to be 3 years for the watches and heart rate belts and 8 years for the cycle ergometers). Post-discharge costs: Data regarding personal costs to each trial participant, including informal care-givers time, was not included owing to the unreliability of the data. |
|  | ***Valuation*** | Personal Social Services Research Unit’s of Health and Social Care, NHS and Reference costs |
|  | ***Source*** | study case report forms ((peri-)operative costs), patient diary (post-operative costs) |
|  | ***Time horizon (months)*** | 3 |
|  | ***Reference year and currency*** | GBP (2015-16) |
|  | ***Discounting*** | not reported, but assumed no given the time frame of less than 1 year |
|  | ***Correction for inflation*** | Yes‡ |
| **Analysis** | ***Description*** | For all clinical and patient-reported outcomes, point estimates and their uncertainty are presented as an indication of the range of effect sizes consistent with the data. No robust inference was attempted, as this was a feasibility study that was not powered to detect small yet clinically meaningful effects. |
|  | ***Model assumptions*** | not applicable |
|  | ***Assessment of uncertainty*** | bootstrapped mean differences between trial arms‡ |
|  | ***Handling of missing data*** | HrQoL: For the EQ-5DTM utility index, EQ-VAS, and SF-36® PF and MH subscales at week 5 and 12 weeks after discharge from hospital, a linear mixed model was used with restricted maximum likelihood, adjusted for baseline score, operative procedure and trial site. This model included all three time points in the same analysis, a principled method for handling any data missing at random on the dependent variable. |
|  | ***Willingness-to-pay threshold*** | None, as it was a feasibility study.‡ |

Tveter 2020 (53)

| **Type and design of analysis** | | CUA; trial-based (RCT) |
| --- | --- | --- |
| **Further references** | | Gravås EMH, Østerås N, Nossum R, Eide REM, Klokkeide Å, Matre KH, et al. Does occupational therapy delay or reduce the proportion of patients that receives thumb carpometacarpal joint surgery? A multicentre randomised controlled trial. RMD Open. 2019;5(2):e001046.  Kjeken I, Eide REM, Klokkeide Å, Matre KH, Olsen M, Mowinckel P, et al. Does occupational therapy reduce the need for surgery in carpometacarpal osteoarthritis? Protocol for a randomized controlled trial. BMC Musculoskeletal Disorders. 2016;17(1):473.  Kjeken I, Eide REM, Klokkeide Å, Matre KH, Olsen M, Mowinckel P, et al. Erratum to: Does occupational therapy reduce the need for surgery in carpometacarpal osteoarthritis? Protocol for a randomized controlled trial. BMC Musculoskeletal Disorders. 2017;18(1):68. |
| **Clinical effectiveness** | ***Outcome(s) and measurement*** | QALY: time spent in EQ-5D-5L health states* |
|  | ***Valuation*** | not reported* |
|  | ***Source*** | trial documentation |
|  | ***Time horizon (months)*** | 24 |
|  | ***Discounting*** | not reported* |
| **Costs** | ***Perspective*** | unclear* |
|  | ***Type*** | direct and indirect |
|  | ***Calculation approach*** | bottom-up |
|  | ***Resource use measurement*** | Form protocol: […] the costs will include the cost of the two interventions. This comprises the hours and frequency of occupational therapy, costs related to the provision of assistive devices, orthoses, and exercise material, and costs related to the surgical procedures, including post-operative treatment. Furthermore, costs related to medical or technical equipment purchased by participants and to the use of other health care services (home care services, rehabilitation, and institution) will be recorded for both the intervention and control group during the trial period. Further, the direct and indirect costs in the study period will be self-reported at 4, 18 and 24 months as the number of days of sick leave and absence from paid work, number of visits to a given list of health providers and the number of hospital visits or stays over the period since previous control (see table 5). |
|  | ***Valuation*** | not reported* |
|  | ***Source*** | details unclear*: questionnaires, patient diary |
|  | ***Time horizon (months)*** | 24 |
|  | ***Reference year and currency*** | EURO (year not reported)* |
|  | ***Discounting*** | not reported* |
|  | ***Correction for inflation*** | not reported* |
| **Analysis** | ***Description*** | Standard methods for economic evaluation will be applied and the cost-effectiveness will be calculated as the incremental cost-effectiveness ratio, which is defined by the cost per incremental QALY. Sensitivity analyses were performed to assess the robustness of the reported Results. The results are presented in a cost-effectiveness plane using bootstrapping. |
|  | ***Model assumptions*** | not applicable |
|  | ***Assessment of uncertainty*** | bootstrap resampling method with 1,000 replications of the study data |
|  | ***Handling of missing data*** | not reported* |
|  | ***Willingness-to-pay threshold*** | 27.500 € |

Van Wijk 2020 (54)

| **Type and design of analysis** | | CBA; model-based (decision tree) |
| --- | --- | --- |
| **Further references** | | not applicable |
| **Clinical effectiveness** | ***Outcome(s) and measurement*** | Morbidity: rate of postoperative complications (not specified) |
|  | ***Valuation*** | costs |
|  | ***Source*** | Dutch Institute for Clinical Auditing, literature |
|  | ***Time horizon (months)*** | until discharge |
|  | ***Discounting*** | not reported† |
| **Costs** | ***Perspective*** | not reported† |
|  | ***Type*** | direct |
|  | ***Calculation approach*** | top-down |
|  | ***Resource use measurement*** | costs |
|  | ***Valuation*** | costs in EUR |
|  | ***Source*** | Dutch Institute for Clinical Auditing, literature |
|  | ***Time horizon (months)*** | until discharge |
|  | ***Reference year and currency*** | EUR (year not reported)† |
|  | ***Discounting*** | not reported† |
|  | ***Correction for inflation*** | not reported† |
| **Analysis** | ***Description*** | The cost-effectiveness was determined with four main statistics: the incidence of the patient related risk factor, the average costs of the intervention to optimize the patient with respect to the risk factor, the success rate of the intervention and the weighted importance of the intervention that contributes to risk reduction based on a Delphi method. These four main statistics and the current data on complication rates, the expected maximal increase and decrease in complication rate related to the presence or absence of all risk factors, and the hospital admission costs were brought together into a scenario tree. |
|  | ***Model assumptions*** | Pancreatic surgery was used as an example to fill in the model and calculate the potential financial benefits when a multimodal prehabilitation program would be implemented. |
|  | ***Assessment of uncertainty*** | none stated |
|  | ***Handling of missing data*** | not applicable |
|  | ***Willingness-to-pay threshold*** | none stated |

Wang 2020 (55)

| **Type and design of analysis** | | CCA; trial-based (NRSI) |
| --- | --- | --- |
| **Further references** | | not applicable |
| **Clinical effectiveness** | ***Outcome(s) and measurement*** | Morbidity: rate of postoperative complications (all types) Mortality: rate PROMs: Functional capacity (Functional Assessment of Cancer Therapy-Hepatobiliary (FACT-Hep) score, 0 (worst) to 180 (best)) |
|  | ***Valuation*** | not applicable |
|  | ***Source*** | medical charts, trial documentation |
|  | ***Time horizon (months)*** | Morbidity and PROMs: unclear*; assumed until discharge, mortality: 3 |
|  | ***Discounting*** | not reported, but assumed no given the time frame of less than 1 year |
| **Costs** | ***Perspective*** | unclear* |
|  | ***Type*** | direct |
|  | ***Calculation approach*** | bottom-up |
|  | ***Resource use measurement*** | Details unclear*; “The payment for PP was bundled together with patients’ preoperative assessment charges; inpatient costs (initial admission/readmission)” |
|  | ***Valuation*** | actual charges |
|  | ***Source*** | hospital payment system |
|  | ***Time horizon (months)*** | unclear of 1 or only until discharge* |
|  | ***Reference year and currency*** | not reported*: assumed Singapore dollar |
|  | ***Discounting*** | not reported, but assumed no given the time frame of less than 1 year |
|  | ***Correction for inflation*** | not reported* |

Zhou 2017 (34)

| **Type and design of analysis** | | CCA; trial-based (NRSI) |
| --- | --- | --- |
| **Further references** | | not applicable |
| **Clinical effectiveness** | ***Outcome(s) and measurement*** | Morbidity: rate of postoperative pulmonary complications (pneumonia, prolonged air leak (≥7 days), atelectasis, pleural effusion requiring drainage, acute respiratory distress syndrome, respiratory failure, mechanical ventilation ≥48 hours postoperatively, pulmonary embolism, empyema; classified as per Clavien-Dindo and only including grade II–V in final analysis) |
|  | ***Valuation*** | not applicable |
|  | ***Source*** | hospital database |
|  | ***Time horizon (months)*** | 1 |
|  | ***Discounting*** | not reported, but assumed no given the time frame of less than 1 year |
| **Costs** | ***Perspective*** | unclear* |
|  | ***Type*** | direct |
|  | ***Calculation approach*** | bottom-up |
|  | ***Resource use measurement*** | Details unclear*; “Preoperative costs (ward expense, care expense, rehabilitation expense) + surgery related expense + postoperative costs (drug expense, ward expense, care expense)” |
|  | ***Valuation*** | actual charges |
|  | ***Source*** | Details unclear*; “hospital database” |
|  | ***Time horizon (months)*** | until discharge |
|  | ***Reference year and currency*** | not reported* |
|  | ***Discounting*** | not reported, but assumed no given the time frame of less than 1 year |
|  | ***Correction for inflation*** | not reported* |

### Appendix 8 – Methods of ongoing economic evaluations with a published protocol

Barberan-Garcia 2020 (56)

| **Type and design of analysis** | | not reported; trial-based (RCT) |
| --- | --- | --- |
| **Clinical effectiveness** | ***Outcome(s) and measurement*** | HrQoL: Self-perceived health status (Short Form 36, 0 (worst) to 100 (best)) Morbidity: rate of postoperative complications (classified as per Clavien-Dindo) PROMs: 1) Physical activity (Yale Physical Activity Survey, 0 (worst) to X (best)) 2) Psychological status (Hospital anxiety and depression questionnaire, 0 (best) to 21 (worst)) Physical function: aerobic capacity (standard cardiopulmonary exercise test) |
|  | ***Valuation*** | not applicable |
|  | ***Source*** | not reported |
|  | ***Time horizon (months)*** | 1 |
|  | ***Discounting*** | not reported |
| **Costs** | ***Perspective*** | provider perspective |
|  | ***Type*** | direct |
|  | ***Calculation approach*** | bottom-up |
|  | ***Resource use measurement*** | i) Patient-chargeable costs (i.e. pharmacy and blood bank); ii) Tariff-chargeable costs (i.e. medical care, diagnostic techniques, laboratories, specialist consultations, hospital length of stay and hostelry); and, iii) Other costs (i.e. support and structural costs) |
|  | ***Valuation*** | not reported |
|  | ***Source*** | not reported |
|  | ***Time horizon (months)*** | 1 |
|  | ***Reference year and currency*** | not reported |
|  | ***Discounting*** | not reported |
|  | ***Correction for inflation*** | not reported |
| **Analysis** | ***Description*** | The effect of the intervention will be studied by intention to treat through regression analysis (linear, logistic, Cox or Poisson, depending on the distribution of the variable), including the exposure to the intervention as the main variable and as co-variables those in which the intervention group and the control group are different at baseline, if there were any present. |
|  | ***Model assumptions*** | not applicable |
|  | ***Assessment of uncertainty*** | not reported |
|  | ***Handling of missing data*** | not reported |
|  | ***Willingness-to-pay threshold*** | none stated |

Coca-Martinez 2020 (57)

| **Type and design of analysis** | | not reported; trial-based (RCT) |
| --- | --- | --- |
| **Clinical effectiveness** | ***Outcome(s) and measurement*** | Morbidity: rate of postoperative complications (classified as per European Society of Anaesthesiology and European Society of intensive Care Medicine; classified as per Dindo-Clavien) Mortality: rate PROMs:  1) Physical activity (Yale Physical Activity Survey, 0 (worst) to X (best)) 2) Psychological status (Hospital anxiety and depression questionnaire, 0 (best) to 21 (worst)) Physical function: 1) aerobic capacity (constant work rate at 80% of peak oxygen uptake) 2) aerobic capacity and endurance (6 minutes walk test) 3) leg strength and mobility (sit-to-stand test) 4) hand-grip strength |
|  | ***Valuation*** | not applicable |
|  | ***Source*** | trial documentation |
|  | ***Time horizon (months)*** | 3 (additionally at 12 months for mortality) |
|  | ***Discounting*** | not reported |
| **Costs** | ***Perspective*** | provider perspective |
|  | ***Type*** | direct |
|  | ***Calculation approach*** | not reported |
|  | ***Resource use measurement*** | costs of the intervention and the expenses related to the disease |
|  | ***Valuation*** | not reported |
|  | ***Source*** | not reported |
|  | ***Time horizon (months)*** | 12 |
|  | ***Reference year and currency*** | not reported |
|  | ***Discounting*** | not reported |
|  | ***Correction for inflation*** | not reported |
| **Analysis** | ***Description*** | Categorical variables, as well primary outcome, will be analysed with Fisher’s exact test. Continuous variables will be compared with Student's t-test for independent groups and Mann-Whitney U test according to each variable distribution. Ordinal variables will be analysed by Mann-Whitney U test. Secondary outcomes looking at the difference between groups for the change in functional capacity over the different time points (repeated measures) will be analysed using generalised estimating equations models and will be shown as estimated effect and their 95%CI. For these analyses, we will apply unstructured matrix in order to assess intra-subject correlation, for cases with no adjustments we will apply an autoregressive model (AR) (1) type matrix. |
|  | ***Model assumptions*** | not applicable |
|  | ***Assessment of uncertainty*** | Considering previous experience with prehabilitation cost analysis,19 a highly skewed distribution will probably be present. Right-sided asymmetric distribution appears when some patients incur in high costs, mainly because of major medical complications. To deal with this, a non-parametric approach (bootstrapping) will be used. Bootstrap analysis yields more robust when dealing with skewed cost data compared with non-parametric tests. |
|  | ***Handling of missing data*** | not reported |
|  | ***Willingness-to-pay threshold*** | none stated |

Pufulete 2020 (58)

| **Type and design of analysis** | | CUA; trial-based (RCT) |
| --- | --- | --- |
| **Clinical effectiveness** | ***Outcome(s) and measurement*** | QALY: time spent in EQ-5D-5L health states |
|  | ***Valuation*** | Health state valuations from the UK general population |
|  | ***Source*** | trial documentation |
|  | ***Time horizon (months)*** | 6 |
|  | ***Discounting*** | not reported |
| **Costs** | ***Perspective*** | Mix of provider and payer perspective (NHS and personal social services perspective) |
|  | ***Type*** | direct |
|  | ***Calculation approach*** | bottom-up |
|  | ***Resource use measurement*** | Resource use data will be collected using the trial case report forms and patient questionnaires (at 3 months and 6 months post-surgery), and will cover length of stay in hospital (including any readmissions), time in intensive care, treating any complications, and further contact with health professionals in primary or secondary care. Unit costs will be derived from nationally published sources such as the NHS Reference Costs database (National Schedule of Reference Costs) [67] and hospital trust finances and attached to the resource use data. The costs of drugs given in hospital (including antibiotics) will be taken from the Electronic Marketing Information Tool where possible, which provides the reduced prices paid for generic drugs in hospital [68]. Drug costs not available from this source will be taken from the British National Formulary (BNF) [69]. |
|  | ***Valuation*** | not reported |
|  | ***Source*** | Resource use data will be collected using the trial case report forms and patient questionnaires |
|  | ***Time horizon (months)*** | 6 |
|  | ***Reference year and currency*** | not reported |
|  | ***Discounting*** | not reported |
|  | ***Correction for inflation*** | not reported |
| **Analysis** | ***Description*** | From the average costs and QALYs gained in each study group, the incremental cost-effectiveness ratio (ICER) will be derived, producing an incremental cost per QALY gained of IMT compared to no IMT. [...] Results will be expressed in terms of a cost-effectiveness acceptability curve, which indicates the likelihood that IMT is cost-effective for different levels of willingness to pay for health gain. |
|  | ***Model assumptions*** | not applicable |
|  | ***Assessment of uncertainty*** | Uncertainty around the ICER will be represented graphically on the cost-effectiveness plane by the bootstrap replicates of the mean difference in costs and QALYs between the groups. |
|  | ***Handling of missing data*** | Missing resource use and EQ-5D data will be handled using multiple imputation methods |
|  | ***Willingness-to-pay threshold*** | £20,000 per QALY |

Schaller 2022 (59)

| **Type and design of analysis** | | I) CUA, II) CEAs; trial-based (RCT) |
| --- | --- | --- |
| **Clinical effectiveness** | ***Outcome(s) and measurement*** | QALY: time spent in EQ-5D-5L health states |
|  | ***Valuation*** | Appropriate utility weights |
|  | ***Source*** | trial documentation |
|  | ***Time horizon (months)*** | 12 |
|  | ***Discounting*** | not reported |
| **Costs** | ***Perspective*** | I) Societal perspective II) Payer perspective |
|  | ***Type*** | direct and indirect |
|  | ***Calculation approach*** | bottom-up and top-down |
|  | ***Resource use measurement*** | All direct medical and non-medical healthcare-related resource utilization will be monitored using a validated questionnaire for health-related resource use by elderly patients |
|  | ***Valuation*** | Costs in EUR |
|  | ***Source*** | Questionnaire |
|  | ***Time horizon (months)*** | 12 |
|  | ***Reference year and currency*** | EUR (year not reported) |
|  | ***Discounting*** | not reported |
|  | ***Correction for inflation*** | not reported |
| **Analysis** | ***Description*** | not reported |
|  | ***Model assumptions*** | not applicable |
|  | ***Assessment of uncertainty*** | Probabilistic and deterministic sensitivity analyses will be performed to explore any uncertainty in the results. |
|  | ***Handling of missing data*** | not reported |
|  | ***Willingness-to-pay threshold*** | different willingness to pay assumptions |

Sheill 2020 (60)

Please note: An updated protocol reflecting changes due to the Corona Virus 2019 (COVID-19) pandemic was published on an Open Access Publishing Platform in 2021: Sheill G, Guinan E, O'Neill L, Smyth E, Normand C, L. Doyle S, et al. Prehabilitation during a pandemic: preoperative exercise to improve fitness in patients undergoing complex surgery for cancer of the lung or oesophagus, the PRE-HIIT trial: an updated study protocol [version 1; peer review: 1 approved, 1 approved with reservations]. HRB Open Research. 2021;4(4).

| **Type and design of analysis** | | CEA; trial-based (RCT) |
| --- | --- | --- |
| **Clinical effectiveness** | ***Outcome(s) and measurement*** | HrQoL: EQ-5D-5L (score; not specified), European Organization for Research and Treatment of Cancer Core Quality of Life Questionnaire (EORTC QLQ-C30) (score; not specified) |
|  | ***Valuation*** | not applicable |
|  | ***Source*** | trial documentation |
|  | ***Time horizon (months)*** | 3 |
|  | ***Discounting*** | not reported |
| **Costs** | ***Perspective*** | not reported |
|  | ***Type*** | direct |
|  | ***Calculation approach*** | bottom-up and top-down |
|  | ***Resource use measurement*** | The costing of hospital stays, and interventions will be carried out based on activity data from hospital records, with unit costs taken from the standard estimated costs from the Healthcare Pricing Office. Programme implementation costs will be analysed in consideration of clinician salaries, overheads and equipment costs. Formal care costs will be extracted from medical charts and from the institutional database in consideration of pre-operative characteristics, surgery type and postoperative recovery including complications.  The destination at discharge, use of community health services from the time of discharge to the follow up outpatient appointment and EQ5D5L scores at the time of the follow up appointment will be collected for each participant. Tool: Service Use Inventory |
|  | ***Valuation*** | not reported |
|  | ***Source*** | hospital records, Healthcare Pricing Office, institutional database, trial documentation |
|  | ***Time horizon (months)*** | 3 |
|  | ***Reference year and currency*** | not reported |
|  | ***Discounting*** | not reported |
|  | ***Correction for inflation*** | not reported |
| **Analysis** | ***Description*** | A linear mixed model will be used to model the longitudinal change in the primary response between the groups, allowing for missing data (under the assumption that data are missing at random) and allowing for within subject correlations in the repeated measures across time. The model will adjust for the baseline response variable and other covariates as necessary. |
|  | ***Model assumptions*** | not applicable |
|  | ***Assessment of uncertainty*** | not reported |
|  | ***Handling of missing data*** | A linear mixed model will be used to model the longitudinal change in the primary response between the groups, allowing for missing data (under the assumption that data are missing at random) and allowing for within subject correlations in the repeated measures across time. |
|  | ***Willingness-to-pay threshold*** | none stated |

Stamp 2021 (61)

| **Type and design of analysis** | | not reported; trial-based (RCT) |
| --- | --- | --- |
| **Clinical effectiveness** | ***Outcome(s) and measurement*** | not reported |
|  | ***Valuation*** | not reported |
|  | ***Source*** | not reported |
|  | ***Time horizon (months)*** | not reported |
|  | ***Discounting*** | not reported |
| **Costs** | ***Perspective*** | not reported |
|  | ***Type*** | direct |
|  | ***Calculation approach*** | not reported |
|  | ***Resource use measurement*** | cost of stay |
|  | ***Valuation*** | not reported |
|  | ***Source*** | not reported |
|  | ***Time horizon (months)*** | until discharge |
|  | ***Reference year and currency*** | not reported |
|  | ***Discounting*** | not reported |
|  | ***Correction for inflation*** | not reported |
| **Analysis** | ***Description*** | not reported |
|  | ***Model assumptions*** | not applicable |
|  | ***Assessment of uncertainty*** | not reported |
|  | ***Handling of missing data*** | not reported |
|  | ***Willingness-to-pay threshold*** | not reported |

Steffens 2022 (62)

| **Type and design of analysis** | | I) CEA, II) CUA; trial-based (RCT) |
| --- | --- | --- |
| **Clinical effectiveness** | ***Outcome(s) and measurement*** | I) Morbidity: rate of postoperative complications (defined as per the Standardised Endpoints Core Outcome Measures in Perioperative and Anaesthetic Care (StEP-COMPAQ) group) II) QALY: time spent in Short Form 36 health states |
|  | ***Valuation*** | Health state valuations based on UK value set (Bazier et al. 2002) |
|  | ***Source*** | trial documentation |
|  | ***Time horizon (months)*** | Morbidity: until discharge QALY: 3 |
|  | ***Discounting*** | not reported |
| **Costs** | ***Perspective*** | not reported |
|  | ***Type*** | direct |
|  | ***Calculation approach*** | bottom-up |
|  | ***Resource use measurement*** | direct costs to the healthcare system and out-of-pocket costs to patients |
|  | ***Valuation*** | not reported |
|  | ***Source*** | case report forms and monthly patient diaries |
|  | ***Time horizon (months)*** | 3 |
|  | ***Reference year and currency*** | not reported |
|  | ***Discounting*** | not reported |
|  | ***Correction for inflation*** | not reported |
| **Analysis** | ***Description*** | Both costs and outcomes will be reported separately for each group (following best practice methods) [55] and an incremental cost-effectiveness ratio (ICER) will be calculated for (i) the additional cost of a pre-operative exercise program per in-hospital complication avoided; and (ii) the additional cost per QALY gained at 3 months. |
|  | ***Model assumptions*** | not applicable |
|  | ***Assessment of uncertainty*** | not reported |
|  | ***Handling of missing data*** | multiple imputations will be performed |
|  | ***Willingness-to-pay threshold*** | none stated |

Svinøy 2019 (63)

| **Type and design of analysis** | | CUA; trial-based (RCT) |
| --- | --- | --- |
| **Clinical effectiveness** | ***Outcome(s) and measurement*** | QALY: time spent in EQ-5D health states |
|  | ***Valuation*** | not reported |
|  | ***Source*** | trial documentation |
|  | ***Time horizon (months)*** | 3 |
|  | ***Discounting*** | not reported |
| **Costs** | ***Perspective*** | Mix of provider and patient perspective |
|  | ***Type*** | direct and indirect |
|  | ***Calculation approach*** | bottom-up |
|  | ***Resource use measurement*** | Some of the relevant types of cost included in the CUA are the actual time used for training (both the physical therapists and participants), travel expenses, hospitalisation, medicine, home help service, informal care and other health services. |
|  | ***Valuation*** | The unit costs will be based on marked prices, the reimbursement systems in Norway and literature. |
|  | ***Source*** | Information on costs will be obtained by using the validated instrument Client Service Receipt Inventory covering costs of health, social and informal services. The treatment cost will be calculated and included. |
|  | ***Time horizon (months)*** | 3 |
|  | ***Reference year and currency*** | not reported |
|  | ***Discounting*** | not reported |
|  | ***Correction for inflation*** | not reported |
| **Analysis** | ***Description*** | We will aim to perform sensitivity analysis for changes in unit cost and uncertainty related to generalisability and extrapolation. |
|  | ***Model assumptions*** | not applicable |
|  | ***Assessment of uncertainty*** | not reported |
|  | ***Handling of missing data*** | If data are missing at random, the linear mixed model for repeated measurements is considered robust. In addition, we will use multiple imputation to assess its robustness for missing data at random. If data are assumed to be not missed by random, we will apply Bayesian modelling. If the data are missing completely at random, the primary statistical methods are robust. |
|  | ***Willingness-to-pay threshold*** | none stated |

van Rooijen 2019 (64)

| **Type and design of analysis** | | I) CEA, II) CUA; trial-based (RCT) |
| --- | --- | --- |
| **Clinical effectiveness** | ***Outcome(s) and measurement*** | I) Morbidity: severity of postoperative complications (Comprehensive Complications Index score, 0 (best) to 100 (worst)) Mortality: rate PROMs: 1) anxiety and depression (Generalized Anxiety Disorder Scale-7 (GAD-7), 0 (best) to 21 (worst)) 2) anxiety and depression (Patient Health Questionnaire-9 (PHQ-9), 0 (best) to 27 (worst)) II) QALY: time spent in Short Form 36 health states |
|  | ***Valuation*** | I) not applicable  II) not reported |
|  | ***Source*** | trial documentation |
|  | ***Time horizon (months)*** | Morbidity: 1, All other outcomes: 12 |
|  | ***Discounting*** | not reported |
| **Costs** | ***Perspective*** | societal perspective |
|  | ***Type*** | direct and indirect |
|  | ***Calculation approach*** | bottom-up |
|  | ***Resource use measurement*** | reduction of complications, improving survival, less need for postoperative care and improvements in social productivity |
|  | ***Valuation*** | not reported |
|  | ***Source*** | iMTA-PCQ questionnaires |
|  | ***Time horizon (months)*** | 12 |
|  | ***Reference year and currency*** | not reported |
|  | ***Discounting*** | not reported |
|  | ***Correction for inflation*** | not reported |
| **Analysis** | ***Description*** | All secondary outcomes will be described as means plus SD or median plus IQR, with the data being continuous, and measures for each time point being normally and non-normally distributed respectively. Categorical parameters will be described as number plus percentage per time point. Statistical methods will include t-test and the Mann-Whitney U test for continuous parameters, distributed either normally or not normally respectively, at a single postoperative time-point. Categorical outcomes will be analyzed with Chi-square testing or regression analysis (logistic, ordinal or nominal, depending on the definition of the parameter) for single time points. |
|  | ***Model assumptions*** | not applicable |
|  | ***Assessment of uncertainty*** | not reported |
|  | ***Handling of missing data*** | not reported |
|  | ***Willingness-to-pay threshold*** | none stated |

West 2021 (65)

| **Type and design of analysis** | | CUA; trial-based (RCT) |
| --- | --- | --- |
| **Clinical effectiveness** | ***Outcome(s) and measurement*** | QALY: time spent in EQ-5D-5L health states |
|  | ***Valuation*** | not reported |
|  | ***Source*** | trial documentation |
|  | ***Time horizon (months)*** | 12 |
|  | ***Discounting*** | not reported |
| **Costs** | ***Perspective*** | not reported |
|  | ***Type*** | not reported |
|  | ***Calculation approach*** | not reported |
|  | ***Resource use measurement*** | not reported |
|  | ***Valuation*** | not reported |
|  | ***Source*** | not reported |
|  | ***Time horizon (months)*** | not reported |
|  | ***Reference year and currency*** | not reported |
|  | ***Discounting*** | not reported |
|  | ***Correction for inflation*** | not reported |
| **Analysis** | ***Description*** | not reported |
|  | ***Model assumptions*** | not applicable |
|  | ***Assessment of uncertainty*** | not reported |
|  | ***Handling of missing data*** | not reported |
|  | ***Willingness-to-pay threshold*** | none stated |

Yau 2019 (66)

| **Type and design of analysis** | | CUA; trial-based (RCT) |
| --- | --- | --- |
| **Clinical effectiveness** | ***Outcome(s) and measurement*** | QALY: time spent in EQ-5D health states |
|  | ***Valuation*** | Health state valuations based on Hong Kong reference weights |
|  | ***Source*** | trial documentation |
|  | ***Time horizon (months)*** | 3 |
|  | ***Discounting*** | not reported |
| **Costs** | ***Perspective*** | provider perspective |
|  | ***Type*** | direct |
|  | ***Calculation approach*** | not reported |
|  | ***Resource use measurement*** | outpatient prehabilitation sessions, ICU and hospital stays, postoperative outpatient visits and readmissions within 3 months. |
|  | ***Valuation*** | The physiotherapist staff salary, obtained from the personnel department, will be based on the midpoint of the relevant pay scale. Prices will be based on 2020 costs in Hong Kong dollars. |
|  | ***Source*** | not reported |
|  | ***Time horizon (months)*** | 3 |
|  | ***Reference year and currency*** | Hong Kong dollars (2020) |
|  | ***Discounting*** | not reported |
|  | ***Correction for inflation*** | not reported |
| **Analysis** | ***Description*** | [A] cost-effectiveness acceptability curve will be drawn to detect differences in the joint cost-effects (DAH30 or QALY) relationships between groups using the net-benefit framework.44 Prehabilitation will be considered as cost-effective if there is a reduction in the overall perioperative treatment cost per gain in DAH30 and/or QALYs. |
|  | ***Model assumptions*** | not applicable |
|  | ***Assessment of uncertainty*** | not reported |
|  | ***Handling of missing data*** | Missing data will be checked and imputed using the most common category value for categorical variables or median for continuous variables if there is less than 10% missing data. Otherwise, multiple imputation techniques will be used. |
|  | ***Willingness-to-pay threshold*** | none stated |

### Appendix 9 – Description of prehabilitation programmes in ongoing studies

| **Study ID, main reference** | **Type and modalities** | **Involved health care professionals** | **Setting** | **Overall duration, frequency and duration per session** |
| --- | --- | --- | --- | --- |
| Ongoing studies with published protocol | | | | |
| Barberan-Garcia 2020 (56) | Multimodal: exercise (endurance, strength), promotion of physical activity, nutrition (counselling, supplements), alcohol cessation, smoking cessation, psychosocial (stress management), counselling/education, | specialized physiotherapist, registered dietician, clinical health psychologist | outpatient - community | Overall duration not reported, 3x/week, for 47 min + strength |
| Coca-Martinez 2020 (57) | Multimodal: exercise (endurance, strength, inspiratory muscles), promotion of physical activity, nutrition (counselling, supplements), psychosocial (stress management) | physiotherapist, registered dietitian, registered psychologist | outpatient - hospital (endurance & strength training), home (breathing exercises) | 4-6 weeks, 2+1/week, 1-2/day (breathing exercise), for 60 min |
| Pufulete 2020 (58) | IG 1 and 2: Unimodal: exercise (inspiratory muscles) | research nurse, physiotherapist | home | minimum 2 weeks, 2x/day, 30 breaths |
| Sheill 2020 (60) | Multimodal: exercise (endurance), nutrition (supplements) | physiotherapist, registered dietitian | Originally planned: outpatient - hospital; Update: home | min 2 weeks, up to 5x/week, for 40 min |
| Schaller 2022 (59) | Multimodal: exercise (endurance, resistance, proprioception and balance), nutrition (not specified), psychosocial (not specified) | physiotherapist or occupational therapist | inpatient, outpatient - hospital, outpatient - community, home | 3 weeks, 5x/week, for 30 min |
| Stamp 2021 (61) | Unclear*; assumed unimodal: promotion of physical activity | cardiac surgery, anaesthesia, intensive care, exercise physiology, psychology and nursing | not reported | not reported |
| Steffens 2022 (62) | Multimodal: exercise (endurance, resistance, inspiratory muscles), promotion of physical activity, counselling/education | physiotherapists or exercise physiologists | outpatient - community | 4-8 weeks, 3x/week, for 50 min |
| Svinøy 2019 (63) | Multimodal: counselling/education, exercise (endurance, resistance, proprioception and balance) | physiotherapist | outpatient - hospital, possibly home | 6-12 weeks, 2x/week, for 45-60 min |
| van Rooijen 2019 (64) | Multimodal: exercise (endurance, resistance), counselling/education, nutrition (counselling, supplements), smoking cessation, psychosocial (anxiety reduction, stress management) | exercise specialist (kinesiologist, sport physician), dietician, psychologist | outpatient - hospital, home | 4 weeks, 3x/week, for 28-32 min |
| West 2021 (65) | Multimodal:  IG 1: exercise (endurance), counselling/education, psychosocial (anxiety reduction, other)  IG 2: exercise (endurance), counselling/education | personal trainers, psychological counsellors | outpatient - community, hospital | Overall duration not reported, 2-3x/week, for 30-40 min |
| Yau 2019 (66) | Multimodal: exercise (endurance, resistance, inspiratory muscles), counselling/education, nutrition (counselling), smoking cessation, psychosocial (other) | physiotherapist | outpatient - hospital, home | 6-10 weeks, 2x/week, for 40-80 min |
| Ongoing studies without published protocol | | | | |
| Albaladejo 2019 (75) | Multimodal: promotion of physical activity, nutrition (counselling, supplements), psychosocial (stress management) | not reported | not reported | not reported |
| Diaz-Feijoo 2021 (67) | Multimodal: exercise (endurance), promotion of physical activity, nutrition (counselling, supplements), psychosocial (stress management) | not reported | not reported | minimum 3 weeks, frequency and duration not reported |
| Hu 2021 (68) | Multimodal: exercise (endurance, resistance), nutrition (supplements), psychosocial (other) | not reported | not reported | Overall duration not reported, 3x/day, duration not reported |
| Lönnroos 2019 (69) | Multimodal: stabilizing diseases, nutrition (not specified), drug evaluation, promotion of physical activity | geriatrician, registered nurse, physiotherapist and pharmacist | not reported | not reported |
| McIsaac 2020 (71) | Unimodal: exercise (resistance, endurance, stretching and flexibility) | not reported | home | 3 weeks, 3-7x/week, for 60 min |
| Molenaar 2019 (70) | Multimodal: exercise (endurance, resistance, inspiratory muscles), nutrition (counselling, supplements), psychosocial (other, stress management), smoking cessation, counselling/education | not reported | not reported | 3 weeks, frequency and duration not reported |
| Ocampos 2019 (74) | Multimodal: counselling/education, nutrition (counselling), psychosocial (not specified), exercise (not specified) | psychologists, physical therapists, physical educators | outpatient - hospital | 6 months, frequency and duration not reported |
| Santa Mina 2019 (72) | Multimodal: exercise (endurance, resistance, disease-specific), nutrition (counselling, supplements), psychosocial (stress management) | registered kinesiologist/exercise physiologist | IG 1: outpatient  IG 2: home | Overall duration not reported, 3-5x/week, for 60 min |
| Strijker 2020 (73) | Multimodal: exercise (not specified), nutrition (not specified), psychosocial (not specified), smoking cessation | not reported | not reported | 4-8 weeks, frequency and duration not reported |
| * Authors did not respond. Abbreviations: IG, intervention group | | | | |

### Appendix 10 – Risk of bias and methodological quality of included economic evaluations


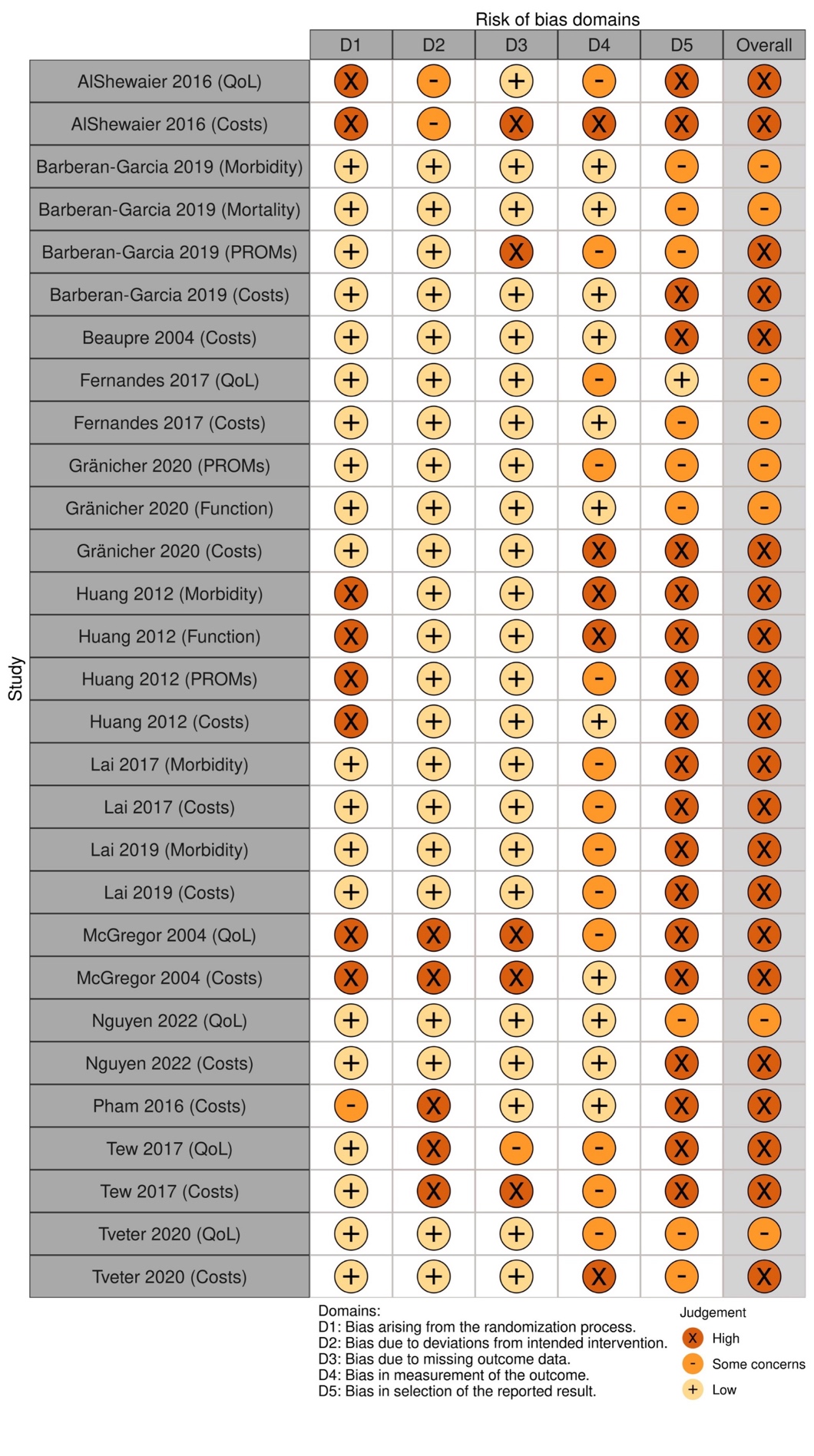


Figure 1: Risk of bias in RCT-based economic evaluations as judged with the Cochrane Risk of Bias 2 tool

NB: We deviated from the RoB 2 tool guidance with regards to item 5: When an RCT did not have a prospective registration record or protocol, we assigned a high risk of bias instead of “some concerns” because the 2008 revision the Declaration of Helsinki requires each trial involving human participants to be prospectively registered (see Krleza-Jerić and Lemmens 2009. Doi: 10.3325/cmj.2009.50.105). We applied this criterion also to two trials published before 2008 that were not registered (39, 47).

Furthermore, a high overall risk of bias was assumed for the outcome “costs” when the intervention costs (i.e., the costs for the prehabilitation programme) were not reported.


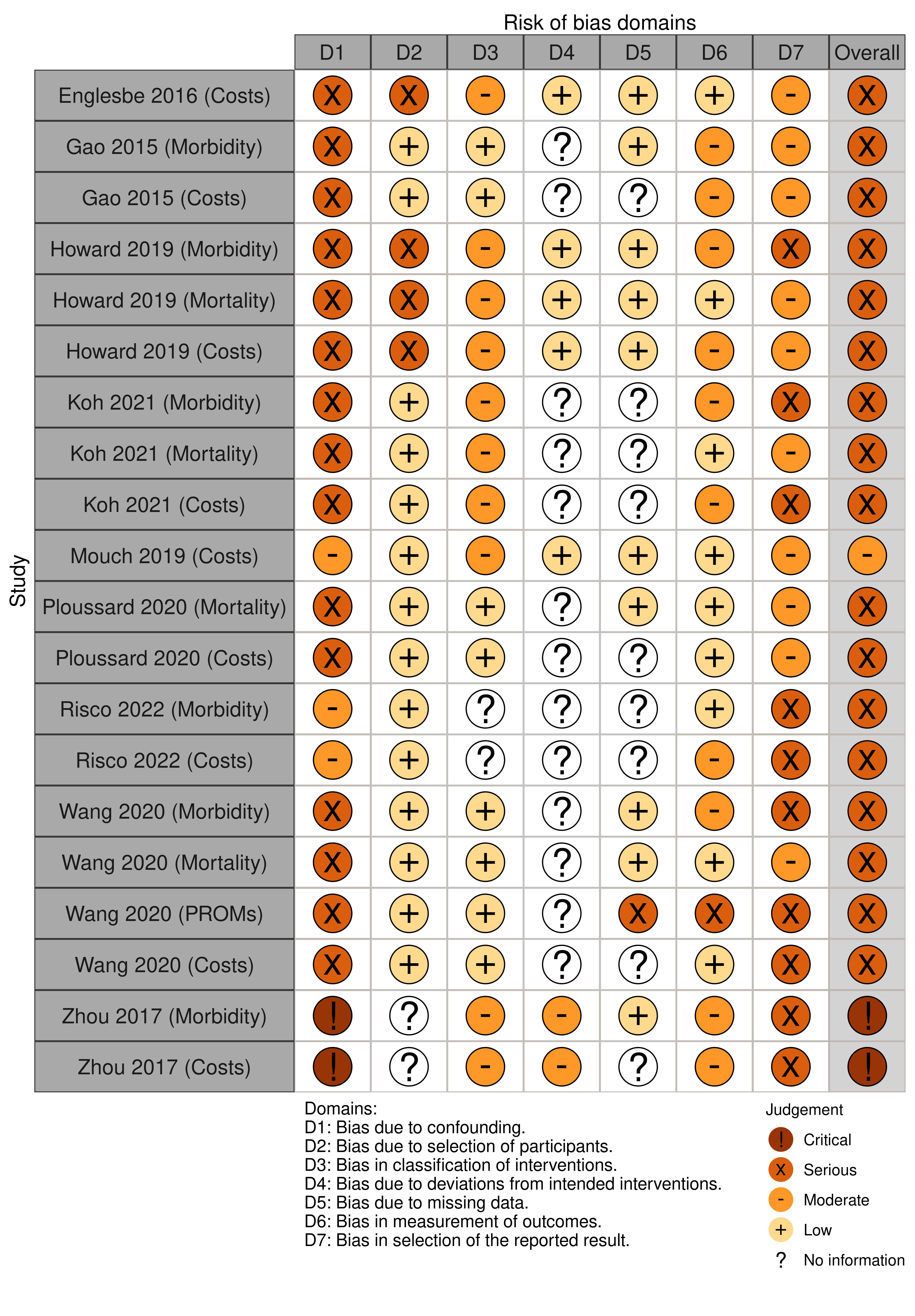


Figure 2: Risk of bias in NRSI-based economic evaluations as judged with the ROBINS-I tool

NB: The ROBINS-I tool is mainly intended for concurrently controlled studies NRSI (25) (see https://www.riskofbias.info/welcome/home/current-version-of-robins-i/robins-i-detailed-guidance-2016), but 3 EEs presented before-and-after studies (29, 46, 50). Thus, we added two extra items to the domain “Bias due to confounding”: Item 1.9. “In non-concurrently controlled studies (i.e., studies where cohorts come from different time periods), is it likely that there were extraneous events or changes in context around the time of the intervention that could have influenced the outcome?" and Item 1.10. “If Y/PY to 1.9. Did the authors use an appropriate analysis method that accounts for relevant time trends and patterns, and controls for all the important confounding domains?".


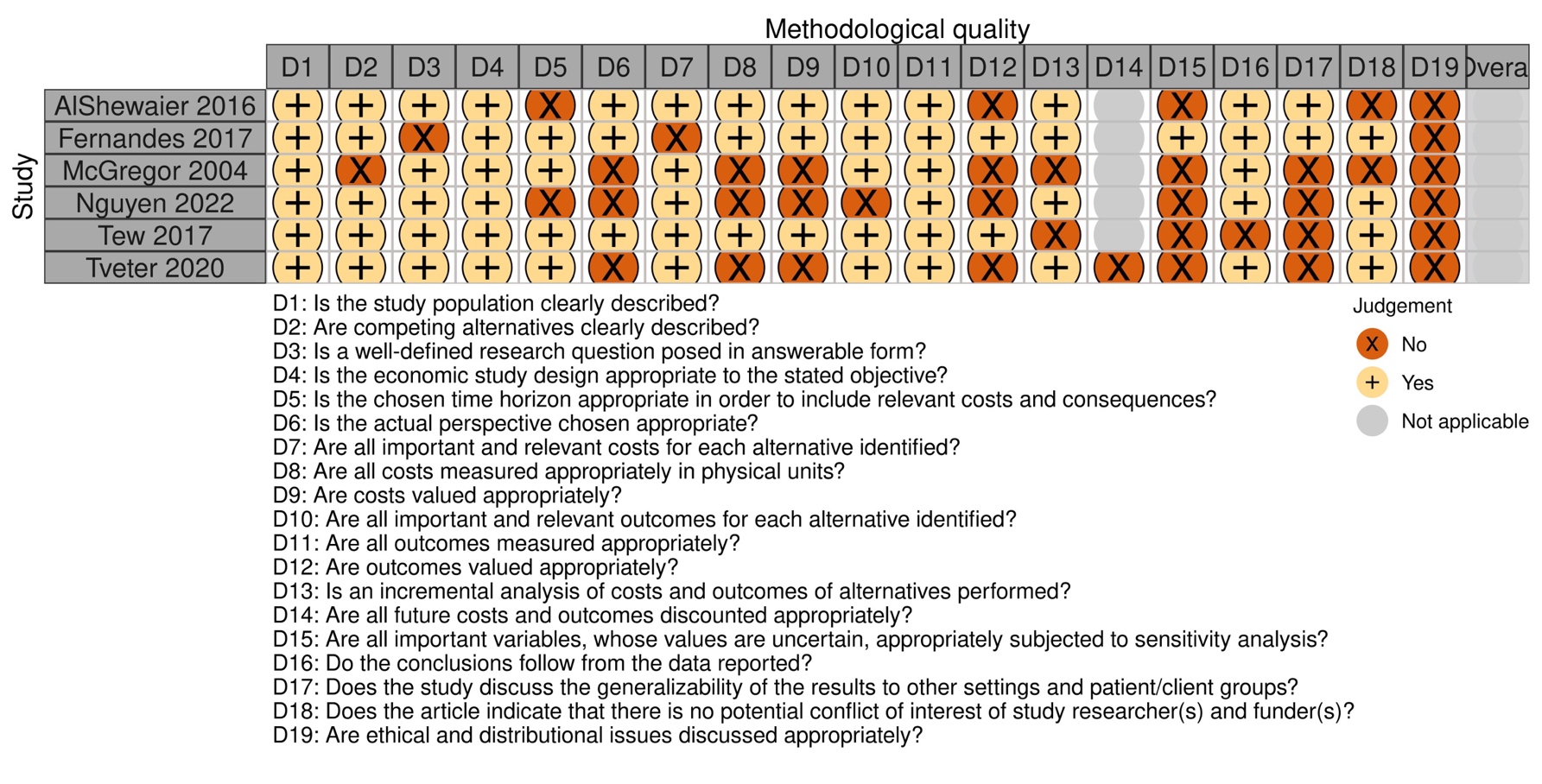


Figure 3: Methodological quality of full trial-based economic evaluations as judged with the CHEC-checklist


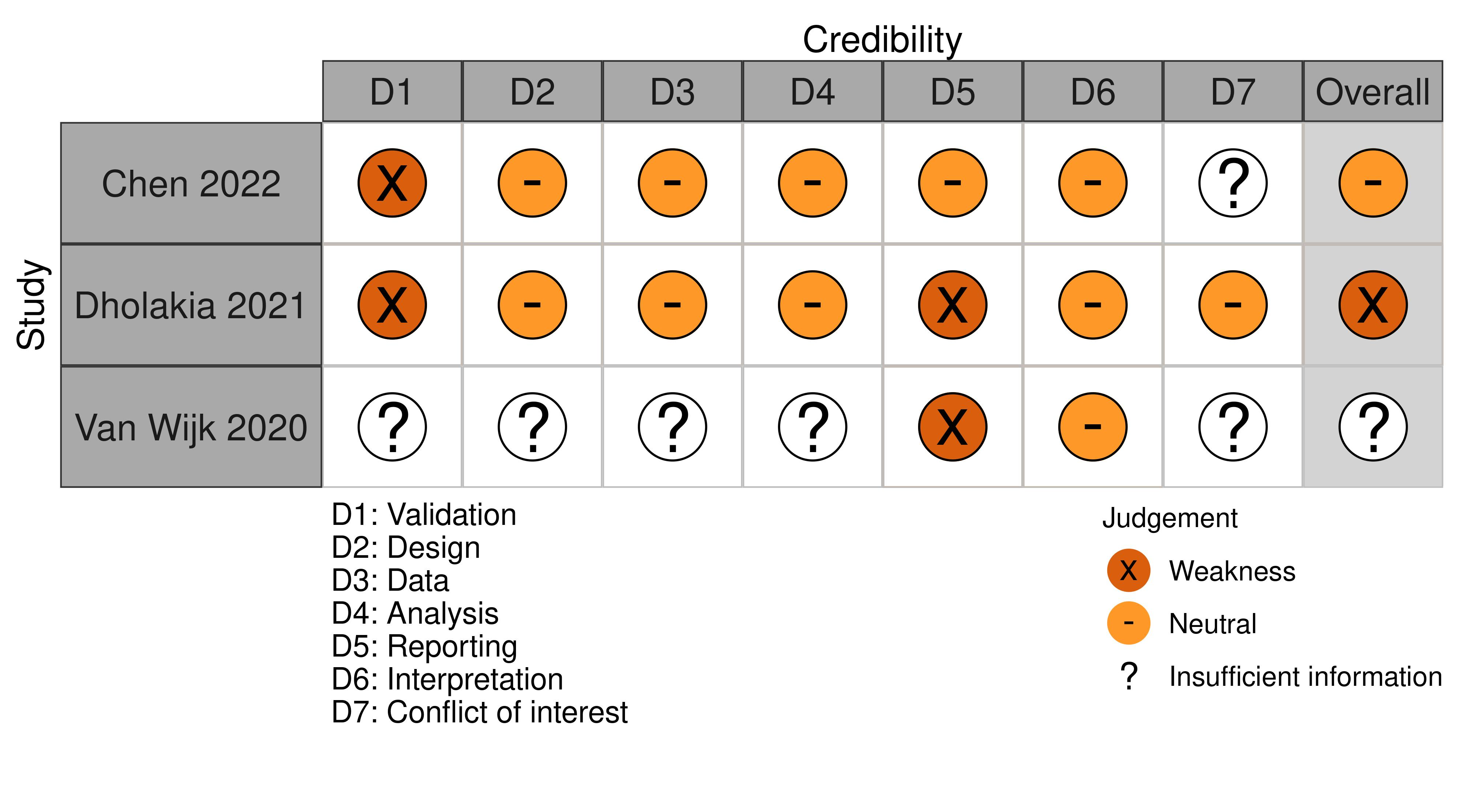


Figure 4: Credibility of model-based economic evaluations as judged with the ISPOR-checklist

### Appendix 11 – Detailed costs results of included economic evaluations

| **Study ID, main reference** | **Quantities of resource use (IG vs. CG)** | **Unit costs (IG vs. CG)** | **Total costs in original currency and year (IG vs. CG)** | **ICER in original currency and year (full EEs only)** | **Study authors’ conclusion (full EEs only)** |
| --- | --- | --- | --- | --- | --- |
| **Results from model-based economic evaluations** | | | | | |
| **Results from CEAs** | | | | | |
| Dholakia 2021 (41) | Hospital charge without major complication: 66,021 vs. 66,021  Hospital charge with major complication: 152,596 vs. 152,596  Non-home discharge facility charge (after peri-operative complication): 1,925 vs. 3,850  Non-home discharge facility charge (no peri-operative complication): 1,100 vs. 1,925  Usual preoperative care: 200 vs. 200  Prehabilitation costs: 100 vs. 0 | | Mean 84,053 vs. 91,713; MD* -7,660  (US dollar, year not reported; assumed 2021) | Not calculated as intervention dominated control. | "Our cost-effectiveness model provides a financial lens on the potential impact of prehabilitation on healthcare costs. Based on our use of conservative estimates, the magnitude of cost savings may even exceed our projections in this analysis. Further investigation into prehabilitation holds promise not only to provide better care for at-risk patients, but to decrease healthcare system costs and improve system efficiency." |
| **Results from CBAs** | | | | | |
| Chen 2022 (40) | Prehabilitation:  Medical director 0.2 full-time equivalents (FTE)  Program management 0.4 FTE  Clinical coordinator 1.0 FTE  Administrative assistant 0.5 FTE  Qualified exercise professional 0.4 FTE  Dietitian 0.25 FTE  Psychologist 0.25 FTE  Morbidity:  Complications: 16 vs. 24 | Prehabilitation:  Not reported  Morbidity: 52,555.77 standard median morbidity cost | Mean 4,623* vs. 5,255*; MD* -632  (US dollar, 2021) | ICER not reported (Total cost-benefit: 151,706 | "The financial forecasting in this article represents relevant insight for key decision-makers responsible for surgical quality improvement initiatives who will be increasingly called upon regarding the value of integrating prehabilitation into clinical care models. Ultimately, future work establishing prehabilitation cost-benefit and cost-effectiveness grounded in program evaluations based on real-world experiences are essential next steps to translating prehabilitation research into a standard of care clinical service." |
|  | Prehabilitation costs: 1,119.75 (calculated), consisting of,  Fixed costs (total sums)  Medical director 38,000  Program management 40,000  Clinical coordinator 83,000  Administrative assistant 25,000  Qualified exercise professional 29,000  Dietitian 12,000  Psychologist 22,000  Sundries and other expenses 1,500  Variable costs (cost per patient)  Patient manual 5  Exercise equipment 30  Protein supplements 35  IMT device (prorated at 20%) 6  Smoking cessation NA  Morbidity costs: -420,446 | |  |  |  |
| Van Wijk 2020 (54) | Prehabilitation costs: 1,576 | | 30,511 vs. 32,953; Difference -2,442†  (Euro (Netherlands), year not reported; assumed 2020) | ICER not reported/ calculated as intervention dominates control | "[T]he model showed that a multimodal prehabilitation program for pancreatic cancer surgery is financially beneficial with a ROI of 1.55." |
| **Results from trial-based economic evaluations** | | | | | |
| **Results from CUAs** | | | | | |
| AlShewaier 2016 (37) | Prehabilitation costs: median 1,512 vs. 0  ACL reconstruction and physiotherapy after surgery: 36,000 vs. 36,000 | | Median 37,512 vs. 36,000; Difference in medians 1,512  (Saudi Riyals, 2015) | 14,264.15/QALY | "Overall, patients from the Intervention group showed better primary and secondary health outcomes than the Control group and the ICER indicated that the intervention cost about £2241 per QALY gained due to the intervention. The clinical and cost-effectiveness of the intervention indicated by the present clinical trial suggest that pre-operative physiotherapy is beneficial for the management of ACL-deficient patients and should be introduced to healthcare management of ACL injury in KSA." |
| Fernandes 2017 (42) | Prehabilitation:  Exercise sessions: mean 13.1 (range 0 to 24) vs. 0  Primary health care, visits: mean 21.4 ± BS SE 1.3 vs. 23.7 ± 1.7  Secondary health care, visits: mean 8.6 ± BS SE 0.9 vs. 9.0 ± 1.1  Secondary health care, inhospital days: mean 4.8 ± BS SE 0.7 vs. 7.2 ± 1.8  Patient expenses:  Informal time per exercise session: 1.25 h vs. 0  Travel distance (km): mean 21.5 (range 0.5 to 78.3) vs. 0  Patient expenses:  Informal time per exercise session: 1.25 h vs. 0  Travel distance (km): mean 21.5 (range 0.5 to 78.3) vs. 0 | Prehabilitation unit costs (calculated from Tables 3 and 4):  25 vs. 0* per session  Primary health care costs: 25 vs. 27* per visit  Secondary health care costs: 262 vs. 217* per visit  Secondary health care costs for inhospital days: 2,724 vs. 1,908* per day  Patient expenses:  Informal time total costs: mean 302 ± SE 15.3 vs. 0  Travel costs per distance: 0.51 EUR/km vs. 0  Total travel costs: mean 137 ± SE 12.2 vs. 0 | Mean 16,181 ± BS SE 1,174 vs. 16,313 ± BS SE 1374; MD -132 (95%-CI -3,668 to 3,405) (+307 when including patient expenses)  (Euro (country not reported), 2012) | ICER not reported/ calculated as intervention dominates control  When including patients’ expenses (calculated): 7,675 per QALY gained (EUR 2012); 8,268 EUR (2020) per QALY gained  Probability of cost-effectiveness-acceptability at a willingness-to-pay of 40,000 EUR: 80% | "Preoperative supervised neuromuscular exercise for 8 weeks was found to be cost-effective in patients scheduled for THR and TKR surgery at conventional thresholds for willingness to pay. One-year clinical effects were small to moderate and favoured the intervention group, but only statistically significantly so for quality of life measures." |
|  | Prehabilitation costs: Mean 326 (Bootstrap SE 13) vs. 0  Primary health care costs: 530 (35) vs. 629 (74)  Secondary health care costs: 2,252 (557) vs. 1,949 (316)  Secondary health care costs for inhospital days: 13,074 (706) vs. 13,735 (1,249)  Patient expenses: 439 vs. 0 | |  |  |  |
| Nguyen 2022 | Prehabilitation cost: 62 vs. 0  Initial admission: Mean 9,168 ± SD 1,568 vs. 9,065 ± 1,230  Rehabilitation: Mean 6,143 ± SD 7,005 vs. 6,693 ± 5,875  Adverse events: Mean 200 ± SD 919 vs. 229 ± 1,223 | | Mean 15,573 ± SD 7,247 vs. 15,987 ± SD 6,519; MD -414  (Euro (France), year not reported; assumed 2016) | ICER not reported/ calculated as intervention dominates control | "We found no evidence that our intervention improved any of the secondary end points (ie, pain, quality of life, level of physical activity, and costs)." (Discussion) |
| Tveter 2020 (53) | Patients who underwent operation: 22/90 (24.4%) vs. 33/90 (36.7%) | Not reported | Not reported per group; Difference -500†  (Euro (country not reported), year not reported; assumed 2015) | ICER not reported/ calculated as intervention dominates control | "The Results in this within-trial analysis indicate that multimodal occupational therapy in the waiting period before surgical consultation compared to usual care is a cost-effective alternative taking a health care perspective." |
| **Results from CEAs** | | | | | |
| McGregor 2004 (47) | Mean days in the hospital: 15 vs. 18  Inpatient physiotherapy: “Little difference was seen between the groups with respect to inpatient physiotherapy”  Inpatient occupational therapy: “Patients in group B required more occupational therapy” | Days in the hospital: not reported  Inpatient physiotherapy: not reported  Inpatient occupational therapy: 11 | Mean 2,842 vs. 3,429; MD* -587  (Great Britain Pound, 2003) | ICER not reported/ calculated as intervention dominates control | "One of the key findings is the positive impact of a preoperative educational program on the costs of treatment. One of the main cost savings was reduction in length of hospital stay, which will help relieve the current shortage of hospital beds in the United Kingdom. Furthermore, a reduction in length of hospital stay can have a positive effect on the overall costs of health care." |
|  | Prehabilitation costs: mean 14.58 vs. 0 | |  |  |  |
| Tew 2017 (52) | Prehabilitation:  Polar heart rate watches and chest straps: 6 vs. 0  Cycle ergometers: 6 vs. 0  Nurse time: 12 hours per patient for 27 patients vs. 0  Nurse time maintenance sessions: 36 vs. 0  Physiotherapist time: 12 hours per patient for 27 patients vs. 0  Physiotherapist time maintenance sessions: 36 vs. 0  Travel costs: 12 return journeys per patient for 27 patients vs. 0  Costs of AAA repair:  Open surgeries: 11 vs. 11  Endovascular surgeries: 16 vs. 15 | Polar heart rate watches and chest straps: 63.67  Cycle ergometers: 228  Nurse time: 30.42/hour or maintenance session  Physiotherapist time: 30.42/hour or maintenance session  Travel cost: 25/patient  Costs of AAA repair:  Open: 8,285.56  Endovascular: 12,675.50 | BS Mean 12, 519 ± SD 3,107 vs. 12,009 ± 3,107; BS MD 510 (95%-CI -1,393 to 2,498)  (Great Britain Pound, 2015/16) | ICER not reported | "Regardless of the optimal timing and content of a preoperative exercise programme for this population, it seems that a large, multicentre trial that is pragmatic in design and explores both clinical effectiveness and cost-effectiveness is needed before recommendations can safely be made about whether or not the healthcare systems should adopt this type of intervention." |
|  | Prehabilitation costs: 1,176 vs. 0  Costs of AAA repair: Mean 10,481 ± SD 2,247 vs. 10,880 ± 2,209  Postdischarge costs: Mean 862 ± SD 2,653 vs. 1,129 ± 2,290, consisting of  GP contact at practice: 32 ± 46 vs. 30 ± 37  GP home visit: 7 ± 16 vs. 18 ± 29  GP telephone call: 13 ± 26 vs. 10 ± 26  Practice Nurse contact: 24 ± 58 vs. 11 ± 17  District/Community nurse: 479 ± 1,809 vs. 44 ± 83  Physiotherapy contact: 6 ± 24 vs. 17 ± 68  Occupational therapy contact: 0 vs. 20 ± 64  Dietician contact: 0 vs. 4 ± 18  Hospital out-patient attendance: 417 ± 1,005 vs. 269 ± 200  Accident and emergency attendance: 0 vs. 12 ± 38  Hospital in-patient admission: 0 vs. 693 ± 2,280  Social worker contact: 4 ± 18 vs. 4 ± 17 | |  |  |  |
| **Results from CCAs** | | | | | |
| Barberan-Garcia 2019 (38) | Patients attended at the emergency room: 13/62 (21.0%) vs. 14/63 (22.2%)  Number of emergency room visits per patient: mean 1.0 ±  SD 0 vs. 1.3 ± 0.5  Patients readmitted or still hospitalized: 5/62 (8.1%) vs. 5/63 (7.9%)  Readmission length of stay, days: mean 7 ± SD 6 vs. 6 ± 6  Surgical reinterventions: 0/62 (0%) vs. 0/63 (0%) | Not reported: “We want to point out that costs at 3 and 6 months have not been reported because of the lack of differences on healthcare use between groups during this period of the follow-up” | Mean 4,613 (range 1,523 to 25,948) vs. 5,453 (1,349 to 29,338); BS MD -812 (95%-CI -2,642 to 878)  (Euro (Spain), 2017) | Not applicable | Not applicable |
|  | Prehabilitation costs (mean): 389 vs. 0, consisting of  Cardiopulmonary exercise testing: 230 vs. 0  Motivational interview: 41 vs. 0  Pedometer device 22 vs. 0  Group endurance-exercise training sessions: 96 vs. 0 | |  |  |  |
| Gao 2015 (43) | Prehabilitation costs: mean 1,013.30 vs. 0 | | Mean 40,131.72 ± SD 4,663.28 vs. 36,943.33 ± 3,663.28; MD* 3,188.39  (Renminbi Yuan, year not reported; assumed 2011) | Not applicable | Not applicable |
| Gränicher 2020 (44) | Prehabilitation costs: mean 467 vs. 0  Postoperative physiotherapy: mean 1,161 vs. 1,223  In-patient rehabilitation: mean 3,645 vs. 5,481 | | Mean 5,273 vs. 6,704; MD* -1,431  (Swiss franc, 2017) | Not applicable | Not applicable |
| Howard 2019 (30) | Prehabilitation costs: 100 vs. 0 | | Mean 75,493.97 ± SD 55,151.21 vs. 97,439.88 ± 100,377.06; MD* (incorporating prehabilitation costs) -21,945.91  (US dollar, 2012-2017‡) | Not applicable | Not applicable |
| Huang 2012 (45) | Prehabilitation costs: 360 vs. 0 | | Mean 123,726 ± SD 5,204 vs. 125,838 ± 4,428; MD* (incorporating prehabilitation costs) -1,752  (New Taiwan Dollar, 2008-2010) | Not applicable | Not applicable |
| Koh 2021 (46) | Median LOS (days): 9 (range 4 to 32) vs. 11 (5 to 84) | National average unsubsidized cost per day for a colonic resection in a public hospital (sum not reported) | MD -3,482  (US dollar, 2019) | Not applicable | Not applicable |
| Lai 2017 (32) | Material cost: mean 3,237.6 ± SD 1,088.1 vs. 3,640.8 ± 1,632.2  Drug cost: mean 1,235.5 ± SD 564.5 vs. 1,817.6 ± 1,443.8  Costs for daily nursing care fee, intraoperative examination cost, and surgery-related expenses not reported. | | Mean 7550.7 ± SD 1,351.9 vs. 8,466.4 ± 2,441.2; MD* -915.7  (Euro (country not reported), year not reported; assumed 2015) | Not applicable | Not applicable |
| Lai 2019 (33) | Material cost: median 23,350.8 (IQR 18,300.6 to 26,421.9) vs. 25,730.0 (21,328.7 to 29,250.2)  Drug cost: median 7,230.0 (IQR 6,661.9 to 8,347.4) vs. 11,388.6 (7,963.0–16,314.3)  Remaining in-hospitalization costs not reported. | | Median 48,588.7 (IQR 44,999.1 to 52,693.3) vs. 52,445.3 (49,002.9 to 61,994.0); Difference in medians* -3856.6  (Renminbi Yuan, year not reported; assumed 2018) | Not applicable | Not applicable |
| Ploussard 2020 (50) | Prehabilitation costs: Mean 250 vs. 0 Hospitalization costs for personnel, materials and devices, and readmission: Mean 2,894 vs. 3,554 | | Mean 3,144 vs. 3,554; MD* -410  (Euro (France), 2020) | Not applicable | Not applicable |
| Risco 2022 (51) | Prehabilitation cost: 382 vs. 0 Hospitalization: 3989 vs. 4181 Treatment: 468 vs. 455 Diagnostic Tests: 325 vs. 343 Catering: 182 vs. 199 Structure: 395 vs. 424 Readmissions and Emergency Room: 519 vs. 533 | | Mean 6,260 vs. 6,135; MD 125  (Euro (Spain), 2017-2019) | Not applicable | Not applicable |
| Wang 2020 (55) | Not reported | | Median 6,892 (IQR 5,153 to 9,916) vs. 8,251 (5,982 to 12,379); Difference in medians -1,359  (Currency not reported; assumed Singapore dollar, year not reported; assumed 2017) | Not applicable | Not applicable |
| Zhou 2017 (34) | Prehabilitation costs: mean 142.0 ± SD 32.5 vs. O Preoperative ward expense:  mean 118.4 ± SD 34.7 vs. 115.3 ± 32.0 Preoperative care expense:  mean 53.3 ± SD 15.6 vs. 51.9 ± 14.4 Surgery related expense:  mean 5,602.5 ± SD 2,211.1 vs. 5,751.0 ± 1,495.5 Postoperative drug expense:  mean 379.1 ± SD 211.3 vs. 444.8 ± 201.0 Postoperative ward expense:  mean 112.0 ± SD 57.0 vs. 131.0 ± 100.4 Postoperative care expense:  mean 67.2 ± SD 35.9 vs. 78.6 ± 60.2 | | Mean 7,131.8 ± SD 2,316.6 vs. 77,266.4 ± 1,615.0; MD* -134.60  (Currency not reported; assumed Singapore dollar, year not reported; year could not be assumed) | Not applicable | Not applicable |
| **Results from CMAs** | | | | | |
| Beaupre 2004 (39) | Acute care length of stay (days): mean 6.7 ± SD 2.2 vs. 7.3 ± 2.5 Patients transferred for subacute rehabilitation: 23/55 (41.8%) vs. 31/60 (51.6%) Subacute rehabilitation length of stay (days): mean 7.7 ± SD 2.0 vs. 7.7 ± 2.8 Patients readmitted: 5/55 (9.1%) vs. 6/60 (10.0%) Readmission length of stay (days): mean 3.4 ± SD 0.55 vs. 3.8 ± 2.0 Total length of stay for acute care, subacute rehabilitation, and readmissions (days): mean 10.2 ± SD 4.5 vs. 11.7 ± 5.2 | Unit costs not reported; total costs for acute care, subacute rehabilitation, and readmissions: mean 878 ± SD 1,233 vs. 1,090 ± 1,316 | Mean 1,369 ± SD 1,274 vs. 1,366 ± 1,415; MD 3  (Canadian dollars, 1997/98) | Not applicable | Not applicable |
|  | Prehabilitation costs: 240 vs. 0 Homecare costs: mean 127 ± SD 177 vs. 117 ± 159 Community rehabilitation costs: mean 125 ± SD 226 vs. 159 ± 251 | |  |  |  |
| Englesbe 2017 (29) | Prehabilitation costs: 100 vs. 0  Cost from primary encounter: median 18,681 (IQR 12,078 to 27,968) vs. 25,523 (16,697 to 42,459) | | Provider perspective:  Median 21,244 (IQR 12,774 to 38,170) vs. 29,026 (18,847 to 49,045); Difference in medians* -7,728  Payer perspective:  Median 24,155 (IQR 15,237 to 42,538) vs. 30,821 (21,441 to 46,815); Difference in medians* -6,666  (US dollar, 2015) | Not applicable | Not applicable |
| Mouch 2019 (31) | Prehabilitation costs: 70 vs. 0  Index hospitalisation payment: mean 19,195 ± SD 11,309 vs. 21,380 ± 19,972  Inpatient professional fees: mean 2,685 ± SD 1,821 vs. 2,818 ± 1,962  Readmission payment: mean 4,467 ± SD 14,431 vs. 4,563 ± 12,219  Post-acute care payment:  Outpatient care: mean 2,054 ± SD 3,561 vs. 2,093 ± 3,926  Outpatient professional fees: mean 595 ± SD 2,142 vs. 595 ± SD 2,142  Skilled nursing facility: mean 941 ± SD 3,408 vs. 1566 ± 4,927  Home health services: mean 829 ± SD 1,418 vs. 960 ± 1,453 | | Mean 31,641 ± SD 25,930 vs. 34,837 ± 32,288; MD* -3,196  (US dollar, year not reported; assumed 2017) | Not applicable | Not applicable |
| Pham 2016 (49) | Length of stay (days): mean 2.60 ± SD 0.82 days vs. 2.89 ± 0.76 Patients who underwent operation: 24/29 (82.8%) vs. 21/21 (100%) | Recovery cost per day: 1,144.01 Operating room costs: 4,719.04 | Reported only for a subset of patients (5/29 vs. 11/21): Mean 7,791.00 ± SD 458.24 vs. 7,898.73 ± 1004.83; MD* -107.73  (Canadian dollar, year not reported; assumed 2017) | Not applicable | Not applicable |
|  | Prehabilitation costs: 157.80 vs. 0  12-week pool-based class/patient: 67.80 vs. 0  12-week land-based class/patient: 90.00 vs. 0 | |  |  |  |
| * Calculated by review authors  † Measure of central tendency (mean, median) not reported  ‡ In this study, costs were taken from the year they occurred; as we did not have access to the patient-level data, the last was chosen for cost conversion.  Abbreviations: BS, bootstrapped; CBA, cost-benefit analysis; CCA, cost-consequence analysis; CEA, cost-effectiveness analysis; CG, control group; CMA, cost-minimisation analysis; CUA, cost-utility analysis; IG, intervention group; ICER, incremental cost-effectiveness ratio; IQR, interquartile range; MD, mean difference; SD, standard deviation; US, United States of America. | | | | | |

### Appendix 12 – Results of adherence and safety outcomes

| **Study ID, main reference** | **Adherence (% of all scheduled sessions)** | **Adherence (% of participants who achieved sufficient adherence)** | **Safety/Feasibility of prehabilitation program** |
| --- | --- | --- | --- |
| AlShewaier 2016 (37) | not reported | not reported | "No adverse effects to pre-operative physiotherapy which required medical attention were experienced by the patients during this study." |
| Barberan-Garcia 2019 (38) | not reported | not reported | "No intervention patient reported any relevant incidence [incidents?] during the prehabilitation period." |
| Beaupre 2004 (39) | 777/780 (99.6%)* | 64/65 (98.5%) completed all 12 treatment sessions | not reported |
| Chen 2022 (40) | not reported | not reported | not reported |
| Dholakia 2021 (41) | not reported | not reported | not reported |
| Englesbe 2017 (29) | not reported | Before matching: 436/535 (81.5%) were engaged in the program | not reported |
| Fernandes 2017 (42) | not reported | 62/84 (73.8%) had attendance of 12 sessions or more | 1/84: "One patient with hip OA discontinued the exercise intervention after experiencing an increase in pain" |
| Gao 2015 (43) | not reported | not reported | not reported |
| Gränicher 2020 (44) | not reported | 8/10 (80.0%) completed all (n = 9) sessions | "[…] no adverse events were reported within the study." |
| Howard 2019 (30) | not reported | 70% of MSHOP patients complied with the program | not reported |
| Huang 2012 (45) | not reported | not reported | not reported |
| Koh 2021 (46) | not reported | not reported | not reported |
| Lai 2017 (32) | not reported | 45/51 (88.2%) completed the 7-day rehabilitation plan (rest were drop-outs) | "2 requested withdrawal because they felt they could not endure the regimen." |
| Lai 2019 (33) | not reported | 32/34 (94.1%) completed the 7-day rehabilitation plan (rest were drop-outs) | "2 IG patients did not complete the training program, as they could not endure the training intensity (one stopped after the third training day and one at the fourth)." |
| McGregor 2004 (47) | not reported | not reported | 4 patients in the IG lost to follow-up with no explanation vs. none in the CG |
| Mouch 2019 (31) | not reported | 324/523 (62.0%) entered step counts 3 or more times per week for at least 50% of weeks enrolled | "A total of 39 inpatients (7.5%) disenrolled from the MSHOP program (they asked to be removed) […]." |
| Nguyen 2022 (48) | not reported | 45/131 (34.4%) attended all sessions | At least 1 minor event: 48/131 (37%) vs. 47/131 (36%) At least 1 serious adverse event: 18/131 (14%) vs. 12/131 (9%) |
| Pham 2016 (49) | 10/12 (83.3%) sessions attended on average per patient | not reported | not reported |
| Ploussard 2020 (50) | not reported | approximately 75% | not reported |
| Risco 2022 (51) | not reported | 112/328 (34%) were considered completers | not reported |
| Tew 2017 (52) | 276/364 (75.8%) | 17/27 (63.0%) completed at least 75 per cent of the main-phase sessions (at least 9 of 12 sessions) plus all weekly maintenance sessions if surgery was delayed. | 1/27: "One adverse event occurred that resulted in the termination of an exercise session: a single episode of short-lived angina that was relieved by self-administration of glyceryl trinitrate." |
| Tveter 2020 (53) | mean of 77% (SD 30.2) of total possible exercise sessions, mean of 69% (SD 33.1) of days using day orthoses, mean of 64% (SD 36.7) of days using night orthoses | 58/90 (64.4%) treatment adherence overall, i.e. fulfilling a minimum of three of the following four treatment elements during the first 3 months: 72/90 (80.0%) adherent to exercise (two exercise sessions recorded per week, for at least 8 weeks), 61/90 (67.8%) adherent to day orthoses (use of a day orthosis for at least 4 days per week, for a minimum of 8 weeks, regardless of the number of hours used per day), 49/90 (54.4%) adherent to night orthoses (use of a night orthosis for at least 5 hours per night for a minimum of 5 days a week for at least 8 weeks), use of at least three of the five assistive devices, reported at the 4-month followup. | Minor adverse events: 17/90: "During the intervention period, 17 patients (19%) experienced minor or moderate pain or discomfort with the orthoses and/ or hand exercises (online supplementary file, Adverse events). Few problems were reported for using the assistive devices (median score 0, IQR 0–2)." "[…] no serious adverse events […]." |
| Van Wijk 2020 (54) | not reported | not reported | not reported |
| Wang 2020 (55) | not reported | not reported | not reported |
| Zhou 2017 (34) | not reported | 160/197 (81.2%) completed the 7-day intensive programme | 11/197 (5.6%) could not endure the high-intensive regimen |
| * Calculated by review authors | | | |

### Appendix 13 – Results of descriptive post-hoc subgroup analyses to explore heterogeneity in cost-effectiveness results

| **Domain** | **Count of economic evaluations that found prehabilitation cost-effective** | **References** |
| --- | --- | --- |
| All economic evaluations (reference) | 16/25 (64.0%) | (29, 31-34, 38, 40-42, 46-50, 53, 55) |
| ***Population*** | | |
| Continent |  |  |
| Asia | 5/8 (62.5%) | (32-34, 46, 55) |
| Europe | 6/10 (60.0%) | (38, 42, 47, 48, 50, 53) |
| North America | 5/7 (71.4%) | (29, 31, 40, 41, 49) |
| Disease/Surgery type |  |  |
| Cancer | 7/8 (87.5%) | (32-34, 41, 46, 50, 55) |
| Orthopaedic | 5/9 (55.6%) | (42, 47-49, 53) |
| Mixed major/other | 4/8 (50.0%) | (29, 31, 38, 40) |
| Perioperative risk |  |  |
| High | 8/11 (72.7%) | (31, 32, 34, 38, 40, 41, 46, 49) |
| Normal/low | 8/14 (57.1%) | (29, 33, 42, 47, 48, 50, 53, 55) |
| ***Intervention*** | | |
| Programme type |  |  |
| Multimodal programme | 14/20 (70.0%) | (29, 31-34, 38, 40, 42, 46, 48-50, 53, 55) |
| Unimodal/variable programme | 2/5 (40.0%) | (41, 47) |
| Setting |  |  |
| Home | 5/7 (71.4%) | (29, 31, 47, 50, 53) |
| Outpatient | 6/11 (54.5%) | (38, 40, 42, 46, 48, 49) |
| Inpatient | 3/4 (75.0%) | (32-34) |
| Not reported/variable | 2/3 (66.7%) | (41, 55) |
| Duration |  |  |
| ≤ 4 weeks* | 10/13 (76.9%) | (29, 31-34, 40, 46, 47, 50, 55) |
| > 4 weeks | 5/10 (50.0%) | (38, 42, 48, 49, 53) |
| variable | 1/2 (50.0%) | (41) |
| Intensity of exercise element |  |  |
| High/moderate to high | 3/7 (42.9%) | (38, 49, 53) |
| Low/moderate to low | 2/3 (66.7%) | (40, 48) |
| Not applicable/not reported | 11/15 (73.3%) | (29, 31-34, 41, 42, 46, 47, 50, 55) |
| Evidence-based programme |  |  |
| Yes | 5/8 (62.5%) | (38, 40, 48, 49, 53) |
| No | 11/17 (64.7%) | (29, 31-34, 41, 42, 46, 47, 50, 55) |
| Costs |  |  |
| ≤ 103 EUR (2020)* | 5/7 (71.4%) | (29, 31, 47-49) |
| > 103 EUR (2020) | 4/11 (36.4%) | (38, 40, 42, 50) |
| Not reported/calculable or variable | 7/7 (100.0%) | (38, 40, 42, 50) |
| ***Economic evaluation methods*** | | |
| Type of economic evaluation |  |  |
| Model-based | 2/3 (66.7%) | (40, 41) |
| Trial-based | 14/22 (63.6%) | (29, 31-34, 38, 42, 46-50, 53, 55) |
| Study type (trial-based economic evaluations only) |  |  |
| Randomised controlled trial | 8/13 (61.5%) | (32, 33, 38, 42, 47-49, 53) |
| Non-randomised trial | 6/9 (66.7%) | (29, 31, 34, 46, 50, 55) |
| Analysis type |  |  |
| Cost-utility analysis | 3/4 (75.0%) | (42, 48, 53) |
| Cost-effectiveness analysis | 2/3 (66.7%) | (41, 47) |
| Cost-benefit analysis | 1/2 (50.0%) | (40) |
| Cost-consequence analysis | 7/12 (58.3%) | (32-34, 38, 46, 50, 55) |
| Cost-minimisation analysis | 3/4 (75.0%) | (29, 31, 49) |
| Perspective |  |  |
| Mix of payer/provider perspective | 7/9 (77.8%) | (29, 31, 38, 42, 47, 48, 50) |
| Payer or provider or patient perspective | 3/7 (42.9%) | (40, 41, 46) |
| Unclear perspective | 6/9 (66.7%) | (32-34, 49, 53, 55) |
| Time horizon |  |  |
| < 3 months* | 9/15 (60.0%) | (32-34, 38, 40, 46, 48, 50, 55) |
| ≥ 3 months | 7/10 (70.0%) | (29, 31, 41, 42, 47, 49, 53) |
| ***Other*** | | |
| Funding |  |  |
| None/non-profit | 10/16 (62.5%) | (29, 31, 32, 34, 38, 42, 47, 48, 50, 53) |
| Not reported/unclear | 6/9 (66.7%) | (33, 40, 41, 46, 49, 55) |
| Conflict of interest |  |  |
| No (relevant) conflict of interest | 11/16 (68.8%) | (32-34, 38, 40, 41, 46, 48, 50, 53, 55) |
| Relevant conflict of interest or not reported | 5/9 (55.6%) | (29, 31, 42, 47, 49) |
| *Cut-off was determined arbitrarily. | | |
